# Trifluoromethylthio and Trifluoromethyl Functionalization of Endomorphin‑1 Enhances its Hydrophobicity and Plasma Stability while Preserving its Affinity for the μ‑Opioid Receptor

**DOI:** 10.1021/acs.joc.6c00321

**Published:** 2026-04-15

**Authors:** Jure Gregorc, Jolien De Neve, Karine Guitot, Brian J. Holleran, Nathalie Lensen, Louis Gendron, Thierry Brigaud, Jernej Iskra, Steven Ballet, Grégory Chaume

**Affiliations:** † University of Ljubljana, Faculty of Chemistry and Chemical Technology, Večna pot 113, Ljubljana 1000, Slovenia; ‡ 27004 CY Cergy Paris Université, CNRS, BioCIS, Cergy Pontoise 95000, France; § Université Paris-Saclay, CNRS, BioCIS, Orsay 91400, France; ∥ Research Group of Organic Chemistry, Departments of Chemistry and Bioengineering Sciences, 70493 Vrije Universiteit Brussel, Brussels B-1050, Belgium; ⊥ Institut de Pharmacologie de Sherbrooke, Department of Pharmacology and Physiology, Faculty of Medicine and Health Sciences, Université de Sherbrooke, 3001, 12e Avenue Nord, Sherbrooke, Quebec J1H 5N4, Canada

## Abstract

The incorporation of fluorinated amino acid residues into peptides represents a promising strategy for improving the pharmacokinetic properties of bioactive peptides. Herein, we report the solid-phase synthesis (SPPS) of a set of ten SCF_3_- or CF_3_-modified neuropeptide analogs based on endomorphin-1 (EM1). EM1, a selective subnanomolar μ-opioid receptor (μOR) agonist with poor metabolic stability (*t*_1/2_ = 6 min in human plasma), was selected as a model to evaluate the effects of SCF_3_ and CF_3_ functionalization on the pharmacokinetic profile of short bioactive peptides. The syntheses of eight ready-to-use SCF_3_- or CF_3_-containing building blocks for SPPS and their incorporation into peptides are reported. *In vitro* μOR binding and functional activity assays demonstrated that most fluorinated analogs retained binding affinity and potency, accompanied by increased hydrophobicity. Among the series, modification of the pharmacophoric Tyr^1^ residue with l-Dmt­(3-SCF_3_) yielded the most favorable profile (*K*_*i*_ = 1.4 nM, EC_50_ = 0.9 nM). Plasma stability studies revealed a significant increase in half-life for this ligand (72-fold relative to EM1 and 14-fold relative to EM1­(Dmt^1^)), and thus, further demonstrated the potential of SCF_3_-containing amino acids in therapeutic peptide design.

## Graphical abstract

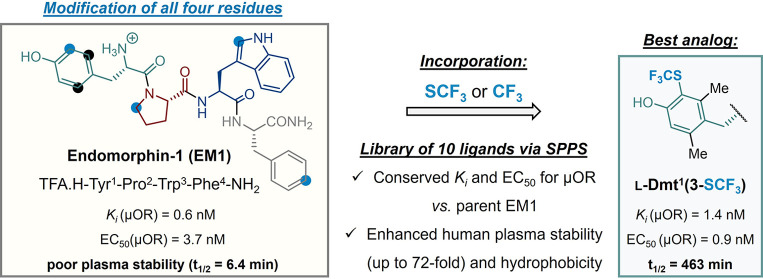

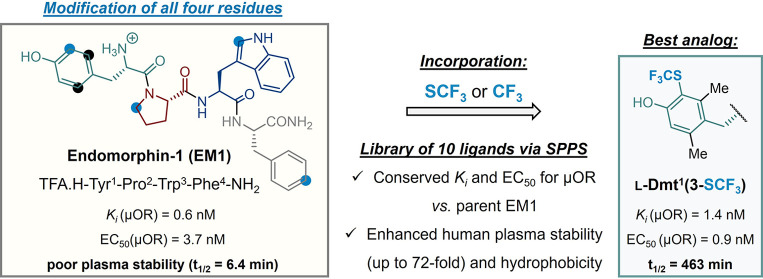

## Introduction

Synthetic modified peptides are a valuable therapeutic modality because of their highly selective interactions at large surface area binding sites, next to their high potency, tolerability, and structural versatility.^1^ The endogenous peptidome serves as a straightforward source of lead compounds for further peptide drug optimization, addressing the typically limited pharmacokinetic properties of native sequences.^2,3^ In the context of neuropeptides, several subtypes of endogenous opioid peptides, such as enkephalins, dynorphins, endorphins, and endomorphins, have been studied.^4^ These peptides act as selective agonists of specific opioid receptors (ORs), the μ-opioid receptor (μOR), δ-opioid receptor (δOR), κ-opioid receptor (κOR), and nociceptin receptor (NOPR). They exhibit strong antinociceptive effects, in addition to other physiological roles.^5^ Specifically, endomorphin-1 (EM1) and endomorphin-2 (EM2) stand out as the most selective μOR agonists, displaying excellent affinity (EM1: *K*_*i*_ of 360 pM for μOR) and potent analgesia.^6^ Nevertheless, small-molecule μOR agonists that mimic opioid peptide function, such as morphine or fentanyl, remain the gold standard in current pain relief therapy, despite their adverse side-effects (e.g., acute tolerance, physical dependence, respiratory depression, etc.).^5^ Clinical use of EM-based analgesics has been severely limited by their inherent pharmacokinetic restrictions, including short duration of action, inability to effectively cross the blood–brain barrier (BBB), and low plasma stability.^7,8^ To mitigate these limitations, structure modifications have been employed, such as cyclization,^9–11^ conjugation (e.g., glycosylation and lipidation),^12^ and incorporation of d-amino acids, β-amino acids, or other unnatural amino acids.^13–19^ A less explored avenue for optimizing drug-like properties of neuropeptides is the incorporation of fluorinated amino acids (FAAs).^10,11,20,21^

In general, FAA incorporation into peptide sequences has been shown to lead to improved proteolytic stability,^22,23^ membrane permeability *via* enhanced hydrophobicity,^24,25^ receptor affinity and selectivity,^11^ or improved self-assembly propensity.^26,27^ FAAs that feature highly lipophilic and electron-withdrawing chalcogen–fluoroalkyl functional groups are becoming particularly relevant.^28^ Among these emergent motifs, the trifluoromethylthio group (−SCF_3_) is considered a privileged substituent due to one of the highest lipophilic parameters and a favorable physicochemical profile.^29,30^ Despite these promising parameters, SCF_3_-functionalization of peptide side chains and its effect on peptide properties has been largely underexplored so far due to a lack of available methodologies enabling its insertion. Recently, we reported the synthesis of Fmoc-protected trifluoromethylthiolated tryptophan Trp­(2-SCF_3_) and tyrosine Tyr­(3-SCF_3_) building blocks on a gram scale (Scheme 1A).^31^ These amino acids were efficiently incorporated into peptides, and we demonstrated their ability to remarkably enhance the local hydrophobicity of peptides and tune the H-bond donating ability of the Tyr­(3-SCF_3_) residue. Trifluoromethionine (TFM), an aliphatic SCF_3_-containing AA, has also been shown to markedly enhance local peptide hydrophobicity compared to canonical methionine or other aliphatic AAs.^32^ Most recently, Li and co-workers reported the late-stage Trp-selective electrophilic trifluoromethylthiolation of a series of native and complex therapeutic peptides (Scheme 1B).^33^ In the case of melittin, the corresponding SCF_3_-functionalized analog exhibited significantly increased human serum stability while retaining unattenuated antitumor activity.^33^ Moreover, a combination of sulfur and fluorine has been shown to drastically enhance cell uptake of cell-penetrating peptides, a property that is crucial for peptide–drug conjugate delivery systems.^34^ Further efforts to investigate the effects of chalcogen-associated fluoro functional groups on bioactivity and pharmacokinetic properties are required to understand their full potential for improving the ’drug-likeness’ of peptides.

**Scheme 1:**
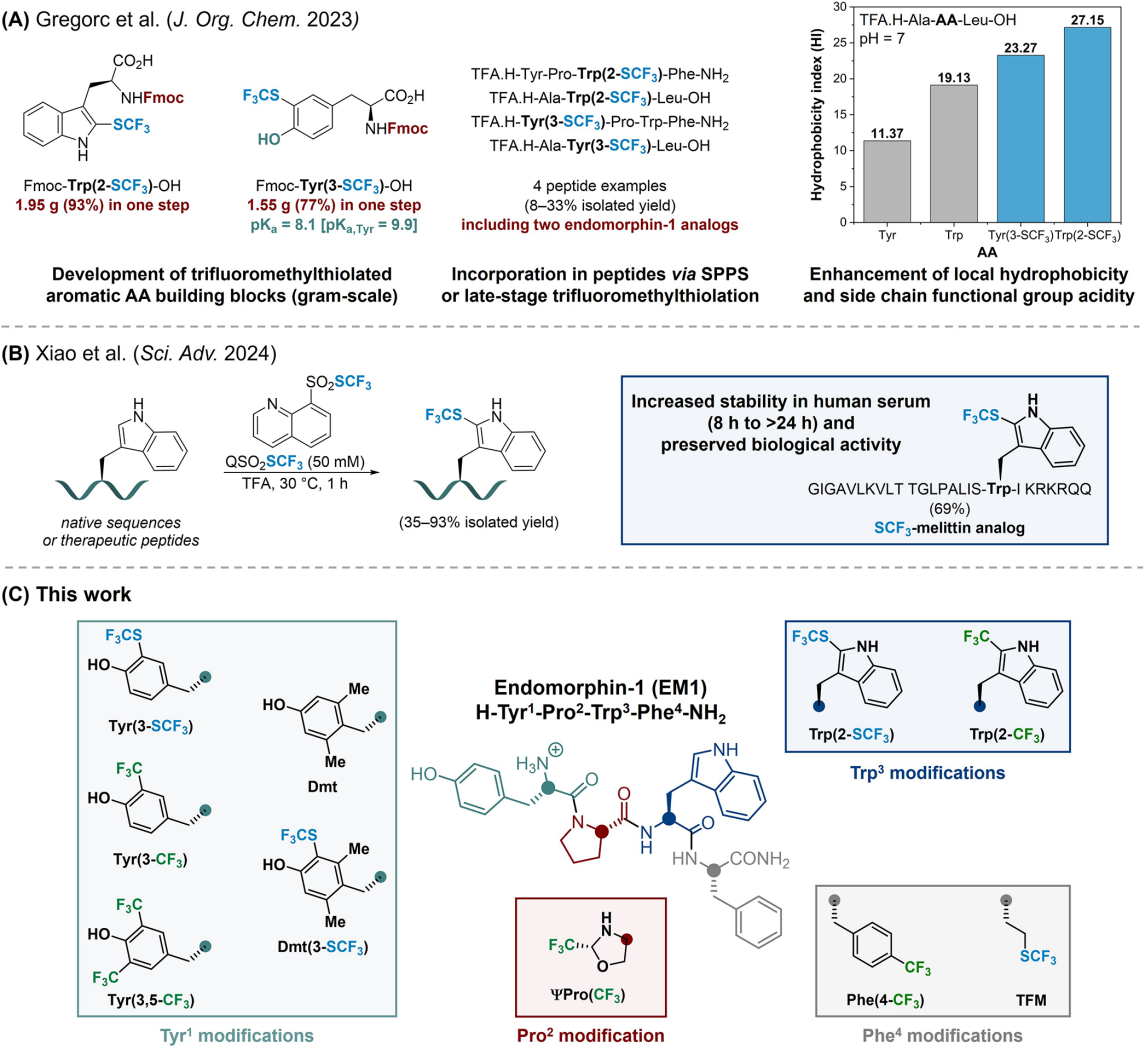
Overview of Trifluoromethylthiolation and Trifluoromethylation Strategies in Peptides and Their Properties^a^

a (A) Synthesis of Trp­(2-SCF_3_) and Tyr­(3-SCF_3_) building blocks, their incorporation into peptides, and resulting enhancement of local hydrophobicity in peptides.^31^ (B) Late-stage C2-selective Trp trifluoromethylthiolation of therapeutic peptides affording enhanced serum stability with preserved bioactivity.^33^ (C) Incorporation of SCF_3_- and CF_3_-amino acids into EM1 sequence for bioactivity and pharmacokinetic screening.

Herein, we systematically evaluated the impact of trifluoromethylthiolated and trifluoromethylated amino acids (Scheme 1C) on the physicochemical properties of short peptides and their utility as modified neuropeptide pharmacophores. The endogenous endomorphin-1 sequence (H-Tyr^1^-Pro^2^-Trp^3^-Phe^4^-NH_2_) was chosen as a case study to showcase the favorable effects of selective SCF_3_ and CF_3_ group incorporation into these biologically active targets with inherently poor pharmacokinetic properties. We first report herein the synthetic routes toward *N*-protected SCF_3_- and CF_3_-containing building blocks. Preassembled synthons were then incorporated in the EM1 peptide targeting all four amino acids *via* standard Fmoc-based solid-phase peptide synthesis (SPPS), affording 10 fluorinated ligands that were subjected to competitive μOR binding and functional activity assays. Additionally, relative ligand hydrophobicity and metabolic stability were assessed *via* RP-HPLC retention time analysis and human plasma incubation with UPLC-MS monitoring, respectively.

## Results and Discussion

### Fluorinated Building Blocks Synthesis

We previously reported the synthesis of trifluoromethylthiolated peptides *via* SPPS incorporation of Trp­(2-SCF_3_) and Tyr­(3-SCF_3_) building blocks, prepared by direct trifluoromethylthiolation of *N*-protected substrates using the *N*–SCF_3_ electrophilic reagent **1** under either Brønsted or Lewis acid activation (Scheme 2A).^31^ In this way, ready-to-use SPPS building blocks **3a** and **5a** were obtained in a single-step from commercial compounds, on a large scale (>2 g) and in excellent isolated yields (85–94%; Scheme 2A).

**Scheme 2:**
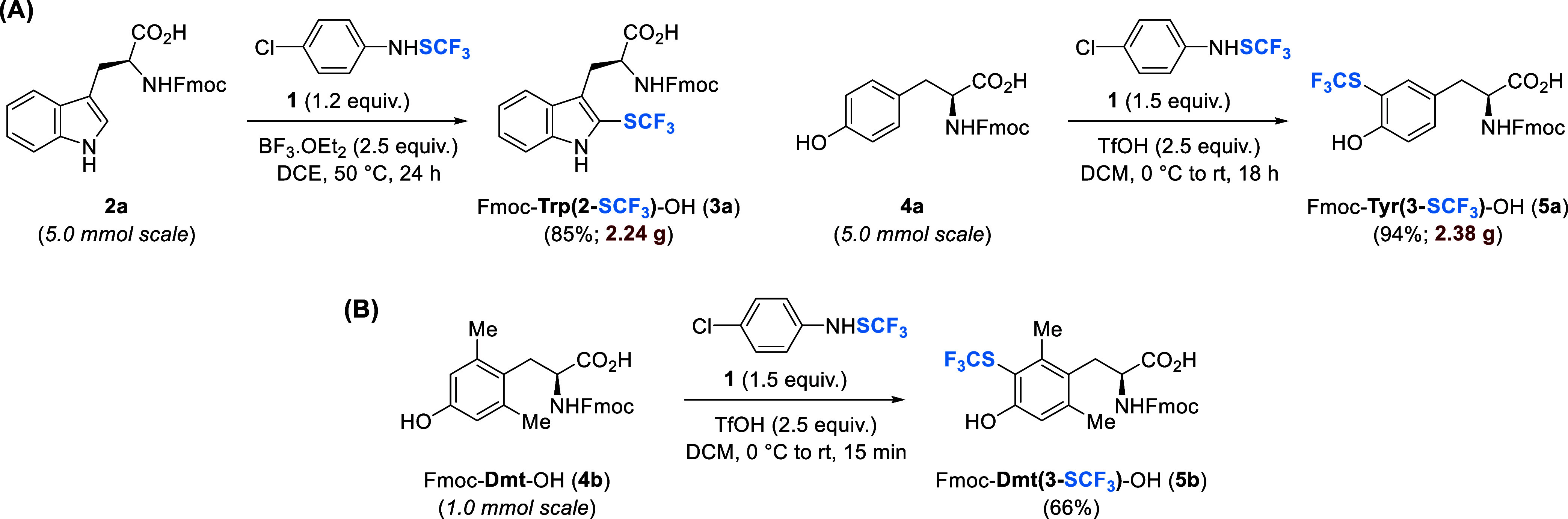
(A) Multigram-Scale Synthesis of *N*-Fmoc-Protected Trp­(2-SCF_3_) **3a** and Tyr­(3-SCF_3_) **5a** using our Previously Developed Method;^31^ (B) Direct Trifluoromethylthiolation of 2,6-Dimethyltyrosine (Dmt) **4b**

This methodology was extended to 2,6-dimethyltyrosine (Dmt), a widely used *N*-terminal Tyr^1^ modification in synthetic opioid ligands known to impart increased OR affinity and enhanced antinociceptive effects (Scheme 2B).^13,35–37^ Our motivation for synthesizing Dmt­(3-SCF_3_) was to combine the pharmacodynamic advantages of Dmt with the pharmacokinetically favorable SCF_3_ substitution. The additional electron-donating methyl groups in Dmt rendered it more reactive toward electrophilic trifluoromethylthiolation, as compared to the canonical amino acid **4a**, which required 18 h reaction times. Therefore, trifluoromethylthiolation of *N*-Fmoc-protected Dmt **4b** was quenched after 15 min to ensure monofunctionalization, affording analog **5b** in 66% yield.

To investigate the impact of the SCF_3_ group on opioid peptide properties relative to other fluorinated substituents, we also prepared trifluoromethylated analogs of tryptophan and tyrosine. Late-stage trifluoromethylation methodologies targeting Trp^38–45^ and Tyr^39,46^ residues in complex peptides have already been explored.^47^ Moreover, C2-trifluoromethylated Trp analogs are well represented as scope examples in numerous trifluoromethylation methodology reports.^48–61^ Despite this, robust gram-scale preparation of Fmoc-protected CF_3_-Trp/Tyr building blocks, along with their subsequent incorporation in peptides *via* SPPS, remains largely undocumented. To our knowledge, only two reports have recently described the synthesis of *N*-Fmoc-protected Tyr­(3-CF_3_) and Tyr­(3,5-CF_3_) analogs, but in low yield.^62,63^

First, we attempted to extend the radical copper-catalyzed trifluoromethylation conditions reported by Guerrero and Correa to the *N*-Fmoc-protected Trp series **2** (Conditions I in Scheme 3A).^38^ Applying the literature conditions to *C*-unprotected Trp **2a** resulted in 29% conversion to the desired CF_3_-analog **6a**. The final isolated yield of 13% is comparable to that of the literature yield for the synthesis of Ac-Trp­(2-CF_3_)-OH (25%).^38^ Trifluoromethylation of the methyl ester Fmoc-Trp-OMe **2b** gave **6b** in 34% conversion, similar to that of **6a**, and a slightly increased isolated yield of 32%. Nevertheless, an additional saponification step would be required prior to the SPPS use of **6b**. To improve the direct trifluoromethylation toward **6a**, we employed photocatalyst-free conditions under purple LED irradiation, recently reported by Jin and co-workers, which has proven effective for C2-trifluoromethylation of Trp-containing dipeptides and other biomolecules.^48^ Under such conditions (Conditions II in Scheme 3A), we observed near-quantitative conversion, and **6a** was isolated with a significantly improved yield of 40%.

**Scheme 3:**
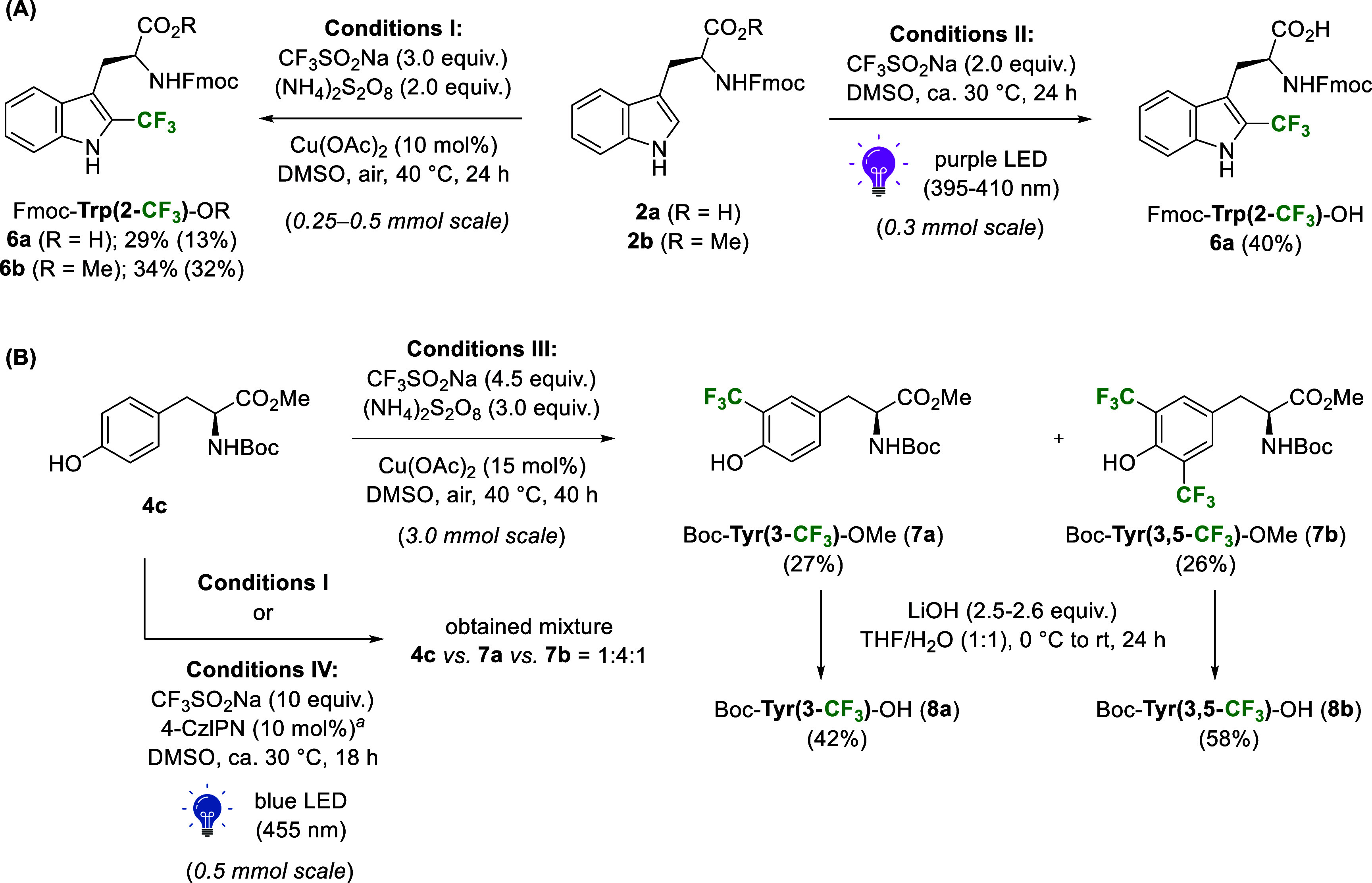
Synthesis of Trifluoromethylated Trp (A) and Tyr (B) Building Blocks by Adapting Reported Radical^38^ or Photoredox-Mediated^46,48^ Methods

a 4CzIPN = 1,2,3,5-tetrakis­(carbazol-9-yl)-4,6-dicyanobenzene.

The synthesis of Fmoc-protected Tyr­(3-CF_3_) analog following the literature Negishi cross-coupling conditions afforded only traces of the desired product in our hands.^62^ The low conversion rate and the need to use an expensive catalyst led us to turn to the trifluoromethylation of tyrosine derivatives under radical conditions (Scheme 3B). The Tyr^1^ residue, being the *N*-terminal AA in the parent EM1 sequence, allowed us to use *N*-Boc-protected substrate **4c**, instead of the Fmoc-protected substrate **4a** that features additional potential sites for C­(sp^2^)–CF_3_ bond formation. To our delight, optimized conditions used in the Trp series (Conditions I in Scheme 3A) immediately afforded a mixture of the monosubstituted Tyr­(3-CF_3_) **7a** and disubstituted Tyr­(3,5-CF_3_) **7b** products in good conversion (crude ratio: **4c** vs **7a** vs **7b** = 1:4:1). The *bis*-functionalized analog **7b** was deemed to represent a complementary and thus useful derivative for the following SAR study. Moreover, closely related column chromatography retention of **4c** and **7a** led us to employ larger reagent excess (Conditions III in Scheme 3B) to ensure complete conversion of **4c**, which enabled the isolation of **7a** and **7b** analogs in 27 and 26% yield, respectively (crude ratio: **7a** vs **7b** = ca. 3:2). After the saponification reaction, the ready-to-use building blocks **8a** and **8b** for SPPS were isolated in 42 and 58% yield, respectively. Additionally, photoredox-catalyzed trifluoromethylation of **4c** was performed under adapted literature conditions (*via* use of 4CzIPN photocatalyst in DMSO, Conditions IV in Scheme 3B).^46^ A crude mixture of **4c** vs **7a** vs **7b** = 1:4:1 was obtained, similar to the outcome of **4c** trifluoromethylation under radical Conditions I. Ultimately, the CF_3_SO_2_Na/(NH_4_)_2_S_2_O_8_ system proved better suited in the short term, as it could be easily performed at a larger scale of 3.0 mmol and produced the two useful fluorinated Tyr^1^ derivatives **8a** and **8b** for modifying EM1.

We then focused on incorporating fluorinated groups into the *C*-terminal Phe^4^–NH_2_ residue in EM1. Recent evidence indicated that the Phe^4^ position primarily drives conformational changes in the opioid receptor, rather than solely stabilizing the ligand’s bioactive conformation.^5,64^ Cryogenic electron microscopy structural studies of EM1-bound μOR complexes show that Phe^4^ inserts into an extended hydrophobic pocket formed by transmembrane (TM) helices, inducing a TM1 inward shift that promotes signaling bias and distinguishes EM1 from other μOR ligands.^64^ To modulate *C*-terminal hydrophobic interactions within EM1, we decided to incorporate Phe­(4-CF_3_) and trifluoromethionine (TFM) hydrophobic residues into our ligand series. Both synthons have been advantageously applied in recent work on peptide hydrogel development, resulting in materials with favorable mechanical and drug release properties.^27^ The Fmoc-protected Phe­(4-CF_3_) building block is commercially available and Fmoc-TFM **9** was synthesized from l-homocysteine in gram scale, following a recently reported protocol (Scheme 4A).^27^

**Scheme 4:**
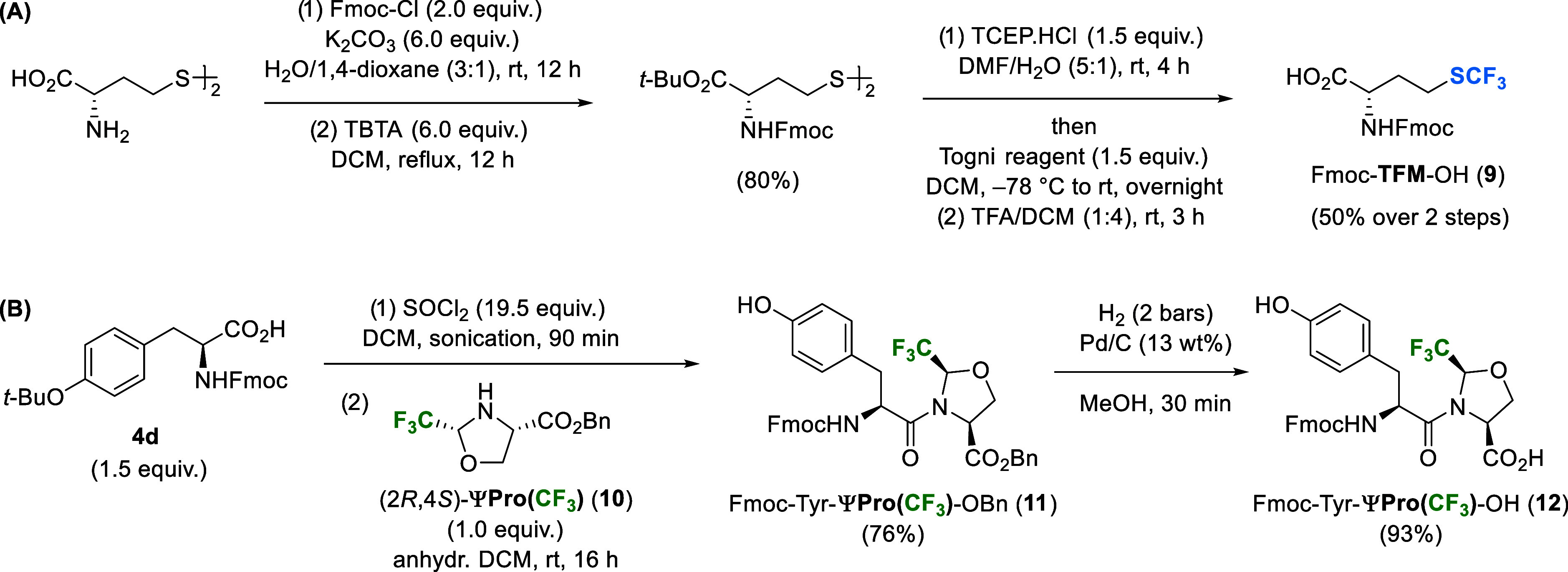
(A) Synthesis of *N*-Fmoc-Protected Trifluoromethionine Building Block **9**;^27^ (B) Synthesis of Tyr-ΨPro­(CF_3_) Dipeptide **12**

Lastly, the trifluoromethylated pseudoproline ΨPro­(CF_3_) was used to replace the Pro^2^ spacer for its capacity to modulate the *cis*/*trans* amide bond equilibrium of the Tyr^1^–Pro^2^ amide bond. The seminal NMR study on EM1 conformations in solution reported approximately 25% *cis* and 75% *trans* populations in both H_2_O and DMSO.^65^ Recent cryogenic electron microscopy structures of EM1−μOR complexes further support that the bioactive EM1 conformation adopts the extended *trans* isomer.^5,64^ In previous work, we demonstrated that the ΨPro­(CF_3_) residue lowers the *cis–trans* isomerization energy barrier Δ*G*_c–t_^‡^ by 3–5 kcal mol^–1^,^66^ controls the peptide backbone, and increases the local hydrophobicity.^25,67–69^ Its incorporation into the Pro^2^ position therefore provides a conformational hinge that could control the bioactive EM1 conformation and reduce the energy penalty for OR binding.

We previously reported the gram-scale synthesis of (2*R*,4*S*)-ΨPro­(CF_3_) **10** *via* a single-step Dean–Stark condensation of serine esters with trifluoroacetaldehyde ethyl hemiacetal.^67^ Compared with other fluorinated AAs, the strong electron-withdrawing effect of the trifluoromethyl group significantly decreases the nucleophilicity of the α-amino group. Therefore, preparation of Fmoc-X_AA_-ΨPro­(CF_3_)–OH dipeptide building blocks *via* in-solution peptide coupling of **10** with preformed X_AA_ acyl chlorides is necessary for effective incorporation of ΨPro­(CF_3_) residues by SPPS.^69,70^ The base-free peptide coupling of Fmoc-Tyr-Cl with (2*R*,4*S*)-ΨPro­(CF_3_)-OBn **10** gave the protected Tyr-ΨPro­(CF_3_) dipeptide **11** in a yield of 76% (Scheme 4B). Notably, the thionyl chloride activation of **4d** occurred with the concomitant removal of the *tert*-butyl protecting group of Tyr. Finally, the removal of the benzyl protecting group *via* Pd-catalyzed hydrogenolysis afforded the SPPS-compatible dipeptide **12** in near-quantitative yield (93%). The c*is*/*trans* conformer ratio of **12** in CDCl_3_ was determined by ^1^H–^1^H ROESY to be ca. 50:50.

### Solid-Phase Synthesis of Fluorinated EM1-Based Ligands

The series of ligands **L1–10** featuring fluorinated EM1 analogs was synthesized using SPPS (see ESI for SPPS procedures). EM1 and Dmt^1^ analog **L0** were prepared to serve as references for the following *in vitro* assays or physicochemical studies. All peptides were isolated in moderate to good yield (10–44%) and excellent purity (>96%) (Table 1), and characterized using HRMS and UPLC-MS (see ESI Figures S1–S12). Conformational study by NMR in deuterated methanol showed that ligands **L1–10** exhibited major *trans* prolyl amide bond conformation, except **L5** that features the ΨPro­(CF_3_) residue (*trans vs cis* = 29:71).

**Table 1:**
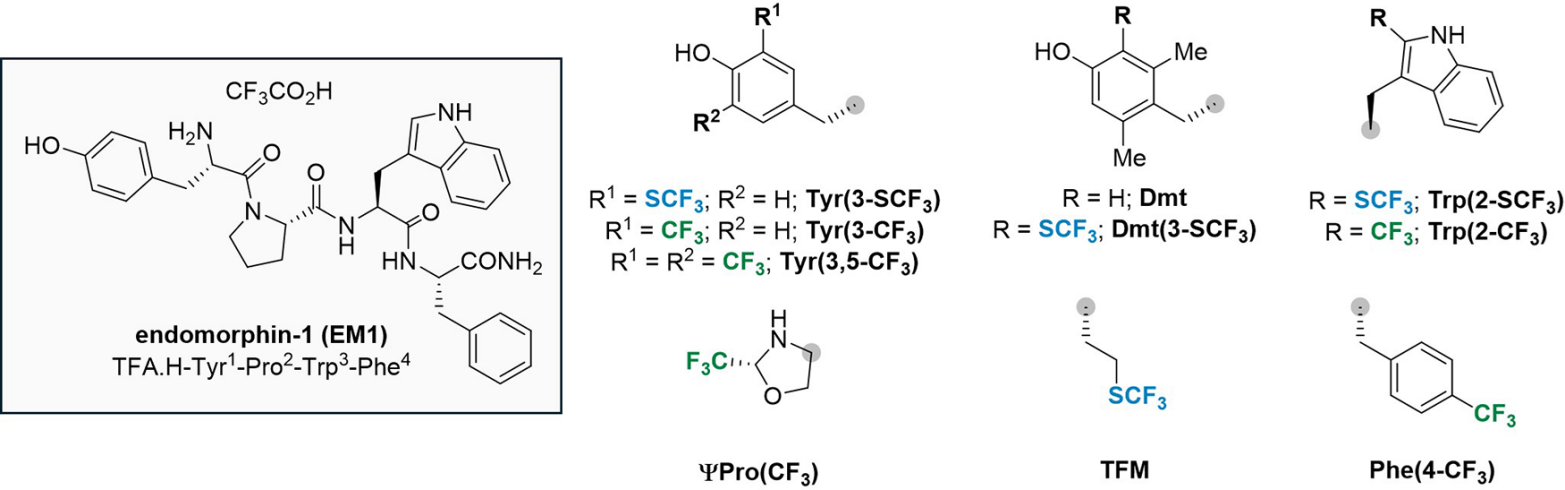
Synthesized Ligands **L0–10** and Parent Peptide **EM1** with Corresponding RP-HPLC Retention Times^a^

compound code	sequence	yield (%)	purity (%)^b^	*t*_R_ (min)^b^
**EM1**	TFA.H-Tyr-Pro-Trp-Phe-NH_2_	24	>99	8.06
**L0**	TFA.H-**Dmt**-Pro-Trp-Phe-NH_2_	24	>99	8.72
**L1**	TFA.H-**Tyr(3-SCF**_**3**_ **)**-Pro-Trp-Phe-NH_2_	19	>99	11.68
**L2**	TFA.H-**Tyr(3-CF**_**3**_ **)**-Pro-Trp-Phe-NH_2_	13	>99	10.94
**L3**	TFA.H-**Tyr(3,5-CF**_**3**_ **)**-Pro-Trp-Phe-NH_2_	22	97	12.25
**L4**	TFA.H-**Dmt(3-SCF**_**3**_ **)**-Pro-Trp-Phe-NH_2_	43	98	12.43, 12.87^c^
**L5**	TFA.H-Tyr-**ΨPro(CF**_**3**_ **)**-Trp–Phe-NH_2_	12	97	8.48
**L6**	TFA.H-Tyr-Pro-**Trp(2-SCF**_**3**_ **)**-Phe-NH_2_	26	>99	11.07
**L7**	TFA.H-Tyr-Pro-**Trp(2-CF**_**3**_ **)**-Phe-NH_2_	23	>99	9.62
**L8**	TFA.H-Tyr-Pro-Trp-**TFM**-NH_2_	10	>99	9.00
**L9**	TFA.H-Tyr-Pro-Trp-**Phe(4-CF**_**3**_ **)**-NH_2_	44	97	11.13
**L10**	TFA.H-**Dmt**-Pro-Trp-**Phe(4-CF**_**3**_ **)**-NH_2_	35	96	11.90, 12.13^c^

a Chemical modifications with respect to the canonical sequence are indicated in bold and illustrated above.

b RP-HPLC analysis (20 → 60% MeCN + 0.1% TFA in MQ H_2_O + 0.1% TFA, λ = 210 nm, 20 min run).

c Two HPLC signals are attributed to rotamers (see ESI for details).

The retention times (*t*_R_) of the RP-HPLC analysis were collected to give an approximation of ligand hydrophobicity, as compared to that of the canonical peptide EM1 (Table 1).^31^ The most drastic *t*_R_ increase was observed in the case of SCF_3_-modified Tyr^1^ analogs **L1** and **L4**, as well as ligands containing either two trifluoromethyl groups (**L3**) or a combination of two modifications (**L10**). In a previous investigation on the effects of SCF_3_-substitution on peptide hydrophobicity, we found that the introduction of the SCF_3_ group into the Tyr residue leads to higher relative increase in hydrophobicity compared to that observed with the Trp residue (see Scheme 1A).^31^ Herein, we observe a similar trend for EM1 (**L1** vs **L6**). The hydrophobic contribution of the trifluoromethyl group is indicated to be substantially lower than that of the trifluoromethylthio-substitution (**L1** vs **L2** and **L6** vs **L7**). Moreover, the two methyl substituents of **L0** appear to only moderately affect the RP-HPLC retention time (*t*_R_ = 8.72 min vs 8.06 min of EM1). However, the cumulative effect along with 3-SCF_3_ substitution of Dmt resulted in the most hydrophobic ligand (**L4**; *t*_R_ = 12.43 and 12.87 min). Unexpectedly, the chromatograms of the Dmt^1^-modified peptides **L4** and **L10** displayed two major HPLC signals, while in the case of peptide **L0**, a single peak was observed (see the ESI for chromatograms). We hypothesized that the two signals must correspond to rotamers due to the increased rotational barrier between the conformationally constrained Dmt^1^ and Pro^2^ residues.

### *In Vitro* Binding and Functional Activity at μOR

The binding affinity of fluorinated ligands **L1–10** was determined using a competitive radioligand binding assay on HEK293 cells expressing μOR and [^125^I]-DAMGO as the radioligand (Table 2, see the ESI for details). The μOR-selective peptide DAMGO was used as a reference ligand, alongside EM1, in binding and functional activity assays.

**Table 2:** μ-Opioid Receptor Binding Affinities and Functional Activity of DAMGO, **EM1**, and Fluorinated Ligands **L1–10**

ligand code	modification	*K*_*i*_ (nM)^a^	EC_50_ (nM)^b^	*E*_max_ (%)^b^
DAMGO		1.90 ± 1.71	30.4 ± 16.1	100
**EM1**		0.64 ± 0.35	3.70 ± 1.96	109 ± 15
**L1**	Tyr^1^(3-SCF_3_)	2.12 ± 1.14	5.65 ± 3.05	94 ± 19
**L2**	Tyr^1^(3-CF_3_)	2.90 ± 2.59	10.2 ± 5.21	80 ± 10
**L3**	Tyr^1^(3,5-CF_3_)	4.71 ± 2.63	19.8 ± 16.1	92 ± 9
**L4**	**Dmt** ^**1**^ **(3-SCF**_**3**_ **)**	**1.38 ± 1.31**	**0.93 ± 0.59**	**91 ± 12**
**L5**	ΨPro^2^(CF_3_)	7.05 ± 6.01	60.0 ± 31.7	80 ± 11
**L6**	Trp^3^(2-SCF_3_)	7.30 ± 4.21	8.00 ± 5.66	81 ± 13
**L7**	Trp^3^(2-CF_3_)	11.0 ± 7.94	8.26 ± 2.61	87 ± 7
**L8**	TFM^4^	8.50 ± 6.68	129 ± 153	61 ± 23
**L9**	Phe^4^(4-CF_3_)	24.9 ± 15.1	18.3 ± 17.0	55 ± 15
**L10**	**Dmt** ^**1**^ **/Phe** ^**4**^ **(4-CF**_**3**_ **)**	**1.51 ± 1.47**	**0.87 ± 0.37**	**67 ± 5**

a Determined in competitive radioligand binding assays (displacement of [^125^I]-DAMGO), *n* = 4.

b Effect of EM1 and **L1–10** on forskolin-stimulated cAMP accumulation by μOR, *n* = 3.

Most ligands exhibited μOR binding affinities within the nanomolar range. However, none of the analogs in the series demonstrated a superior inhibitory constant (*K*_*i*_) for μOR than canonical EM1. The most promising ligands of the series involved the Tyr^1^ residue variants (**L1–4** and **L10**), with Tyr^1^ representing the most essential pharmacophoric group for opioid activity.^71^ Notably, the SCF_3_ substitution of Tyr^1^ in **L1** demonstrated only 3.3-fold reduction in μOR binding vs EM1, while the Dmt^1^ variant **L4** exhibited superior affinity vs DAMGO and only a 2.2-fold reduction relative to EM1. The Dmt^1^ modification is known to significantly improve the overall opioid receptor binding affinity (lit. value for **L0**:^72^ *K*_*i*_ = 0.054 ± 0.01 nM at μOR), which is further evidenced by the 17-fold increase in binding affinity going from ligand **L9** to **L10**. Therefore, the additional trifluoromethylthio-substitution in **L4** disfavors μOR binding, as compared to the unmodified Dmt^1^ residue, but a comparable and thus high affinity to endogenous EM1 is retained. The incorporation of the ΨPro­(CF_3_) spacer in **L5** led to reduced μOR binding affinity (*K*_*i*_ = 7 nM), suggesting that the stereoelectronic effects of the CF_3_ group at Pro^2^ may disrupt the ligand–receptor interaction, next to a potentially disfavorable steric clash being introduced *via* the fluorinated group. Furthermore, Trp^3^-modified ligands **L6** and **L7** displayed an order of magnitude weaker μOR binding relative to that of EM1 as well. In addition, the Phe^4^-modified ligands **L8** and **L9** showed relatively weak μOR binding, as compared to the rest of the series (*K*_*i*_ = 9–25 nM). Despite being one order of magnitude lower compared to Tyr^1^-modified peptides **L1–4**, the binding affinity value of **L8** remains in the range of the other fluorinated peptide analogs, demonstrating that the incorporation of the nonaromatic TFM residue can be accommodated at the Phe^4^ position.

The functional activity of ligands **L1–10** was evaluated by measuring forskolin-stimulated cAMP accumulation in HEK293 cells expressing the μOR (Table 2). The potency (EC_50_) and efficacy (*E*_max_) were determined relative to those of the reference peptide DAMGO and compared to EM1 data. Most ligands except **L5** and **L8** exhibited higher μOR potency than DAMGO, with ligands containing the Dmt^1^ modification (**L4** and **L10**) displaying a net superior potency to that of EM1 (EC_50_ = 0.9 nM). The *E*_max_ parameter indicates the maximum response elicited by the ligand. Based on the obtained *E*_max_ values, ligands **L1–7** could be classified as full agonists and **L8–10** as partial agonists. Further investigation in the ligands’ **L1–10** mode of action is warranted; however, the presented data suggest a similar profile to parent EM1 when considering cAMP signaling only.

### *In Vitro* Human Plasma Stability

The introduction of unnatural fluorinated amino acids can interfere with proteolytic digestion processes and consequently increase the enzymatic stability of peptides.^22,23^ We investigated the human plasma stability of two of the most promising ligands (**L4** and **L10**) with regard to μOR binding and potency data. Half-lives of natural EM1 and nonfluorinated **L0** were also determined to quantify the sole impact of SCF_3_ and CF_3_ substitution on peptide stability.

In agreement with the literature, endogenous EM1 exhibited a poor half-life (*t*_1/2_) of ca. 6 min (Table 3, see ESI for experimental details).^73^ The introduction of 2,6-dimethyltyrosine in **L0** already demonstrated 5-fold increase in *t*_1/2_ and a combination of Dmt and Phe­(4-CF_3_) residues (**L10**) additionally improved the stability 4-fold relative to **L0** (20-fold vs EM1). The most stable ligand proved to be **L4** with drastically improved *t*_1/2_ of ca. 7.5 h (72-fold and 14-fold increase vs EM1 and **L0**, respectively). In all cases, the detected metabolites indicated the initial cleavage of the Pro–Trp amide bond. Hence, the additional trifluoromethylthio-substitution of Dmt^1^ is considerably more effective in improving the metabolic stability compared to trifluoromethylation of the *C*-terminal residue in **L10** (Δ*t*_1/2_ = ca. 5.5 h). The obtained data further reinforce the significance of site-specific SCF_3_ incorporation into bioactive peptide residues for improving their drug-like properties, including hydrophobicity and metabolic stability.

**Table 3:** Human Plasma Stability of Selected Ligands **L4** and **L10** vs **EM1** and **L0** as Reference Peptides

ligand code	sequence	*t*_1/2_ (min)^a^
**EM1**	TFA.H_2_N-Tyr-Pro-Trp-Phe-NH_2_	6.4 ± 0.2
**L0**	TFA.H_2_N-**Dmt**-Pro-Trp-Phe-NH_2_	32.3 ± 0.7
**L4**	TFA.H_2_N-**Dmt(3-SCF**_**3**_ **)**-Pro-Trp-Phe-NH_2_	**463 ± 9**
**L10**	TFA.H_2_N-**Dmt**-Pro-Trp-**Phe(4-CF**_**3**_ **)**-NH_2_	130 ± 8

a The data are presented as the mean ± SEM (*n* = 3).

## Conclusion

Herein, we report the synthesis and *in vitro* evaluation of a series of endomorphin-1-based neuropeptides featuring SCF_3_- and CF_3_-functionalization of all four residues. To this end, 8 ready-to-use SCF_3_- or CF_3_-building blocks for SPPS have been efficiently prepared with satisfactory yields. Together with the commercially available Phe­(4-CF_3_), they were incorporated *via* SPPS to afford a series of 10 fluorinated EM1 analogs in moderate to high isolated yields (10–44%) and excellent purities (>96%). Ligands **L1–10** were subjected to *in vitro* μOR binding and functional activity assays. The obtained data suggest that trifluoromethylthio-substitution (as well as trifluoromethyl-substitution to a somewhat lesser extent) across all four EM1 residues produced equipotent ligands to the canonical structure (up to *K*_*i*_ of 1.4 nM and up to EC_50_ of 0.9 nM) with enhanced hydrophobicity, evidenced by significantly increased RP-HPLC retention. Peptides **L1–L4** and **L10**, featuring modifications of the *N*-terminal Tyr^1^ pharmacophoric group, were revealed to be the most promising. Ligand **L4** has demonstrated the potential of Dmt^1^(3-SCF_3_) residue in opioid receptor agonist design for its ability to remarkably increase the overall peptide stability in human plasma (72-fold vs EM1 and 14-fold vs nonfluorinated EM1­(Dmt^1^) **L0**), while displaying equal μOR binding affinity and improved μOR potency. This study further elucidates the potential of trifluoromethylthiolated residues, including Tyr­(3-SCF_3_) and Dmt­(3-SCF_3_), to enhance the pharmacokinetic properties of the bioactive peptides. The developed trifluoromethylthiolated residues should be considered part of the peptide medicinal chemist’s toolbox, and their future application in opioid peptide design, as well as in broader therapeutic peptide development, is anticipated.

## Supplementary Material



## Data Availability

The data underlying this study are available in the published article and its Supporting Information.

## Notes

Experimental procedures, characterization data, RP-HPLC chromatograms of opioid ligands **EM1** and **L0**–**L10**, biological activity assays, plasma stability assays, copies of ^1^H, ^13^C, and ^19^F NMR spectra for all new compounds (PDF)
