# Supplementary material for: Trifluoromethylthio and Trifluoromethyl Functionalization of Endomorphin‑1 Enhances its Hydrophobicity and Plasma Stability while Preserving its Affinity for the μ‑Opioid Receptor
Source: J Org Chem. 2026 Apr 15;91(16):5598–607. doi: 10.1021/acs.joc.6c00321 (PMC13122644; doi:10.1021/acs.joc.6c00321)
Supplement: Supplementary file 1 [file jo6c00321_si_001.pdf]

Supporting information

Trifluoromethylthio and Trifluoromethyl Functionalization of Endomorphin-1 Enhances its Hydrophobicity and Plasma Stability while Preserving its Affinity for the μ-Opioid Receptor

Jure Gregorc,[a,b,c,‖] Jolien De Neve,[d,‖] Karine Guitot,[b,c] Brian J. Holleran,[e] Nathalie Lensen,[b,c] Louis Gendron,[e] Thierry Brigaud,[b,c,*] Jernej Iskra,[a,*] Steven Ballet,[d,*] Grégory Chaume[b,c,*]

[a]University of Ljubljana, Faculty of Chemistry and Chemical Technology, Večna pot 113, 1000 Ljubljana, Slovenia.

[b]CY Cergy Paris Université, CNRS, BioCIS, 95000 Cergy Pontoise, France.

[c]Université Paris-Saclay, CNRS, BioCIS, 91400 Orsay, France.

[d]Research Group of Organic Chemistry, Departments of Chemistry and Bioengineering Sciences, Vrije Universiteit Brussel, B-1050 Brussels, Belgium.

[e]Institut de Pharmacologie de Sherbrooke, Department of Pharmacology and Physiology, Faculty of Medicine and Health Sciences, Université de Sherbrooke, 3001, 12e Avenue Nord, J1H 5N4 Sherbrooke, Quebec, Canada.

Corresponding authors email:

Thierry.Brigaud@cyu.fr

Jernej.Iskra@fkkt.uni-lj.si

Steven.Ballet@vub.be

Gregory.Chaume@cyu.fr

Table of Contents

1. Experimental section S3
2. Preparation of fluorinated building blocks S5
   Starting substrates and reagents S5
   Fluorinated building block synthesis S7
3. Solid-phase peptide synthesis of opioid ligands S17
   General SPPS procedure S17
   Opioid ligands S18
4. Chemical structure and RP-HPLC chromatograms of opioid ligands S32
5. Biological activity assays S38
   Materials S38
   Binding on MOP S38
   Inhibition of cAMP production S38
6. Plasma stability assay S40
7. Copies of NMR spectra S41
   Starting substrates and reagents S41
   Fluorinated building blocks S43
   Opioid ligands S59
8. References S74

1. Experimental section

All chemicals and solvents used were purchased from commercial sources and were used without prior purification (Sigma-Aldrich, Fluorochem, Carbosynth, Iris Biotech GmbH). Solvents used for reactions, extractions and column chromatography were of technical grade. Water for liquid chromatography (LC) analysis was purified using a Millipore MilliQ water purification system. DMF for peptide coupling and LC solvents were of HPLC grade. Organic extracts were dried over technical grade anhydrous Na_2SO_4 or $MgSO_4$. Room temperature (rt) refers to 20–25 °C. For reactions carried out at higher temperatures, the reaction vessels were placed in a heating block.

^{1}H, ^{13}C and ^{19}F NMR spectra were recorded using a Bruker Avance NEO 600 MHz spectrometer (operating at 600 MHz for ^{1}H, 151 MHz for ^{13}C and 565 MHz for ^{19}F at 296 K), Bruker Avance III 500 MHz spectrometer (operating at 500 MHz for ^{1}H, 126 MHz for ^{13}C and 471 MHz for ^{19}F at 296 K), or Bruker Avance Neo 400 MHz spectrometer (operating at 400 MHz for ^{1}H, 101 MHz for ^{13}C and 376 MHz for ^{19}F at 296 K). NMR experiments were processed and analyzed using the MestReNova or TopSpin software. ^{1}H and ^{13}C chemical shifts are reported as δ scale in ppm relative to the residual signals of $CDCl_3$ (δ = 7.26 ppm and 77.16 ppm, respectively), DMSO-d_6 (δ = 2.50 ppm and 39.52 ppm, respectively), and MeOD-d_3 (δ = 3.31 ppm and 49.00 ppm, respectively). Hexafluorobenzene (C_6F_6) was used as an external reference for ^{19}F NMR spectra.[1] Coupling constants (J) are given in hertz (Hz). Abbreviations for NMR data are s (singlet), bs (broad singlet), d (doublet), t (triplet), q (quartet), m (multiplet) and combinations thereof. NMR resonance assignments and conformational populations were confirmed using 2D correlation spectroscopy (COSY), 2D nuclear Overhauser effect spectroscopy (NOESY), 2D rotating-frame nuclear Overhauser effect spectroscopy (ROESY), 2D heteronuclear multiple-bond correlation spectroscopy (HMBC) and 2D heteronuclear single quantum coherence spectroscopy (HSQC). The solvent signal in ^{1}H and 2D spectra of EM1 analogues in MeOH-d_3 was suppressed using the Watergate pulse sequence.

High-resolution mass spectra (HRMS) were acquired using an Agilent 6224 Accurate Mass TOF LC/MS or an Agilent QTOF 6530 (source ESI, resolution 12000) or a Synapt XS system equipped with a Lockspray Exact Mass Ionization (source ESI) using Leu Enkephalin as a reference. Infrared spectra (IR) were recorded using a Perkin-Elmer Spectrum BX FTIR with frequencies given in cm^{-1}. Melting points were determined using a Kofler micro hot stage and are uncorrected. The specific angles of rotation of optically pure compounds were measured using an Anton Paar MCP 5100 polarimeter (sodium D-line – 589 nm, 20 °C) at concentrations given in g/100 mL of the indicated solvent.

Solid-phase peptide synthesis was carried out manually using the standard Fmoc-strategy protocols. Peptide syntheses were monitored using a reverse-phase (RP) ultra performance liquid chromatography coupled mass spectrometer (UPLC-MS). The analyses were performed either 1) on a Waters Acquity UPLC instrument equipped with a Luna Omega PS-C18 column (1.5 µm, 2.1 x 50 mm) and coupled to a single quadrupole ESI-MS (Micromass ZQ) under the following conditions: flow rate of 0.5 mL/min at room temperature; linear gradient of mobile phases 90% Milli-Q H_2O + 0.1% TFA/10% MeCN + 0.1% TFA → 90% MeCN + 0.1% TFA/10%

Milli-Q H_2O + 0.1% TFA; 3 min; detection at 210 nm and 254 nm or 2) on a Waters Acquity System UPLC instrument consisting of 4 parts: Binary Solvent Manager, Sample Manager, Column Manager and PDA Detector, and equipped with a AcquityTM Premier BEH C18, 130 Å column (1.7 µm, 2.1 x 100 mm) and coupled with a ESI-MS unit (Lockspray Exact Mass Ionization) under the following conditions: flow rate of 0.5 ml/min at room temperature, linear gradient of mobile phases 99% Milli-Q H_2O + 0.01% formic acid/1% MeCN + 0.01% formic acid → 99% MeCN + 0.01% formic acid/1% Milli-Q H_2O + 0.01% formic acid over 10 min after which the final gradient remained until 12 min. RP high-performance liquid chromatography (HPLC) purification of peptides was performed using either 1) an semi-preparative Agilent 1260 Infinity II HPLC system equipped with a DAD detector under the following conditions: Luna Omega PS-C18 column (5 µm, 100 Å, 250 x 10 mm); flow rate of 4.0 mL/min; injection volume 100–850 µL, linear gradient of mobile phases Milli-Q H_2O + 0.1% TFA (A) and MeCN + 0.1% TFA (B); detection at 215 nm and 250 nm or 2) on a preparative Gilson HPLC system with a Gilson 322 pump over a Dr. Maisch ReproSil 100 C18 column (5 µm, 100 Å, 250 x 20 mm) and a Gilson UV/Vis-156 detector at 215 nm. The solvent system consists of 0.1% TFA in Milli-Q water (A) and 0.1% TFA in MeCN (B) with a flow rate of 20 or 15 mL/min, respectively. The software used is UniPoint. RP-HPLC analysis for the determination of peptide purity and hydrophobicity index was performed on an Agilent 1200 analytical HPLC system, equipped with a DAD detector. The RP-HPLC conditions were as follows: Zorbax Eclipse XDB-C18 column (5 µm, 100 Å, 150 x 4.6 mm); flow rate of 1.0 mL/min; injection volume 10–25 µL; specified linear gradient of mobile phases MQ H_2O + 0.1% TFA, MeCN + 0.1% TFA; detection at 210 nm.

Column chromatography (CC) was performed on silica gel (silica gel 60, particle size: 0.035–0.070 mm). Reactions were monitored by thin-layer chromatography (TLC) (Merck, silica gel 60 F_{254}, 0.25 nm) using mixtures of ethyl acetate (EtOAc), *n*-hexane (*n*-hex), cyclohexane (cy-hex), dichloromethane (DCM) or methanol (MeOH). Visualization was achieved by UV lamp irradiation and/or staining with phosphomolybdic acid or ninhydrin, followed by heating.

Safety considerations:

Caution! *All experiments were conducted in accordance with the safety guidelines for handling hazardous chemicals. Trifluoromethanesulfonic acid (TfOH) and trifluoroacetic acid (TFA) are highly corrosive, strongly acidic reagents that can cause severe chemical burns upon contact with skin or eyes; Thionyl chloride ($SOCl_2$) is a highly reactive, corrosive reagent that releases toxic gasses (SO_2 and HCl) upon contact with moisture; Dichloromethane is a volatile, toxic, and a suspected carcinogenic solvent; N,N-Dimethylformamide (DMF) is toxic and readily absorbed through the skin, with potential reproductive and hepatic toxicity. All reactions involving these reagents should be conducted in a well-ventilated fume hood with the appropriate personal protective equipment.*

2. Preparation of fluorinated building blocks

Starting substrates and reagents

Compounds **2a** (Fluorochem Ltd.), **4a** (Fluorochem Ltd.), **4c'** (Fluorochem Ltd.), and **4d** (Iris Biotech GmbH) were bought from commercial sources and used without additional purification. 2,4,5,6-Tetra(9*H*-carbazol-9-yl)isophthalonitrile (4CzIPN) was synthesized following a literature procedure.[2]

Fmoc-L-Dmt-OH (**4b**),[3] Fmoc-L-TFM-OH (**9**),[4] and (2*R*,4*S*)-ΨPro(CF_3) (**10**)[5] were synthesized following procedures detailed in our previous publications.

***N*-(4-chlorophenyl)-*S*-(trifluoromethyl)thiohydroxylamine (1)**

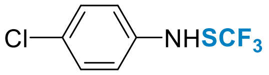

Synthesized following a literature procedure.[6,7] A vacuum-dried 250 mL round-bottom flask was charged with diisopropylethylamine (7 mL, 40 mmol, 1.0 equiv.) and DCM (80 mL). The mixture was cooled to –25 °C before the dropwise addition of diethylaminosulfur trifluoride (5.9 mL, 47 mmol, 1.2 equiv.) over 30 minutes, followed by the dropwise addition of trifluoromethyltrimethylsilane (6.5 mL, 44 mmol, 1.1 equiv.) over 20 minutes. After stirring for an additional hour at –20 °C, 4-chloroaniline (5.1 g, 40 mmol, 1.0 equiv.) was added at 0 °C in several portions over 5 hours. The reaction mixture was allowed to warm to room temperature and was stirred overnight. Following the reaction, the mixture was washed with distilled water. The aqueous phase was extracted with DCM (2 x 80 mL) and the combined organic layers were dried over anhydrous Na_2SO_4, filtered and concentrated under reduced pressure. The crude product was purified by silica gel flash CC (mobile phase: *n*-hex), affording *N*-(4-chlorophenyl)-*S*-(trifluoromethyl)thiohydroxylamine **1**. NMR data were in agreement with the literature.[6,7]

Yield: 6.73 g (29.6 mmol, 74%) of yellow-orange oil.

^{1}H NMR (500 MHz, $CDCl_3$): δ = 7.25–7.22 (m, 2H), 7.04–7.00 (m, 2H), 5.11 (s, 1H).

^{19}F NMR (471 MHz, $CDCl_3$): δ = –52.71 (s, 3F).

Fmoc-L-Trp-OMe (2b)

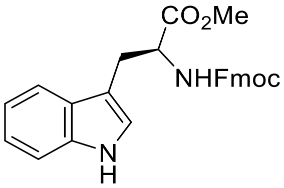

Synthesized following a literature procedure.[8] A round-bottom flask was charged with Fmoc-L-Trp-OH **2a** (2.14 g, 5.0 mmol, 1.0 equiv.) and suspended in MeOH (10 mL, 0.5 M). Trimethylsilyl chloride (1.28 mL, 10.0 mmol, 2.0 equiv.) was then added and the resulting

suspension was stirred at room temperature for 16 hours under ambient atmosphere (open flask). The mixture was diluted with H_2O (50 mL) and extracted with DCM (3 x 40 mL). The combined organic layers were washed sequentially with sat. $NaHCO_3$ (aq., 40 mL), H_2O (60 mL), and brine (60 mL). The organic phase was dried over anhydrous Na_2SO_4, filtered and concentrated *in vacuo*, affording pure **2b**.

Yield: 2.16 g (4.90 mmol, 98 %; mixture of rotamers: 88:12) of white solid.

^{1}H NMR (600 MHz, $CDCl_3$) of the major rotamer: δ = 8.08 (s, 1H), 7.77 (d, J = 7.6 Hz, 2H), 7.56 (dd, J = 13.3, 7.7 Hz, 3H), 7.43–7.38 (m, 2H), 7.37 (d, J = 8.2 Hz, 1H), 7.32–7.28 (m, 2H), 7.23–7.19 (m, 1H), 7.16–7.11 (m, 1H), 6.95 (s, 1H), 5.36 (d, J = 8.3 Hz, 1H), 4.75 (dd, J = 8.2, 5.2 Hz, 1H), 4.43 (dd, J = 10.3, 7.4 Hz, 1H), 4.37 (dd, J = 10.8, 6.9 Hz, 1H), 4.21 (t, J = 7.2 Hz, 1H), 3.70 (s, 3H), 3.34 (d, J = 5.4 Hz, 2H).

Boc-L-Tyr-OMe (4c)

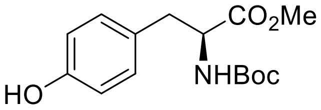

Synthesized following a literature procedure.[9] A round-bottom flask was charged with methyl L-tyrosinate (2.32 g, 10.0 mmol, 1.0 equiv.) and dissolved in MeOH (40 mL). $NaHCO_3$ (1.76 g, 21.0 mmol, 2.1 equiv.) was added and the suspension was stirred for 15 minutes at room temperature. A solution of di-*tert*-butyl dicarbonate (2.40 g, 11.0 mmol, 1.1 equiv.) in MeOH (10 mL) was slowly added and the reaction mixture was stirred for 4 hours at room temperature. Following the reaction, the volatile components were removed *in vacuo* and the resulting crude mixture was suspended in H_2O (75 mL). The pH was adjusted to 2–3 with 1 M HCl (aq.) and the aqueous layer was extracted with EtOAc (3 x 75 mL). The organic phase was dried over anhydrous Na_2SO_4, filtered and concentrated *in vacuo*. The crude product was purified by silica gel CC (dry loading; mobile phase: EtOAc/cy-hex 1:2), affording pure **4c**. NMR data were in agreement with the literature.[9]

Yield: 2.86 g (9.68 mmol, 97%) of white foamy solid.

^{1}H NMR (400 MHz, $CDCl_3$) of the major rotamer: δ = 6.97 (d, J = 8.5 Hz, 2H), 6.73 (d, J = 8.1 Hz, 2H), 5.44 (bs, 1H), 4.99 (d, J = 8.4 Hz, 1H), 4.60–4.47 (m, 1H), 3.71 (s, 3H), 3.03 (dd, J = 13.9, 5.8 Hz, 1H), 2.97 (dd, J = 14.0, 6.1 Hz, 1H), 1.42 (s, 9H).

Fluorinated building block synthesis

Fmoc-L-Trp(2-SCF$_3$)-OH (3a)

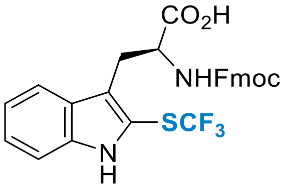

Synthesized following our literature procedure, scaled to 5.0 mmol.[7] A 100 mL round-bottom flask was charged with Fmoc-L-Trp-OH **2a** (2.13 g, 5.0 mmol, 1.0 equiv.), reagent **1** (1.30 g, 5.7 mmol, 1.1 equiv.), and DCE (25 mL, 0.25 M). The reaction mixture was cooled in an ice bath and stirred for 5 minutes before $BF_3{\cdot}OEt_2$ (48 wt% solution in diethyl ether, 3.6 mL, 13.6 mmol, 2.7 equiv.) was added dropwise. The ice bath was then removed and the mixture was heated at 50 °C for 24 hours. After cooling to room temperature, the reaction mixture was diluted with DCM (50 mL) and washed sequentially with 10% HCl (aq., 3 x 40 mL) and brine (1 x 60 mL). The organic phase was dried over anhydrous Na_2SO_4, filtered and concentrated *in vacuo*. The crude product was suspended in *n*-hex/DCM (ratio adjusted to dissolve residual reagent **1** and aniline), heated to reflux with vigorous stirring, and allowed to gradually cool to room temperature. The triturated solid was collected by filtration and dried in an oven at 40 °C overnight to afford **3a** as an off-white solid. NMR data were in agreement with the literature.[7]

Yield: 2.24 g (4.25 mmol, 85%) of off-white solid.

^{1}H NMR (600 MHz, DMSO-*d*$_6$) of the major rotamer: δ = 12.77 (bs, 1H), 11.89 (s, 1H), 7.87 (d, *J* = 7.6 Hz, 2H), 7.82 (d, *J* = 8.6 Hz, 1H), 7.76 (d, *J* = 8.1 Hz, 1H), 7.64 (dd, *J* = 13.5, 7.5 Hz, 2H), 7.44–7.37 (m, 3H), 7.30 (dd, *J* = 7.5, 1.1 Hz, 1H), 7.29–7.21 (m, 2H), 7.05 (ddd, *J* = 8.0, 6.9, 1.0 Hz, 1H), 4.27 (td, *J* = 8.7, 5.7 Hz, 1H), 4.21–4.10 (m, 3H), 3.45–3.37 (m, 1H), 3.22 (dd, *J* = 14.3, 8.9 Hz, 1H).

^{19}F NMR (565 MHz, DMSO-*d*$_6$): δ = –42.29 (s, 3F, SCF_3).

HRMS (ESI) m/z: $[M + H]^+$ Calcd. for $C_{27}H_{22}F_3N_2O_4S^+$ 527.1247; Found: 527.1247.

Fmoc-L-Trp(3-SCF$_3$)-OH (5a)

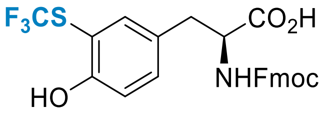

Synthesized following our literature procedure, scaled to 5.0 mmol.[7] A 100 mL round-bottom flask was charged with Fmoc-L-Tyr-OH **4a** (2.02 g, 5.0 mmol, 1.0 equiv.), reagent **1** (1.71 g, 7.5 mmol, 1.5 equiv.) and DCM (50 mL, 0.1 M). The reaction mixture was cooled in an ice bath and stirred for 5 minutes before TfOH (1.1 mL, 12.5 mmol, 2.5 equiv.) was slowly added. The ice bath was then removed, and the mixture was stirred at room temperature for 24 hours. The reaction mixture was diluted with DCM (20 mL) and washed sequentially with 10% HCl (aq., 3 x 40 mL) and brine (1 x 60 mL). The organic phase was dried over anhydrous Na_2SO_4, filtered, and concentrated *in vacuo*. The crude product was suspended in *n*-hex/DCM (ratio adjusted to dissolve residual reagent **1** and aniline), heated to reflux with vigorous stirring, and

allowed to gradually cool to room temperature. The triturated solid was collected by filtration and dried in an oven at 40 °C overnight to afford **5a** as an off-white solid. NMR data were in agreement with the literature.[7]

Yield: 2.38 g (4.71 mmol, 94%) of off-white solid.

^{1}H NMR (600 MHz, DMSO-*d6*) of the major rotamer: *δ* = 12.76 (bs, 1H), 10.36 (s, 1H), 7.88 (d, *J* = 7.6 Hz, 2H), 7.73 (d, *J* = 8.5 Hz, 1H), 7.67–7.60 (m, 2H), 7.46 (d, *J* = 2.2 Hz, 1H), 7.44–7.38 (m, 2H), 7.33–7.25 (m, 3H), 6.94 (d, *J* = 8.4 Hz, 1H), 4.22–4.14 (m, 3H), 4.11 (ddd, *J* = 10.6, 8.5, 4.4 Hz, 1H), 3.01 (dd, *J* = 14.0, 4.3 Hz, 1H), 2.79 (dd, *J* = 13.9, 10.6 Hz, 1H).

^{19}F NMR (565 MHz, DMSO-*d6*): *δ* = –41.59 (s, 3F, SCF_3).

HRMS (ESI) m/z: $[M + H]^+$ Calcd. for $C_{25}H_{21}F_3NO_5S^+$ 504.1087; Found: 504.1087.

Fmoc-L-Dmt(3-SCF$_3$)-OH (5b)

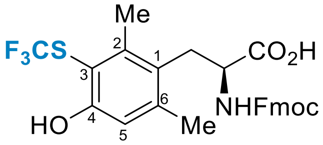

A round-bottom flask was charged with Fmoc-L-Dmt-OH **4b** (431 mg, 1.00 mmol, 1.0 equiv.), reagent **1** (341 mg, 1.50 mmol, 1.5 equiv.), and DCM (10 mL, 0.1 M). The reaction mixture was cooled in an ice bath and stirred before TfOH (0.22 mL, 2.5 mmol, 2.5 equiv.) was added dropwise. The ice bath was then removed and the mixture was stirred for 15 minutes. The reaction mixture was diluted with DCM (40 mL) and washed sequentially with 1 M HCl (aq., 3 x 50 mL) and brine (1 x 50 mL). The organic phase was dried over anhydrous $MgSO_4$, filtered, and concentrated *in vacuo*. The crude product was purified by automated silica gel flash CC (mobile phase: DCM/MeOH 10:1 → 5:1), affording pure **5b**.

Yield: 351 mg (0.66 mmol, 66%) of brown solid.

mp 180–184 °C

$[\alpha]_D^{20}$ = –14.11 (*c* 0.17, MeOH)

^{1}H NMR (600 MHz, MeOD-*d4*) of the major rotamer: *δ* = 7.78 (d, *J* = 7.6 Hz, 2H, Fmoc arom.), 7.61 (dd, *J* = 7.6, 3.5 Hz, 2H, Fmoc arom.), 7.38 (dd, *J* = 7.5, 3.8 Hz, 2H, Fmoc arom.), 7.33–7.25 (m, 2H, Fmoc arom.), 6.67 (s, 1H, C5-H Dmt), 4.39 (dd, *J* = 9.2, 6.0 Hz, 1H, Cα-H Dmt), 4.26 (dd, *J* = 10.6, 7.0 Hz, 1H, Fmoc CH_2-Hb), 4.20 (dd, *J* = 10.5, 7.0 Hz, 1H, Fmoc CH_2-Ha), 4.12 (t, *J* = 7.0 Hz, 1H, Fmoc CH), 3.23 (dd, *J* = 14.6, 6.0 Hz, 1H, Cβ-Hb Dmt), 3.04 (dd, *J* = 14.6, 9.3 Hz, 1H, Cβ-Ha Dmt), 2.59 (s, 3H, C2-CH_3 Dmt), 2.34 (s, 3H, C6-CH_3 Dmt).

^{13}C{^{1}H} NMR (101 MHz, MeOD-*d4*): *δ* = 175.3 (C=O, CO_2H), 160.0 (C, C4 Dmt), 158.4 (C=O, Fmoc), 145.7 (C, C2 Dmt), 145.3 (C, Fmoc arom.), 145.2 (C, Fmoc arom.), 144.4 (C, C6 Dmt), 142.6 (C, Fmoc arom.), 142.5 (C, Fmoc arom.), 131.4 (q, C, *J* = 309.2 Hz, SCF_3), 128.8 (2 x CH, Fmoc arom.), 128.4 (C, C1 Dmt), 128.2 (CH, Fmoc arom.), 126.3 (CH, Fmoc arom.), 126.3 (CH, Fmoc arom.), 120.9 (2 x CH, Fmoc arom.), 116.8 (CH, C5 Dmt), 108.4 (C, C3 Dmt), 68.0 (CH_2, Fmoc CH_2), 55.3 (CH, Cα Dmt), 48.3 (CH, Fmoc CH), 33.6 (CH_2, Cβ Dmt), 21.1 (CH_3, C6-CH_3 Dmt), 19.0 (CH_3, C2-CH_3 Dmt).

^{19}F NMR (565 MHz, MeOD-*d4*): *δ* = –44.42 (s, 3F, SCF_3).

IR (neat, cm^{-1}): 3422, 3307, 2976, 2942, 1699, 1536, 1450, 1321, 1269, 1236, 1207, 1140, 1105, 1044, 1023, 863, 756, 738.

HRMS (ESI) m/z: $[M + H]^+$ Calcd. for $C_{27}H_{25}F_3NO_5S^+$ 532.1400; Found: 532.1396.

Fmoc-L-Trp(2-CF_3)-OH (6a)

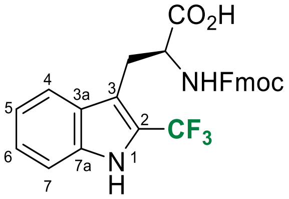

<u>**Radical trifluoromethylation of Fmoc-L-Trp-OH (2a)**</u>

Synthesized following a slightly modified literature procedure.[10] A round-bottom flask was charged with Fmoc-L-Trp-OH **2a** (213 mg, 0.50 mmol, 1.0 equiv.), CF_3SO_2Na (234 mg, 1.50 mmol, 3.0 equiv.), $(NH_4)_2S_2O_8$ (228 mg, 1.00 mmol, 2.0 equiv.), $Cu(OAc)_2$ (10 mg, 0.055 mmol, 0.01 equiv.), and DMSO (2 mL, 0.25 M). The suspension was stirred at 40 °C for 24 hours under ambient atmosphere (open flask). The crude mixture was directly loaded on the silica gel column (mobile phase: *n*-hex/EtOAc/AcOH 50:50:2) and purified, affording 32 mg (0.065 mmol, 13%) of pure **6a** as a yellow solid after several co-evaporations to remove excess AcOH.

<u>***Photomediated trifluoromethylation of Fmoc-L-Trp-OH (2a)***</u>

Synthesized following a literature procedure.[11] A 4 mL reaction vial was charged with Fmoc-L-Trp-OH **2a** (128 mg, 0.30 mmol, 1.0 equiv.), CF_3SO_2Na (93.6 mg, 0.60 mmol, 2.0 equiv.), and DMSO (2 mL, 0.15 M). The reaction vial was open to air and placed above a 395–410 nm custom made LED array (each LED has 3 W electrical power) and stirred under irradiation at ambient temperature (ca. 30 °C due to heat generation from LEDs) for 24 hours. The reaction mixture was diluted with EtOAc (20 mL) and washed sequentially with 0.1 M HCl (aq., 4 x 20 mL) and brine (1 x 20 mL). The organic phase was dried over anhydrous Na_2SO_4, filtered, and concentrated *in vacuo*. The crude product was purified by silica gel flash CC (dry loading; mobile phase: *n*-hex/EtOAc/AcOH 50:50:2), affording 59 mg (0.12 mmol, 40%) of pure **6a**.

mp 101–103 °C

$[\alpha]_D^{20}$ = –6.67 (*c* 0.135, MeOH)

^{1}H NMR (600 MHz, $CDCl_3$) of the major rotamer: δ = 8.48 (bs, 1H, N1-H Trp), 7.77–7.70 (m, 3H, C4-H Trp and Fmoc arom.), 7.53–7.48 (m, 2H, Fmoc arom.), 7.41–7.34 (m, 4H, C7-H Trp and Fmoc arom.), 7.33–7.26 (m, 3H, C6-H Trp and Fmoc arom.), 7.20–7.14 (m, 1H, C5-H Trp), 5.38 (d, *J* = 8.4 Hz, 1H, NH amide), 4.79–4.72 (m, 1H, Cα-H Trp), 4.31 (d, *J* = 7.3 Hz, 2H, Fmoc CH_2), 4.14 (t, *J* = 7.4 Hz, 1H, Fmoc CH), 3.52 (dd, *J* = 14.9, 5.9 Hz, 1H, Cβ-Hb Trp), 3.39 (dd, *J* = 14.8, 7.5 Hz, 1H, Cβ-Ha Trp).

$^{13}C\{^1H\}$ NMR (151 MHz, $CDCl_3$): δ = 175.9 (C=O, CO_2H), 156.0 (C=O, Fmoc), 143.9 (C, Fmoc arom.), 143.8 (C, Fmoc arom.), 141.4 (C, Fmoc arom.), 135.4 (C, C7a Trp), 127.8 (2 x CH, Fmoc arom.), 127.4 (C, C3a Trp), 127.2 (2 x CH, Fmoc arom.), 125.4–125.2 (m, 3 x CH, C6 Trp and Fmoc arom.), 123.0 (q, C, *J* = 36.7 Hz, C2 Trp), 121.9 (q, C, *J* = 269.0 Hz, CF_3), 121.4

(CH, C5 Trp), 120.2 (CH, C4 Trp), 120.1 (2 x CH, Fmoc arom.), 112.2 (C, C3 Trp), 112.1 (C, C7 Trp), 67.5 (CH_2, Fmoc CH_2), 54.3 (CH, Cα Trp), 47.1 (CH, Fmoc CH), 27.2 (CH_2, Cβ Trp).

^{19}F NMR (565 MHz, $CDCl_3$): δ = –57.66 (s, 3F, CF_3).

IR (neat, cm^{-1}): 3404, 3297, 2922, 2853, 1703, 1516, 1318, 1252, 1219, 1161, 1113, 1078, 1054, 738.

HRMS (ESI) m/z: $[M + Na]^+$ Calcd. for $C_{27}H_{21}F_3N_2O_4Na^+$ 517.1346; Found: 517.1344.

Fmoc-L-Trp(2-CF$_3$)-OMe (6b)

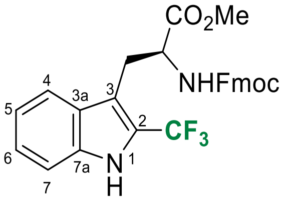

Synthesized following a slightly modified literature procedure.[10] A round-bottom flask was charged with Fmoc-L-Trp-OMe **2b** (110 mg, 0.25 mmol, 1.0 equiv.), CF_3SO_2Na (117 mg, 0.75 mmol, 3.0 equiv.), $(NH_4)_2S_2O_8$ (114 mg, 0.50 mmol, 2.0 equiv.), $Cu(OAc)_2$ (5 mg, 0.028 mmol, 0.01 equiv.), and DMSO (1 mL, 0.25 M). The suspension was stirred at 40 °C for 24 hours under ambient atmosphere (open flask). The crude mixture was directly loaded on the silica gel column (mobile phase: *n*-hex/EtOAc 1:1) and purified, affording pure **6b**.

Yield: 41.2 mg (0.081 mmol, 32%; mixture of rotamers: 86:14) of off-white solid.

mp 121–124 °C

$[\alpha]_D^{20}$ = +2.61 (*c* 0.115, DCM)

^{1}H NMR (600 MHz, $CDCl_3$) of the major rotamer: δ = 8.43 (s, 1H, N1-H Trp), 7.76 (d, *J* = 7.6 Hz, 2H, Fmoc arom.), 7.73 (d, *J* = 8.2 Hz, 1H, C4-H Trp), 7.54 (dd, *J* = 10.8, 7.5 Hz, 2H, Fmoc arom.), 7.41–7.37 (m, 3H, C7-H Trp and Fmoc arom.), 7.33 (ddd, *J* = 8.2, 6.9, 1.1 Hz, 1H, C6-H Trp), 7.29 (dd, *J* = 7.5, 1.3 Hz, 2H, Fmoc arom.), 7.20 (ddd, *J* = 8.0, 6.9, 1.0 Hz, 1H, C5-H Trp), 5.43 (d, *J* = 8.5 Hz, 1H, NH amide), 4.75 (dt, *J* = 8.4, 6.5 Hz, 1H, Cα-H Trp), 4.32 (dd, *J* = 7.3, 2.3 Hz, 2H, Fmoc CH_2), 4.16 (t, *J* = 7.2 Hz, 1H, Fmoc CH), 3.68 (s, 3H, CO_2Me), 3.48–3.43 (m, 1H, Cβ-Hb Trp), 3.43–3.35 (m, 1H, Cβ-Ha Trp).

^{13}C{^{1}H} NMR (151 MHz, $CDCl_3$): δ = 172.2 (C=O, CO_2Me), 155.8 (C=O, Fmoc), 144.0 (C, Fmoc arom.), 143.9 (C, Fmoc arom.), 141.4 (2 x C, Fmoc arom.), 135.4 (C, C7a Trp), 127.8 (2 x CH, Fmoc arom.), 127.5 (C, C3a Trp), 127.2 (2 x CH, Fmoc arom.), 125.3 (3 x CH, C6 Trp and Fmoc arom.), 122.8 (q, C, *J* = 36.6 Hz, C, C2 Trp), 121.9 (q, C, *J* = 268.8 Hz, CF_3), 121.3 (CH, C5 Trp), 120.3 (CH, C4 Trp), 120.1 (2 x CH, Fmoc arom.), 112.5 (C, C3 Trp), 112.0 (C, C7 Trp), 67.3 (CH_2, Fmoc CH_2), 54.4 (CH, Cα Trp), 52.6 (CH_3, CO_2Me), 47.2 (CH, Fmoc CH), 27.6 (CH_2, Cβ Trp).

^{19}F NMR (565 MHz, $CDCl_3$): δ = –57.90 (s, 3F, CF_3).

IR (neat, cm^{-1}): 3325, 2952, 1742, 1695, 1519, 1450, 1319, 1256, 1210, 1160, 1106, 1079, 1056, 737.

HRMS (ESI) m/z: $[M + H]^+$ Calcd. for $C_{28}H_{24}F_3N_2O_4^+$ 509.1683; Found: 509.1671.

Mono- and bis-trifluoromethylthiolation of Boc-L-Tyr-OMe (4c)

Synthesized following a slightly modified literature procedure.[10] Using Boc-L-Tyr-OMe **4c** (872 mg, 2.95 mmol, 1.0 equiv.), CF_3SO_2Na (1.383 g, 8.86 mmol, 3.0 equiv.), $(NH_4)_2S_2O_8$ (1.346 g, 5.90 mmol, 2.0 equiv.), $Cu(OAc)_2$ (53.6 mg, 0.295 mmol, 0.01 equiv.), DMSO (11.8 mL, 0.25 M), 40 °C, 24 h, open flask. After 24 hours, the crude reaction mixture was analyzed by ^{1}H and ^{19}F NMR spectroscopy and another batch of reagents were added: CF_3SO_2Na (692 mg, 4.43 mmol, 1.5 equiv.), $(NH_4)_2S_2O_8$ (673 mg, 2.95 mmol, 1.0 equiv.), $Cu(OAc)_2$ (27 mg, 0.15 mmol, 0.005 equiv.). After additional 24 hours of stirring at 40 °C under ambient atmosphere, the reaction mixture was filtered through a pad of silica gel (mobile phase: EtOAc/cy-hex 1:1) to remove DMSO and non-polar impurities, and the fractions containing **7a** and **7b** were combined and concentrated *in vacuo*. The crude residue was purified by silica gel CC (dry loading; mobile phase: EtOAc/cy-hex 1:2), affording separated **7a** and **7b**.

Boc-L-Tyr(3-CF$_3$)-OMe (7a)

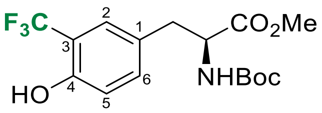

Yield: 294 mg (0.81 mmol, 27%) of yellow semi-solid.

$[\alpha]_D^{20}$ = +20.31 (*c* 0.225, DCM)

^{1}H NMR (400 MHz, CDCl$_3$) of the major rotamer: δ = 7.25–7.22 (m, 1H, C2-H Tyr), 7.13 (d, J = 8.4 Hz, 1H, C6-H Tyr), 6.94 (bs, 1H, OH), 6.76 (d, J = 8.4 Hz, 1H, C5-H Tyr), 5.12 (d, J = 8.4 Hz, 1H, NH amide), 4.59–4.50 (m, 1H, Cα-H Tyr), 3.74 (s, 3H, CO_2Me), 3.09 (dd, J = 14.0, 5.6 Hz, 1H, Cβ-Hb Tyr), 2.95 (dd, J = 14.0, 6.5 Hz, 1H, Cβ-Ha Tyr), 1.41 (s, 9H, Boc).

^{13}C{^{1}H} NMR (101 MHz, CDCl$_3$): δ = 172.5 (C=O, CO_2Me), 155.4 (C=O, Boc), 153.5 (C, C4 Tyr), 134.1 (C, Tyr arom.), 127.7 (2 x CH, Tyr arom.) 124.0 (q, C, J = 272.0 Hz, CF_3), 117.8 (CH, Tyr arom.), 116.7 (q, C, J = 30.5 Hz, C3 Tyr), 80.7 (C, Boc), 54.7 (CH, Cα Tyr), 52.6 (CH_3, CO_2Me), 37.7 (CH_2, Cβ Tyr), 28.4 (3 x CH_3, Boc).

^{19}F NMR (376 MHz, CDCl$_3$): δ = –61.49 (s, 3F, CF_3).

IR (neat, cm^{-1}): 3416, 3359, 2984, 2953, 2118, 1736, 1679, 1528, 1435, 1302, 1149, 1118, 1075, 999.

HRMS (ESI) m/z: $[M + Na]^+$ Calcd. for $C_{16}H_{20}F_3NO_5Na^+$ 386.1186; Found: 386.1189.

Boc-L-Tyr(3,5-CF$_3$)-OMe (7b)

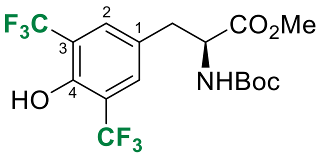

Yield: 332 mg (0.77 mmol, 26%) of white solid.

mp 87–90 °C

$[\alpha]_D^{20}$ = +36.25 (*c* 0.08, DCM)

^{1}H NMR (400 MHz, CDCl$_3$): δ = 7.47 (s, 2H, 2 x C2-H Tyr), 6.10 (bs, 1H, OH), 5.07 (d, *J* = 7.8 Hz, 1H, NH amide), 4.61–4.51 (m, 1H, Cα-H Tyr), 3.75 (s, 3H, CO_2Me), 3.22 (dd, *J* = 14.1, 5.7 Hz, 1H, Cβ-Hb Tyr), 3.03 (dd, *J* = 14.1, 6.0 Hz, 1H, Cβ-Ha Tyr), 1.42 (s, 9H, Boc).

^{13}C{^{1}H} NMR (101 MHz, CDCl$_3$): δ = 171.7 (C=O, CO_2Me), 155.0 (C=O, Boc), 151.0 (C, C4 Tyr), 131.7 (q, 2 x C, *J* = 4.9 Hz, C2 Tyr), 129.0 (C, C1 Tyr), 123.4 (q, 2 x C, *J* = 272.8 Hz, CF_3), 118.9 (q, 2 x C, *J* = 31.2 Hz, C3 Tyr), 80.5 (C, Boc), 54.3 (CH, Cα Tyr), 52.7 (CH_3, CO_2Me), 37.3 (CH_2, Cβ Tyr), 28.3 (3 x CH_3, Boc).

^{19}F NMR (376 MHz, CDCl$_3$): δ = –60.99 (s, 6F, CF_3).

IR (neat, cm^{-1}): 3414, 3349, 2993, 2957, 1732, 1680, 1526, 1492, 1437, 1344, 1282, 1254, 1109, 1076, 1059, 836, 683.

HRMS (ESI) m/z: [(M – Boc) + H]$^+$ Calcd. for $C_{12}H_{12}F_6NO_3^+$ 332.0716; Found: 332.0717.

Boc-L-Tyr(3-CF$_3$)-OH (8a)

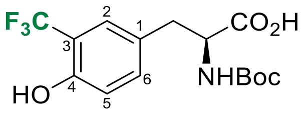

A round-bottom flask equipped with a magnetic stir bar was charged with **7a** (254 mg, 0.70 mmol, 1.0 equiv.) and dissolved in THF/H_2O 1:1 (7 mL). The mixture was stirred and cooled in an ice bath before the addition of LiOH (26 mg, 1.09 mmol, 1.55 equiv.). The reaction mixture was allowed to warm to room temperature and was stirred for 4 hours. After TLC monitoring, which showed residual starting material **7a**, additional LiOH (17 mg, 0.70 mmol, 1.0 equiv.) was added at 0 °C. The reaction mixture was allowed to warm to room temperature and was stirred overnight for a total of 24 hours. The pH was adjusted to 2–3 with 1 M HCl (aq.), THF was removed *in vacuo*, and the aqueous layer was extracted with EtOAc (3 x 35 mL). The combined organic phase was dried over anhydrous Na_2SO_4, concentrated *in vacuo*, and purified using silica gel CC (mobile phase: 1. EtOAc/cy-hex 1:1; 2. DCM/MeOH 10:1 → 5:1), affording pure **8a**.

Yield: 103 mg (0.29 mmol, 42%) of yellowish solid.

mp 88–91 °C

^{1}H NMR (400 MHz, MeOD-*d$_4$*): δ = 7.34 (d, *J* = 2.0 Hz, 1H, C2-H Tyr), 7.25 (dd, *J* = 8.4, 2.1 Hz, 1H, C6-H Tyr), 6.85 (d, *J* = 8.3 Hz, 1H, C5-H Tyr), 4.26 (bs, 1H, Cα-H Tyr), 3.12 (dd, *J* = 14.0, 4.5 Hz, 1H, Cβ-Hb Tyr), 2.85 (dd, *J* = 13.9, 8.5 Hz, 1H, Cβ-Ha Tyr), 1.38 (s, 9H, Boc).

^{13}C{^{1}H} NMR (101 MHz, MeOD-*d$_4$*): δ = 157.7 (C=O, Boc), 155.8 (C, C4 Tyr), 135.2 (CH, C6 Tyr), 129.4 (C, C1 Tyr), 128.6 (q, C, *J* = 5.2 Hz, C2 Tyr), 125.5 (q, C, *J* = 271.4 Hz, CF_3), 117.7 (CH, C5 Tyr), 117.6 (q, C, *J* = 29.7 Hz, C3 Tyr), 80.4 (C, Boc), 56.8 (CH, Cα Tyr), 37.9 (CH_2, Cβ Tyr), 28.7 (3 x CH_3, Boc).

CO_2H resonance could not be observed.

^{19}F NMR (376 MHz, MeOD-*d$_4$*): δ = –63.68 (s, 3F, CF_3).

IR (neat, cm^{-1}): 2980, 1680, 1622, 1513, 1443, 1395, 1369, 1323, 1305, 1273, 1202, 1159, 1121, 1053, 1027, 827.

HRMS (ESI) m/z: [(M – Boc) + Na]$^+$ Calcd. for $C_{10}H_{10}F_3NO_3Na^+$ 272.0505; Found: 272.0504.

Boc-L-Tyr(3,5-CF_3)-OH (8b)

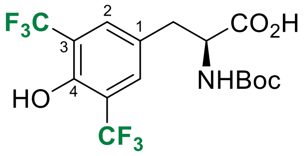

A round-bottom flask equipped with a magnetic stir bar was charged with **7b** (216 mg, 0.50 mmol, 1.0 equiv.) and dissolved in THF/H_2O 1:1 (5 mL). The mixture was stirred and cooled in an ice bath before the addition of LiOH (18 mg, 0.75 mmol, 1.5 equiv.). The reaction mixture was allowed to warm to room temperature and was stirred for 6 hours. After TLC monitoring, which showed residual starting material **7b**, additional LiOH (12 mg, 0.50 mmol, 1.0 equiv.) was added at 0 °C. The reaction mixture was allowed to warm to room temperature and was stirred overnight for a total of 24 hours. The pH was adjusted to 2–3 with 1 M HCl (aq.), THF was removed *in vacuo*, and the aqueous layer was extracted with EtOAc (3 x 30 mL). The combined organic phase was dried over anhydrous Na_2SO_4, concentrated *in vacuo*, and purified using silica gel CC (mobile phase: 1. EtOAc/cy-hex 1:1; 2. DCM/MeOH 10:1 → 5:1), affording pure **8b**.

Yield: 120 mg (0.29 mmol, 58%) of yellowish solid.

mp 82–85 °C

$[\alpha]_D^{20}$ = +9.33 (*c* 0.075, MeOH)

^{1}H NMR (400 MHz, MeOD-*d₄*): δ = 7.64 (s, 2H, 2 x C2-H Tyr), 4.29 (bs, 1H, Cα-H Tyr), 3.27–3.17 (m, 1H, Cβ-Hb Tyr), 2.92 (dd, *J* = 14.0, 8.6 Hz, 1H, Cβ-Ha Tyr), 1.37 (s, 9H, Boc).

^{13}C{^{1}H} NMR (101 MHz, MeOD-*d₄*): δ = 157.6 (C=O, Boc), 153.3 (C, C4 Tyr), 132.6 (q, 2 x C, *J* = 5.1 Hz, C2 Tyr), 131.3 (C, C1 Tyr), 124.9 (q, 2 x C, *J* = 271.9 Hz, CF_3), 121.8 (q, 2 x C, *J* = 30.3 Hz, C3 Tyr), 80.5 (C, Boc), 56.6 (CH, Cα Tyr), 37.9 (CH_2, Cβ Tyr), 28.6 (3 x CH_3, Boc). CO_2H resonance could not be observed.

^{19}F NMR (376 MHz, MeOD-*d₄*): δ = –63.08 (s, 6F, CF_3).

IR (neat, cm^{-1}): 2979, 2929, 1691, 1630, 1493, 1371, 1279, 1257, 1125, 1075, 683.

HRMS (ESI) m/z: [(M–Boc) + H]$^+$ Calcd. for $C_{11}H_{10}F_6NO_3^+$ 318.0559; Found: 318.0559.

Fmoc-L-Tyr-ΨPro(CF_3)-OBn (11)

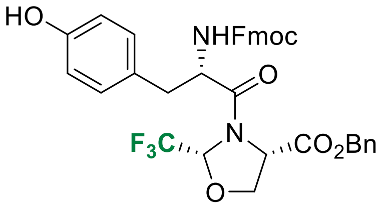

Synthesized by adapting a literature procedure.[12] A round-bottom flask equipped with a magnetic stir bar was charged with Fmoc-L-Tyr(*t*-Bu)-OH (**4d**) (688 mg, 1.50 mmol, 1.5 equiv.) and freshly distilled DCM (12 mL) under argon. $SOCl_2$ (1.42 mL, 19.6 mmol, 19 equiv.) was

added dropwise and the resulting mixture was sonicated at room temperature for 2 hours. The solvent and excess $SOCl_2$ were removed *in vacuo*, affording tyrosine acyl chloride as a white solid, which was immediately employed in peptide coupling without further purification. Benzyl (2*R*,4*S*)-2-(trifluoromethyl)oxazolidine-4-carboxylate (**10**) (275 mg, 1.0 mmol, 1.0 equiv.) was suspended in distilled DCM (10 mL) and slowly added to the Tyr-containing flask under argon. The resulting mixture was stirred overnight at room temperature. Following the reaction, the volatile components were removed *in vacuo* and the residue was purified using silica gel CC (mobile phase: EtOAc/cy-hex 1:2), affording pure **11**.

Yield: 504 mg (0.76 mmol, 76%; mixture of *cis*/*trans* rotational isomers: 40:60; determined by ^{1}H–^{1}H NOESY) of white foamy solid.

mp 84–86 °C

^{1}H NMR (400 MHz, $CDCl_3$) of the *trans* rotamer: δ = 7.73 (d, *J* = 7.6 Hz, 2H, Fmoc arom.), 7.48–7.14 (m, 11H, Fmoc arom. and Bn arom.), 7.04 (d, *J* = 7.8 Hz, 2H, 2 x C2-H Tyr), 6.76 (d, *J* = 8.0 Hz, 2H, 2 x C3-H Tyr), 6.27 (q, *J* = 5.2 Hz, 1H, Cδ-H ΨPro), 5.64 (d, *J* = 8.8 Hz, 1H, NH amide), 5.35 (s, 1H, OH), 5.26–5.03 (m, 3H, Cα-H ΨPro and Bn CH_2), 4.48 (m, 2H, Cα-H Tyr and Cβ-Hb ΨPro), 4.40–4.07 (m, 4H, Cβ-Ha ΨPro, Fmoc CH and Fmoc CH_2), 3.11–2.96 (m, 1H, Cβ-Hb Tyr), 2.93–2.75 (m, 1H, Cβ-Ha Tyr).

^{1}H NMR (400 MHz, $CDCl_3$) of the *cis* rotamer: 7.78 (d, *J* = 7.8 Hz, 2H, Fmoc arom.), 7.58 (d, *J* = 7.4 Hz, 2H, Fmoc arom.), 7.48–7.14 (m, 9H, Fmoc arom. and Bn arom.), 7.04 (d, *J* = 7.8 Hz, 2H, 2 x C2-H Tyr), 6.76 (d, *J* = 8.0 Hz, 2H, 2 x C3-H Tyr), 5.75 (q, *J* = 6.0 Hz, 1H, Cδ-H ΨPro), 5.54 (s, 1H, OH), 5.33 (d, *J* = 7.7 Hz, 1H, NH amide), 5.26–5.03 (m, 2H, Bn CH_2), 4.79–4.68 (m, 1H, Cα-H Tyr), 4.40–4.07 (m, 4H, Cβ-Hb ΨPro, Fmoc CH and Fmoc CH_2), 3.86–3.77 (m, 1H, Cα-H ΨPro), 3.68–3.57 (m, 1H, Cβ-Ha ΨPro), 3.11–2.96 (m, 1H, Cβ-Hb Tyr), 2.93–2.75 (m, 1H, Cβ-Ha Tyr).

^{13}C{^{1}H} NMR (101 MHz, $CDCl_3$): δ = 172.3 (C=O, Tyr-ΨPro *trans*), 171.5 (C=O, Tyr-ΨPro *cis*), 168.0 (C=O, CO_2Bn *trans*), 167.9 (C=O, CO_2Bn *cis*), 156.6 (C, C4 Tyr *trans*), 155.4 (C, C4 Tyr *cis*), 155.3 (C=O, Fmoc *cis*), 155.1 (C=O, Fmoc *trans*), 143.9 (C arom.), 143.7 (C arom.), 143.7 (C arom.), 143.5 (C arom.), 141.4 (C arom.), 135.0 (C arom.), 134.5 (C arom.), 130.6 (CH, C2 Tyr *cis*), 130.5 (CH, C2 Tyr *trans*), 128.8 (C arom.), 128.7 (C arom.), 128.4 (C arom.), 128.2 (C arom.), 127.9 (C arom.), 127.6 (C arom.), 127.2 (2 x C, C1 Tyr *trans* and *cis*), 125.2 (2 x CH, Fmoc arom. *trans*), 125.1 (2 x CH, Fmoc arom. *cis*), 120.2 (4 x CH, Fmoc arom. *trans* and *cis*), 116.1 (C, C3 Tyr *cis*), 115.8 (C, C3 Tyr *trans*), 85.2 (q, CH, *J* = 35.7 Hz, Cδ ΨPro *trans*), 84.2 (q, CH, *J* = 37.2 Hz, Cδ ΨPro *cis*), 70.0 (CH_2, Cβ ΨPro *cis*), 69.1 (CH_2, Cβ ΨPro *trans*), 68.5 (CH_2, Bn CH_2 *cis*), 67.9 (CH_2, Bn CH_2 *trans*), 67.6 (CH_2, Fmoc CH_2 *trans*), 67.4 (CH_2, Fmoc CH_2 *cis*), 56.9 (2 x CH, Cα ΨPro *trans* and *cis*), 54.8 (CH, Cα Tyr *cis*), 54.5 (CH, Cα Tyr *trans*), 47.1 (CH, Fmoc CH *cis*), 47.0 (CH, Fmoc CH *trans*), 40.3 (CH_2, Cβ Tyr *trans*), 37.2 (CH_2, Cβ Tyr *cis*).

Both CF_3 quartet resonances could not be observed.

^{19}F NMR (376 MHz, $CDCl_3$): δ = –78.30 (d, *J* = 5.2 Hz, 3F, CF_3 *trans*), –78.79 (d, *J* = 5.3 Hz, 3F, CF_3 *cis*).

IR (neat, cm^{-1}): 3305, 2962, 1734, 1669, 1515, 1447, 1418, 1214, 1177, 1148, 1116, 1077, 1032, 950, 846, 757, 738, 687.

HRMS (ESI) m/z: $[M + H]^+$ Calcd. for $C_{36}H_{32}F_3N_2O_7^+$ 661.2156; Found: 661.2156.

Fmoc-L-Tyr-ΨPro(CF_3)-OH (12)

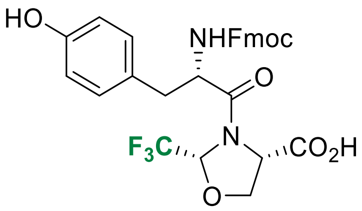

A beaker was equipped with a magnetic stir bar and charged with Fmoc-L-Tyr-ΨPro(CF_3)-OBn (**11**) (453 mg, 0.69 mmol, 1.0 equiv.) and MeOH (7 mL). Pd-C (59 mg, 13 w%) was added under N_2 atmosphere and the beaker was placed in a hydrogenation autoclave. The reactor was filled with H_2 (2 atm) and after 30 minutes, the reaction mixture was filtered through a pad of celite, affording pure **12**.

Yield: 365 mg (0.64 mmol, 93%; mixture of *cis*/*trans* rotational isomers: 50:50; determined by ^1H–^1H ROESY, 14.5 mM) of off-white solid.

mp 124–126 °C

^{1}H NMR (600 MHz, $CDCl_3$) of the *trans* rotamer: δ = 7.70 (d, J = 7.6 Hz, 2H, Fmoc arom.), 7.47–7.40 (m, 2H, Fmoc arom.), 7.38–7.33 (m, 2H, Fmoc arom.), 7.25–7.20 (m, 2H, Fmoc arom.), 7.04 (d, J = 8.0 Hz, 2H, 2 x C2-H Tyr), 6.73 (d, J = 7.8 Hz, 2H, 2 x C3-H Tyr), 6.36–6.30 (m, 1H, Cδ-H ΨPro), 5.99–5.93 (m, 1H, NH amide), 5.02 (t, J = 8.7 Hz, 1H, Cα-H ΨPro), 4.52–4.40 (m, 2H, Cα-H Tyr and Cβ-Hb ΨPro), 4.39–4.14 (m, 3H, Cβ-Ha ΨPro and Fmoc CH_2), 4.11–4.05 (m, 1H, Fmoc CH), 3.14–3.04 (m, 1H, Cβ-Hb Tyr), 2.92–2.79 (m, 1H, Cβ-Ha Tyr).

^{1}H NMR (600 MHz, $CDCl_3$) of the *cis* rotamer: 7.70 (d, J = 7.6 Hz, 2H, Fmoc arom.), 7.47–7.40 (m, 2H, Fmoc arom.), 7.38–7.33 (m, 2H, Fmoc arom.), 7.25–7.20 (m, 2H, Fmoc arom.), 7.04 (d, J = 8.0 Hz, 2H, 2 x C2-H Tyr), 6.73 (d, J = 7.8 Hz, 2H, 2 x C3-H Tyr), 5.93–5.87 (m, 1H, NH amide), 5.78–5.69 (m, 1H, Cδ-H ΨPro), 4.81–4.71 (m, 1H, Cα-H Tyr), 4.39–4.14 (m, 3H, Cβ-Hb ΨPro and Fmoc CH_2), 4.11–4.05 (m, 1H, Fmoc CH), 3.86–3.80 (m, 1H, Cα-H ΨPro), 3.69–3.60 (m, 1H, Cβ-Ha ΨPro), 3.01–2.92 (m, 1H, Cβ-Hb Tyr), 2.92–2.79 (m, 1H, Cβ-Ha Tyr).

^{13}C{^{1}H} NMR (151 MHz, $CDCl_3$): δ = 173.5 (C=O, Tyr-ΨPro *trans*), 171.8 (C=O, Tyr-ΨPro *cis*), 156.8 (C=O, Fmoc *trans*), 156.3 (C=O, Fmoc *cis*), 155.5 (C, C4 Tyr *cis*), 155.2 (C, C4 Tyr *trans*), 143.6 (2 x C, Fmoc arom. *trans*), 143.5 (2 x C, Fmoc arom. *cis*), 141.4 (4 x C, Fmoc arom. *trans* and *cis*), 130.8 (4 x CH, C2 Tyr *trans* and *cis*), 128.0 (4 x CH, Fmoc arom. *trans* and *cis*), 127.3 (4 x CH, Fmoc arom. *trans* and *cis*), 127.2 (2 x C, C1 Tyr *trans* and *cis*), 125.2 (2 x CH, Fmoc arom. *trans*), 125.1 (2 x CH, Fmoc arom. *cis*), 120.2 (4 x CH, Fmoc arom. *trans* and *cis*), 116.1 (2 x CH, C3 Tyr *cis*), 115.7 (2 x CH, C3 Tyr *trans*), 85.2 (q, CH, J = 35.4 Hz, Cδ ΨPro *trans*), 84.3 (q, CH, J = 35.4 Hz, Cδ ΨPro *cis*), 70.1 (CH_2, Cβ ΨPro *cis)*, 68.8 (CH_2, Cβ ΨPro *trans)*, 67.9 (CH_2, Fmoc CH_2 *cis*), 67.7 (CH_2, Fmoc CH_2 *trans*), 57.1 (CH, Cα ΨPro

cis), 57.0 (CH, Cα ΨPro *trans*), 54.8 (CH, Cα Tyr *trans*), 54.7 (CH, Cα Tyr *cis*), 47.0 (2 x CH, Fmoc CH *trans* and *cis*), 39.8 (CH_2, Cβ Tyr *cis*), 37.1 (CH_2, Cβ Tyr *trans*).

CF_3 quartet resonances of both conformers could not be observed.

^{19}F NMR (565 MHz, $CDCl_3$): δ = –78.07 (s, 3F, CF_3 *trans*), –78.53 (bs, 3F, CF_3 *cis*)

IR (neat, cm^{-1}): 3305, 2948, 2167, 1671, 1656, 1613, 1514, 1447, 1248, 1218, 1176, 1148, 1119, 1052, 952, 839, 759, 739, 683.

HRMS (ESI) m/z: $[M + H]^+$ Calcd. for $C_{29}H_{26}F_3N_2O_7^+$ 571.1687; Found: 571.1687.

3. Solid-phase peptide synthesis of opioid ligands

General SPPS procedure

Solid-phase peptide synthesis of fluorinated ligands was performed manually, using standard Fmoc-strategy procotols in plastic syringes containing a frit at the bottom. Fmoc removal was performed twice by using 20% piperidine in DMF (1 mL per 100 mg of resin) for 5 + 15 minutes. Peptide couplings of commercial amino acids were performed using 3 equivalents of the corresponding Fmoc-AA-OH, 3 equivalents of HATU, and 5 equivalents of DIPEA for 45–60 min. With the exception of Fmoc-Dmt(SCF_3)-OH, SCF_3-substituted residues were coupled using 1.5 equivalents of Fmoc-AA(SCF_3)-OH, 3 equivalents of HATU and 3 equivalents of DIPEA for 2–16 hours (overnight without stirring). The coupling with Fmoc-Dmt(SCF_3)-OH (1.0 equivalent) was performed using 1.5 equivalents of Oxyma and 1.5 equivalents of DIC for 3–4 hours. EM1 analogues were synthesized on a Fmoc-Rink amide AM polystyrene resin (0.64 mmol/g). The individual coupling steps were analyzed by Kaiser and chloranil assays, and/or small scale cleavages analyzed by UPLC-MS. Resin cleavage was performed using a cleavage solution of TFA/H_2O/TIPS 95:2.5:2.5 (v/v/v) (1 mL per 100 mg of resin) at room temperature for 2.5 hours. Afterwards, the vessel was washed with DCM (2 x 3 mL) and the volatile components were evaporated using compressed air flow. The crude peptides were then precipitated in cold Et_2O, resolubilized, and freeze-dried. The crude products were purified by semi-preparative HPLC. All peptides were obtained as triflate salts with purity greater than 95% (RP-HPLC analysis, detection at λ = 210 nm). The respective *cis*/*trans* populations of ligands that were obtained in sufficient quantities were determined by ^1H–^1H NOESY or ^1H–^1H ROESY experiments in MeOH-d_3 or MeOH-d_4.

Opioid ligands

Peptides **EM1**, **L1** and **L6** were synthesized as part of our previous methodology study.[7]

TFA.H-Tyr-Pro-Trp-Phe-NH2 (**EM1**)

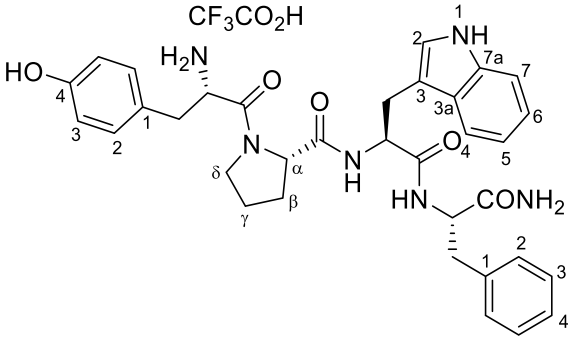

Synthesized following the *General SPPS procedure* using Rink amide AM resin (400 mg, 0.64 mmol/g, 0.256 mmol). The crude peptide was purified using semi-preparative RP-HPLC (80% MQ H_2O + 0.1% TFA/20% MeCN + 0.1% TFA → 60% MeCN + 0.1% TFA, 20 min runs) and freeze dried.

Yield: 44.6 mg (0.062 mmol, 24%) of white solid; *trans*/*cis* = 66:34 (MeOH-d_3, determination by ^{1}H–^{1}H NOESY, 20 mM).

^{1}H NMR (400 MHz, MeOH-d_3, 20 mM) major conformer (*trans*): δ = 10.40–10.33 (m, 1H, N1-H Trp), 7.88 (d, J = 6.7 Hz, 1H, NH Trp amide), 7.66 (d, J = 7.9 Hz, 1H, NH Phe amide), 7.60 (dd, J = 7.7, 1.3 Hz, 1H, CH-4 Trp), 7.30–7.27 (m, 1H, C7-H Trp), 7.27–6.96 (m, 11H, C2-H Tyr, OH Tyr, C2-H Trp, C5-H Trp, C6-H Trp, C2-H Phe, C3-H Phe, C4-H Phe), 6.75 (d, J = 8.5 Hz, 2H, 2 x C3-H Tyr), 4.61–4.55 (m, 1H, Cα-H Trp), 4.52 (dt, J = 7.9, 6.9 Hz, 1H, Cα-H Phe), 4.43 (dd, J = 8.3, 4.9 Hz, 1H, Cα-H Pro), 4.23 (t, J = 7.0 Hz, 1H, Cα-H Tyr), 3.57–3.46 (m, 1H, Cδ-Hb Pro), 3.21 (d, J = 6.4 Hz, 2H, Cβ-H Trp), 3.12–3.02 (m, 1H, Cδ-Ha Pro), 2.95–2.85 (m, 3H, 2 x Cβ-H Phe, Cβ-Hb Tyr), 2.76 (dd, J = 14.4, 7.8 Hz, 1H, Cβ-Ha Tyr), 2.04–1.93 (m, 1H, Cβ-Hb Pro), 1.87–1.78 (m, 2H, 2 x Cγ-H Pro), 1.78–1.69 (m, 1H, Cβ-Ha Pro); **minor conformer (*cis*):** δ = 10.47–10.43 (m, 1H, N1-H Trp), 8.10 (d, J = 7.8 Hz, 1H, NH Phe amide), 7.78 (d, J = 7.7 Hz, 1H, NH Trp amide), 7.60 (dd, J = 7.7, 1.3 Hz, 1H, C4-H Trp), 7.42–7.36 (m, 1H, C7-H Trp), 7.27–6.96 (m, 9H, OH Tyr, C2-H Trp, C5-H Trp, C6-H Trp, C2-H Phe, C3-H Phe, C4-H Phe), 6.89 (d, J = 8.5 Hz, 2H, C2-H Tyr), 6.70 (d, J = 8.5 Hz, 2H, C3-H Tyr), 4.69 (ddd, J = 9.5, 7.8, 5.7 Hz, 1H, Cα-H Trp), 4.61–4.55 (m, 1H, Cα-H Phe), 3.57–3.46 (m, 1H, Cα-H Tyr), 3.38–3.33 (m, 1H, Cδ-Hb Pro), 3.30–3.22 (m, 3H, Cα-H Pro, Cδ-Ha Pro, Cβ-Hb Trp), 3.12–3.02 (m, 2H, Cβ-Ha Trp, Cβ-Hb Phe), 2.95–2.85 (m, 3H, Cβ-Ha Phe, Cβ-H Tyr), 1.54 (ddd, J = 10.6, 7.5, 5.4 Hz, 2H, Cβ-H Pro), 1.49–1.40 (m, 1H, Cγ-Hb Pro), 1.26–1.11 (m, 1H, Cγ-Ha Pro).

^{13}C{^{1}H} NMR (101 MHz, MeOH-d_3, 20 mM) major conformer (*trans*): δ = 175.5 (C=O, $CONH_2$), 173.4 (C=O, Trp-Phe), 173.3 (C=O, Pro-Trp), 169.0 (C=O, Tyr-Pro), 158.2 (C, C4

Tyr), 138.1–138.0 (2 x C, C1 Phe, C7a Trp), 131.7 (2 x CH, C2 Tyr), 130.2 (2 x CH, C3Phe), 129.3 (2 x CH, C2 Phe), 128.7 (C, C3a Trp), 127.6 (CH, C4 Phe), 125.6 (C, C1 Tyr), 125.0 (CH, C2 Trp), 122.5 (CH, C6 Trp), 119.9 (CH, C5 Trp), 119.2 (CH, C4 Trp), 116.7 (2 x CH, C3 Tyr), 112.4 (CH, C7 Trp), 110.2 (C, C3 Trp), 61.5 (CH, Cα Pro), 56.0 (CH, Cα Trp), 55.5 (CH, Cα Phe), 54.6 (CH, Cα Tyr), 48.2 (CH_2, Cδ Pro), 38.3 (CH_2, Cβ Phe), 36.8 (CH_2, Cβ Tyr), 29.8 (CH_2, Cβ Pro), 28.3 (CH_2, Cβ Trp), 25.8 (CH_2, Cγ Pro); **minor conformer (*cis*):** *δ* = 175.7 (C=O, $CONH_2$), 173.6 (C=O, Trp-Phe), 173.1 (C=O, Pro-Trp), 168.9 (C=O, Tyr-Pro), 158.5 (C, C4 Tyr), 138.1–138.0 (2 x C, C1 Phe, C7a Trp), 131.3 (2 x CH, C2 Tyr), 130.2 (2 x CH, C3 Phe), 129.3 (2 x CH, C2 Phe), 128.5 (C, C3a Trp), 127.6 (CH, C4 Phe), 125.2 (C, C1 Tyr), 124.7 (CH, C2 Trp), 122.6 (CH, C6 Trp), 119.9 (CH, C5 Trp), 119.2 (CH, C4 Trp), 116.7 (2 x C, C3 Tyr), 112.4 (CH, C7 Trp), 110.8 (C, C3 Trp), 61.1 (CH, Cα Pro), 55.7 (CH, Cα Trp), 55.6 (CH, Cα Phe), 54.6 (CH, Cα Tyr), 47.9 (CH_2, Cδ Pro), 38.7 (CH_2, Cβ Phe), 38.0 (CH_2, Cβ Tyr), 32.3 (CH_2, Cβ Pro), 28.4 (CH_2, Cβ Trp), 22.7 (CH_2, Cγ Pro).

HRMS (ESI) m/z: $[M + H]^+$ Calcd. for $C_{34}H_{39}N_6O_5^+$ 611.2976; Found: 611.2978.

TFA.H-**Dmt**-Pro-Trp-Phe-NH_2 (**L0**)

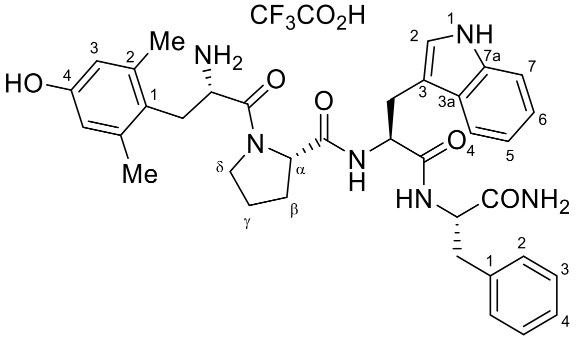

Synthesized following the <u>*General SPPS procedure*</u> using Rink amide AM resin (243 mg, 0.412 mmol/g, 0.1 mmol). The coupling with Fmoc-L-Dmt-OH **4b** was performed using DIC/Oxyma (1.5 equiv. each) for 3–4 hours. The crude peptide was purified using preparative RP-HPLC (70% MQ H_2O + 0.1% TFA/30% MeCN + 0.1% TFA → 70% MeCN + 0.1% TFA) and freeze dried.

Yield: 18.0 mg (0.024 mmol, 24%) of white solid, as a mixture of two rotamers in a ratio of 70:30 in MeOH at 298K.

^{1}H NMR (400 MHz, MeOH-*d*$_4$) **minor rotamer when visible δ* = 7.65* (d, *J* = 8.0 Hz, 0.3H), 7.61 (d, *J* = 7.9 Hz, 0.7H), 7.37 (d, *J* = 8.1 Hz, 0.7H), 7.30-7.18 (m, 5H), 7.14-7.02 (m, 3.3H), 6.54* (s, 0.6H), 6.48 (s, 1.4H), 4.66-4.54 (m, 2.1H), 4.51* (t, *J* = 6.7 Hz, 0.3H), 4.40* (q, *J* = 4.3 Hz, 0.3H), 4.25* (dd, *J* = 8.4, 7.5 Hz, 0.3H), 3.71 (dd, *J* = 12.0, 4.0 Hz, 0.7H), 3.27-3.04 (m, 5.3H), 3.02-2.86 (m, 3H), 2.25* (s, 1.8H), 2.10 (s, 4.2H), 1.80-1.65 (m, 1H), 1.55-1.42 (m, 2H), 1.24-1.11 (m, 1H).

HRMS (ESI) m/z: $[M + H]^+$ Calcd. for $C_{36}H_{43}N_6O_5^+$ 639.3289; Found: 639.3295.

TFA.H-**Tyr(3-SCF$_3$)**-Pro-Trp-Phe-NH$_2$ (**L1**)

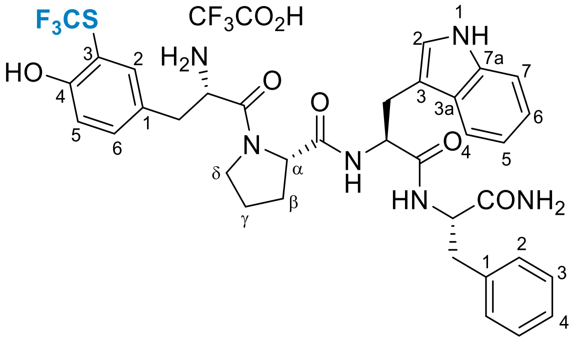

Synthesized following the *General SPPS procedure* using Rink amide AM resin (300 mg, 0.64 mmol/g, 0.192 mmol). The crude peptide was purified using semi-preparative RP-HPLC (70% MQ H_2O + 0.1% TFA/30% MeCN + 0.1% TFA → 70% MeCN + 0.1% TFA, 20 min runs) and freeze dried.

Yield: 29.8 mg (0.036 mmol, 19%) of white solid; *trans*/*cis* = 71:29 (determination by ^{1}H–^{1}H ROESY in MeOH-*d*$_3$, 20 mM).

^{1}H NMR (400 MHz, MeOH-*d*$_3$, 20 mM) major conformer (*trans*): δ = 10.38 (d, *J* = 2.3 Hz, 1H, N1-H Trp), 7.93 (d, *J* = 6.7 Hz, 1H, NH Trp amide), 7.66 (d, *J* = 7.9 Hz, 1H, NH Phe amide), 7.61 (d, *J* = 7.5 Hz, 1H, C4-H Trp), 7.48 (d, *J* = 2.3 Hz, 1H, C2-H Tyr), 7.27 (dd, *J* = 8.1, 0.9 Hz, 1H, C7-H Trp), 7.25–6.95 (m, 10H, OH Tyr, C6-H Tyr, C2-H Trp, C5-H Trp, C6-H Trp, C2-H Phe, C3-H Phe, C4-H Phe), 6.90 (d, *J* = 8.4 Hz, 1H, C5-H Tyr), 4.64–4.55 (m, 1H, Cα-H Trp), 4.55–4.49 (m, 1H, Cα-H Phe), 4.43 (dd, *J* = 8.2, 4.9 Hz, 1H, Cα-H Pro), 4.27 (t, *J* = 6.9 Hz, 1H, Cα-H Tyr), 3.63–3.50 (m, 1H, Cδ-Hb Pro), 3.29–3.14 (m, 2H, Cβ-H Trp), 3.14–3.00 (m, 1H, Cδ-Ha Pro), 2.96–2.85 (m, 3H, Cβ-H Phe, Cβ-Hb Tyr), 2.79 (dd, *J* = 14.5, 7.6 Hz, 1H, Cβ-Ha Tyr), 2.04–1.94 (m, 1H, Cβ-Hb Pro), 1.88–1.79 (m, 2H, Cγ-H Pro), 1.79–1.70 (m, 1H, Cβ-Ha Pro); **minor conformer (*cis*):** δ = 10.46 (s, 1H, N1-H Trp), 8.15 (d, *J* = 7.9 Hz, 1H, NH Trp amide), 7.80 (d, *J* = 7.7 Hz, 1H, NH Phe amide), 7.61 (d, *J* = 7.5 Hz, 1H, C4-H Trp), 7.40–7.35 (m, 2H, OH Tyr, C7-H Trp), 7.30 (d, *J* = 2.3 Hz, 1H, C2-H Tyr), 7.25–6.95 (m, 9H, C6-H Tyr, C2-H Trp, C5-H Trp, C6-H Trp, C2-H Phe, C3-H Phe, C4-H Phe), 6.90 (d, *J* = 8.4 Hz, 1H, C5-H Tyr), 4.74–4.66 (m, 1H, Cα-H Trp), 4.64–4.55 (m, 1H, Cα-H Phe), 3.63–3.50 (m, 1H, Cα-H Tyr), 3.45 (dd, *J* = 7.2, 4.2 Hz, 1H, Cα-H Pro), 3.38–3.32 (m, 1H, Cδ-Hb Pro), 3.29–3.14 (m, 2H, Cδ-Ha Pro, Cβ-Hb Trp), 3.14–3.00 (m, 2H, Cβ-Ha Trp, Cβ-Hb Phe), 2.96–2.85 (m, 3H, Cβ-H Tyr, Cβ-Ha Phe), 1.59–1.49 (m, 2H, Cβ-H Pro), 1.48–1.37 (m, 1H, Cγ-Hb Pro), 1.26–1.12 (m, 1H, Cγ-Ha Pro).

^{13}C{^{1}H} NMR (101 MHz, MeOH-*d*$_3$, 20 mM) major conformer (*trans*): δ = 175.5 (C=O, CONH$_2$), 173.4 (C=O, Trp-Phe), 173.4 (C=O, Pro-Trp), 168.7 (C=O, Tyr-Pro), 160.7 (C, C4 Tyr), 140.68 (CH, C2 Tyr), 138.0 (C, C7a Trp), 138.0 (C, C1 Phe), 135.8 (CH, C6 Tyr), 131.1 (q, C, *J* = 308.0 Hz, SCF$_3$), 130.2 (2 x CH, C2 Phe), 129.3 (2 x CH, C3 Phe), 128.7 (C, C3a Trp), 127.6 (CH, C4 Phe), 126.7 (C, C1 Tyr), 125.0 (CH, C2 Trp), 122.4 (CH, C6 Trp), 119.9 (CH, C5 Trp), 119.2 (CH, C4 Trp), 118.0 (CH, C5 Tyr), 112.4 (CH, C7 Trp), 110.8 (C, C3 Tyr),

110.2 (C, C3 Trp), 61.5 (CH, Cα Pro), 56.0 (CH, Cα Trp), 55.4 (CH, Cα Phe), 54.2 (CH, Cα Tyr), 48.3 (CH_2, Cδ Pro), 38.3 (CH_2, Cβ Phe), 36.2 (CH_2, Cβ Tyr), 29.8 (CH_2, Cβ Pro), 28.4 (CH_2, Cβ Trp), 25.8 (CH_2, Cγ Pro); **minor conformer (*cis*):** *δ* = 175.7 (C=O, $CONH_2$), 173.6 (C=O, Pro-Trp), 172.9 (C=O, Trp-Phe), 168.6 (C=O, Tyr-Pro), 160.8 (C, C4 Tyr), 140.1 (CH, C2 Tyr), 138.1 (C, C7a Trp), 138.0 (C, C1 Phe), 135.2 (CH, C6 Tyr), 130.2 (2 x CH, C2 Phe), 129.3 (2 x CH, C3 Phe), 128.5 (C, C3a Trp), 127.6 (CH, C4 Phe), 126.4 (C, C1 Tyr), 124.7 (CH, C2 Trp), 122.6 (CH, C6 Trp), 119.9 (CH, C5 Trp), 119.2 (CH, C4 Trp), 118.0 (CH, C5 Tyr), 112.5 (CH, C7 Trp), 111.0 (C, C3 Tyr), 110.7 (C, C3 Trp), 61.3 (CH, Cα Pro), 55.8 (CH, Cα Trp), 55.8 (CH, Cα Phe), 54.1 (CH, Cα Tyr), 47.8 (CH_2, Cδ Pro), 38.7 (CH_2, Cβ Phe), 37.3 (CH_2, Cβ Tyr), 32.3 (CH_2, Cβ Pro), 28.5 (CH_2, Cβ Trp), 22.6 (CH_2, Cγ Pro).

SCF_3 quartet resonance of the minor conformer could not be observed.

^{19}F NMR (376 MHz, MeOH-*d₃*, 20 mM): *δ* = –44.25 (3F, SCF_3 minor), –44.30 (3F, SCF_3 major), –77.13 (s, 3F, TFA).

HRMS (ESI) m/z: $[M + H]^+$ Calcd. for $C_{35}H_{38}F_3N_6O_5S^+$ 711.2571; Found: 711.2572.

TFA.H-**Tyr(3-CF_3)**-Pro-Trp-Phe-NH_2 (**L2**)

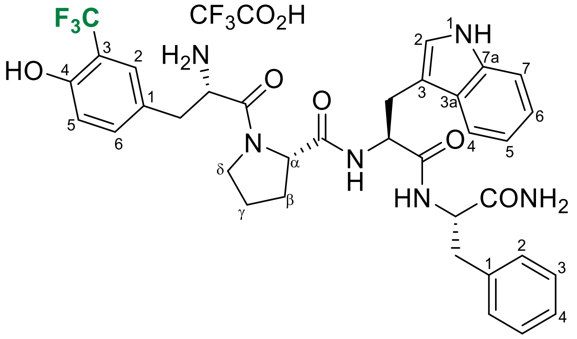

Synthesized following the <u>*General SPPS procedure*</u> using Rink amide AM resin (250 mg, 0.64 mmol/g, 0.16 mmol). The crude peptide was purified using semi-preparative RP-HPLC (70% MQ H_2O + 0.1% TFA/30% MeCN + 0.1% TFA → 45% MeCN + 0.1% TFA, 20 min runs) and freeze dried.

Yield: 16.2 mg (0.020 mmol, 13%) of white solid; *trans*/*cis* = 68:32 (determination by ^{1}H–^{1}H ROESY in MeOD-*d₄*, 20 mM).

^{1}H NMR (600 MHz, MeOD-*d₄*, 20 mM) major conformer (*trans*): *δ* = 7.90 (d, *J* = 6.8 Hz, 1H, NH not exch.), 7.62–7.59 (m, 1H, C4-H Trp), 7.44 (d, *J* = 2.3 Hz, 1H, C2-H Tyr), 7.29–7.05 (m, 9H, C6-H Tyr, C2-H Trp, C6-H Trp, C7-H Trp, C2-H Phe, C3-H Phe, C4-H Phe), 7.03 (ddd, *J* = 8.0, 7.0, 1.1 Hz, 1H, C5-H Trp), 6.91 (d, *J* = 8.4 Hz, 1H, C5-H Tyr), 4.62–4.55 (m, 1H, Cα-H Trp), 4.53 (t, *J* = 6.8 Hz, 1H, Cα-H Phe), 4.45 (dd, *J* = 8.3, 5.1 Hz, 1H, Cα-H Pro), 4.29 (dd, *J* = 8.0, 5.8 Hz, 1H, Cα-H Tyr), 3.63–3.56 (m, 1H, Cδ-Hb Pro), 3.22 (d, *J* = 6.4 Hz, 3H, Cβ-H Trp, Cδ-Ha Pro), 3.01–2.86 (m, 3H, Cβ-Hb Tyr, Cβ-H Phe), 2.80 (dd, *J* = 14.7, 8.0 Hz, 1H, Cβ-Ha Tyr), 2.07–1.98 (m, 1H, Cβ-Hb Pro), 1.95–1.85 (m, 2H, Cγ-H Pro), 1.82–1.75 (m, 1H, Cβ-Ha Pro); **minor conformer (*cis*):** *δ* = 7.82 (d, *J* = 7.8 Hz, 1H, NH not exch.), 7.62–7.59 (m,

1H, C4-H Trp), 7.37 (d, *J* = 8.2 Hz, 1H, C7-H Trp), 7.29–7.03 (m, 10H, C2-H Tyr, C6-H Tyr, C2-H Trp, C5-H Trp, C6-H Trp, C2-H Phe, C3-H Phe, C4-H_Phe), 6.90 (d, *J* = 8.0 Hz, 1H, C5-H Tyr), 4.71–4.64 (m, 1H, Cα-H Trp), 4.62–4.55 (m, 1H, Cα-H Phe), 3.63–3.56 (m, 1H, Cα-H Tyr), 3.40–3.34 (m, 2H, Cα-H Pro, Cδ-Hb Pro), 3.25 (d, *J* = 5.9 Hz, 1H, Cβ-Hb Trp), 3.24–3.17 (m, 1H, Cδ-Ha Pro), 3.11 (dd, *J* = 14.6, 9.4 Hz, 1H, Cβ-Ha Trp), 3.05 (dd, *J* = 13.9, 5.5 Hz, 1H, Cβ-Hb Phe), 3.01–2.86 (m, 3H, Cβ-H Tyr; Cβ-Ha Phe), 1.60–1.53 (m, 2H, Cβ-H Pro), 1.49–1.42 (m, 1H, Cγ-Hb Pro), 1.26–1.19 (m, 1H, Cγ-Ha Pro).

$^{13}C\{^1H\}$ NMR (151 MHz, MeOD-d_4, 20 mM) major conformer (*trans*): δ = 175.5 (C=O, $CONH_2$), 173.5 (C=O, Trp-Phe), 173.4 (C=O, Pro-Trp), 168.8 (C=O, Tyr-Pro), 162.9 (q, *J* = 34.8 Hz, TFA), 157.0 (C, C4 Tyr), 138.2 (C, C1 Phe), 138.0 (C, C7a Trp), 135.9 (CH, C6 Tyr), 130.4 (2 x CH, C2 Phe) 129.5 (2 x CH, C3 Phe), 129.1 (q, CH, *J* = 5.1 Hz, C2 Tyr), 128.9 (C, C3a Trp), 127.8 (CH, C4 Phe), 125.5 (C, C1 Tyr), 125.3 (q, C, *J* = 271.5 Hz, CF_3), 125.0 (CH, C2 Trp), 122.6 (CH, C6 Trp), 120.1 (CH, C5 Trp), 119.4 (CH, C4 Trp), 118.4 (CH, C5 Tyr), 118.3 (q, C, *J* = 31.2 Hz, C3 Tyr), 112.5 (CH, C7 Trp), 110.4 (C, C3 Trp), 61.7 (CH, Cα Pro), 56.0 (CH, Cα Trp), 55.5 (CH, Cα Phe), 54.2 (CH, Cα Tyr), 48.5 (CH_2, Cδ Pro), 38.5 (CH_2, Cβ Phe), 36.3 (CH_2, Cβ Tyr), 30.0 (CH_2, Cβ Pro), 28.5 (CH_2, Cβ Trp), 26.0 (CH_2, Cγ Pro); **minor conformer (*cis*):** δ = 175.7 (C=O, $CONH_2$), 173.7 (C=O, Trp-Phe), 173.0 (C=O, Pro-Trp), 168.7 (C=O, Tyr-Pro), 162.9 (q, *J* = 34.8 Hz, TFA), 157.1 (C, C4 Tyr), 138.3 (C, C1 Phe), 138.0 (C, C7a Trp), 135.5 (CH, C6 Tyr), 130.4 (2 x C, C2 Phe), 129.5 (2 x C, C3 Phe), 128.6 (C, C3a Trp), 127.8 (CH, C4 Phe), 125.2 (C, C1 Tyr), 124.8 (CH, C2 Trp), 122.8 (CH, C6 Trp), 120.1 (CH, C5 Trp), 119.4 (CH, C4 Trp), 118.5 (CH, C5 Tyr), 112.6 (CH, C7 Trp), 110.9 (C, C3 Trp), 61.5 (CH, Cα Pro), 55.9 (CH, Cα Trp), 55.8 (CH, Cα Phe), 54.2 (CH, Cα Tyr), 48.1 (CH_2, Cδ Pro), 38.8 (CH_2, Cβ Phe), 37.5 (CH_2, Cβ Tyr), 32.4 (CH_2, Cβ Pro), 28.6 (CH_2, Cβ Trp), 22.8 (CH_2, Cγ Pro).

CF_3, C2 Tyr and C3 Tyr resonances of the minor conformer could not be observed.

^{19}F NMR (565 MHz, MeOD-d_4, 20 mM): δ = –63.76 (3F, CF_3 major), –63.92 (3F, CF_3 minor), –76.99 (3F, TFA).

HRMS (ESI) m/z: $[M + H]^+$ Calcd. for $C_{35}H_{38}F_3N_6O_5^+$ 679.2850; Found: 679.2850.

TFA.H-**Tyr(3,5-CF$_3$)**-Pro-Trp-Phe-NH$_2$ (**L3**)

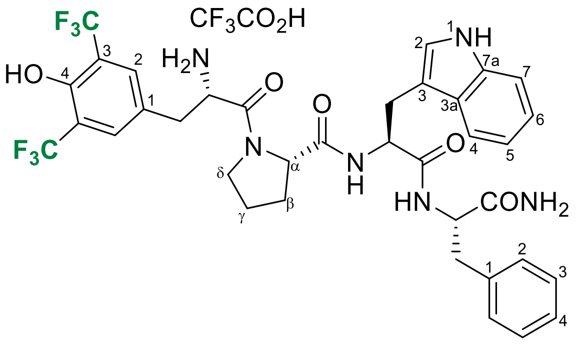

Synthesized following the <u>*General SPPS procedure*</u> using Rink amide AM resin (250 mg, 0.64 mmol/g, 0.16 mmol). The crude peptide was purified using semi-preparative RP-HPLC (70% MQ H_2O + 0.1% TFA/30% MeCN + 0.1% TFA → 55% MeCN + 0.1% TFA, 20 min runs) and freeze dried.

Yield: 30.1 mg (0.035 mmol, 22%) of white solid; *trans* vs. *cis* = 75:25 (determination by $^1H–^1H$ ROESY in MeOD-d_4, 18 mM).

^{1}H NMR (600 MHz, MeOD-d_4, 18 mM) major conformer (*trans*): δ = 7.72 (s, 2H, C2-H Tyr), 7.61 (dd, J = 7.7, 1.1 Hz, 1H, C4-H Trp), 7.26 (dd, J = 8.1, 1.0 Hz, 1H, C7-H Trp), 7.25–7.16 (m, 3H, C3-H Phe, C4-H Phe), 7.13 (s, 1H, C2-H Trp), 7.11–7.09 (m, 2H, C2-H Phe), 7.07 (ddd, J = 8.1, 7.0, 1.3 Hz, 1H, C6-H Trp), 7.03 (ddd, J = 8.0, 7.0, 1.2 Hz, 1H, C5-H Trp), 4.63 (t, J = 6.4 Hz, 1H, Cα-H Trp), 4.52 (dd, J = 7.3, 6.4 Hz, 1H, Cα-H Phe), 4.47 (dd, J = 8.3, 5.1 Hz, 1H, Cα-H Pro), 4.39 (dd, J = 8.5, 5.1 Hz, 1H, Cα-H Tyr), 3.71–3.66 (m, 1H, Cδ-Hb Pro), 3.45–3.37 (m, 1H, Cδ-Ha Pro), 3.28–3.18 (m, 2H, Cβ-H Trp), 2.99 (dd, J = 14.9, 5.1 Hz, 1H, Cβ-Hb Tyr), 2.93–2.82 (m, 3H, Cβ-Ha Tyr, Cβ-H Phe), 2.11–2.03 (m, 1H, Cβ-Hb Pro), 1.95 (ddd, J = 19.3, 12.7, 5.9 Hz, 2H, Cγ-H Pro), 1.81 (dt, J = 12.1, 6.3 Hz, 1H, Cβ-Ha Pro); **minor conformer (*cis*):** δ = 7.61 (dd, J = 7.7, 1.1 Hz, 1H, C4-H Trp), 7.59 (s, 2H, C2-H Tyr), 7.37 (dd, J = 8.2, 0.9 Hz, 1H, C7-H Trp), 7.25–7.16 (m, 5H, C2-H Phe, C3-H Phe, C4-H_Phe), 7.15–7.12 (m, 1H, C6-H Trp), 7.11–7.09 (m, 1H, C2-H Trp), 7.03 (ddd, J = 8.0, 7.0, 1.2 Hz, 1H, C5-H Trp), 4.66 (dd, J = 9.2, 6.2 Hz, 1H, Cα-H Trp), 4.56 (dd, J = 8.6, 5.6 Hz, 1H, Cα-H Phe), 3.73 (dd, J = 8.2, 6.7 Hz, 1H, Cα-H Tyr), 3.65–3.63 (m, 1H, Cα-H Pro), 3.45–3.37 (m, 1H, Cδ-Hb Pro), 3.28–3.18 (m, 2H, Cδ-Ha Pro, Cβ-Hb Trp), 3.13 (dd, J = 14.7, 9.1 Hz, 1H, Cβ-Ha Trp), 3.10–3.02 (m, 3H, Cβ-H Tyr, Cβ-Hb Phe), 2.93–2.82 (m, 1H, Cβ-Ha Phe), 1.73–1.65 (m, 1H, Cβ-Hb Pro), 1.63 (ddd, J = 13.0, 6.9, 3.5 Hz, 1H, Cβ-Ha Pro), 1.49 (dt, J = 11.3, 7.1, 4.0 Hz, 1H, Cγ-Hb Pro), 1.32–1.24 (m, 1H, Cγ-Ha Pro).

^{13}C{^{1}H} NMR (151 MHz, MeOD-d_4, 18 mM) major conformer (*trans*): δ = 175.5 (C=O, $CONH_2$), 173.4 (C=O, Trp-Phe), 173.3 (C=O, Pro-Trp), 168.5 (C=O, Tyr-Pro), 162.8 (q, J = 35.3 Hz, TFA), 154.4 (C, C4 Tyr), 138.2 (C, C1 Phe), 138.0 (C, C7a Trp), 133.4 (q, 2 x CH, J = 5.3 Hz, C2 Tyr), 130.4 (2 x CH, C2 Phe), 129.5 (2 x C, C3 Phe), 128.9 (C, C3a Trp), 127.8 (CH, C4 Phe), 126.9 (C, C1 Tyr), 125.0 (CH, C2 Trp), 124.8 (q, C, J = 272.3 Hz, CF_3), 122.6 (CH, C6_Trp), 122.3 (q, 2 x C, J = 30.8 Hz, C3 Tyr), 120.0 (CH, C5 Trp), 119.4 (CH, C4 Trp), 112.4 (CH, C7 Trp), 110.4 (C, C3 Trp), 61.7 (CH, Cα Pro), 55.7 (CH, Cα Trp), 55.5 (CH, Cα Phe), 53.6 (CH, Cα Tyr), 48.5 (CH_2, Cδ Pro), 38.6 (CH_2, Cβ Phe), 35.7 (CH_2, Cβ Tyr), 30.2 (CH_2, Cβ Pro), 28.4 (CH_2, Cβ Trp), 26.0 (CH_2, Cγ Pro); **minor conformer (*cis*):** δ = 175.7 (C=O, $CONH_2$), 173.7 (C=O, Trp-Phe), 172.9 (C=O, Pro-Trp), 168.4 (C=O, Tyr-Pro), 162.8 (q, J = 35.3 Hz, TFA), 154.4 (C, C4 Tyr), 138.2 (C, C1 Phe), 138.1 (C, C7a Trp), 132.9 (q, 2 x CH, J = 3.7 Hz, C2 Tyr), 130.4 (2 x CH, C2 Phe), 129.5 (2 x CH, C3 Phe), 128.6 (C, C3a Trp), 127.8 (CH, C4 Phe), 126.7 (C, C1 Tyr), 124.8 (CH, C2 Trp), 122.8 (CH, C6 Trp), 120.1 (CH, C5 Trp), 119.3 (CH, C4 Trp), 112.6 (CH, C7 Trp), 110.9 (C, C3 Trp), 61.6 (CH, Cα Pro), 56.1 (CH, Cα Trp), 55.7 (CH, Cα Phe), 53.8 (CH, Cα Tyr), 48.1 (CH_2, Cδ Pro), 38.8 (CH_2, Cβ Phe), 36.8 (CH_2, Cβ Tyr), 32.4 (CH_2, Cβ Pro), 28.6 (CH_2, Cβ Trp), 22.8 (CH_2, Cγ Pro).

CF_3 and C3 Tyr resonances of the minor conformer could not be observed.

^{19}F NMR (565 MHz, MeOD-*d₄*, 18 mM): δ = –63.04 (6F, CF_3 major), –63.22 (6F, CF_3 minor), –77.01 (3F, TFA).

HRMS (ESI) m/z: $[M + H]^+$ Calcd. for $C_{36}H_{37}F_6N_6O_5^+$ 747.2724; Found: 747.2726.

TFA.H-**Dmt(3-SCF$_3$)**-Pro-Trp-Phe-NH_2 (**L4**)

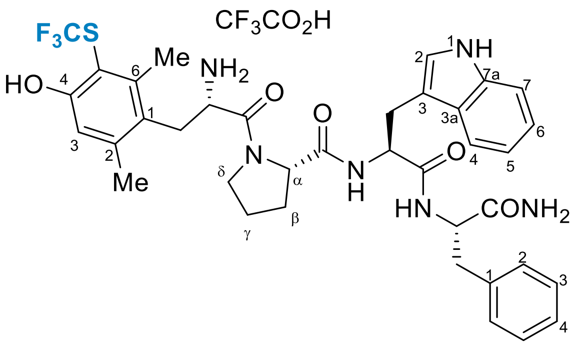

Synthesized following the <u>*General SPPS procedure*</u> using Rink amide AM resin (243 mg, 0.412 mmol/g, 0.10 mmol). The coupling with Fmoc-L-Dmt(3-SCF$_3$)-OH **5b** was performed using DIC/Oxyma (1.5 equiv. each) for 3–4 hours. The crude peptide was purified using preparative RP-HPLC (70% MQ H_2O + 0.1% TFA/30% MeCN + 0.1% TFA → 70% MeCN + 0.1% TFA) and freeze dried.

Yield: 37.0 mg (0.043 mmol, 43%) of white solid, as a mixture of two rotamers in a ratio of 60:40 in MeOH at 298K.

^{1}H NMR (400 MHz, MeOH-*d₄*) ***minor rotamer when visible** δ = 7.65* (d, J = 7.2 Hz, 0.4H), 7.61 (d, J = 7.9 Hz, 0.6H), 7.37 (d, J = 8.1 Hz, 0.6H), 7.33* (d, J = *7.5* Hz, 0.4H), 7.29-7.18 (m, 4.4H), 7.14-6.99 (m, 3.6H), 6.76* (s, 0.4H), 6.73 (s, 0.6H), 4.70-4.57 (m, 1.2H), 4.55* (t, J = 5.6 Hz, 0.4H), 4.50* (t, J = 6.8 Hz, 0.4H), 4.39* (dd, J = 8.3, 4.4 Hz, 0.4H), 4.30* (dd, J = 10.1, 6.0 Hz, 0.4H), 3.80 (dd, J = 12.0, 4.2 Hz, 0.6H), 3.42-2.92 (m, 7.6H), 2.87 (dd, J = 13.7, 7.2 Hz, 0.6H), 2.77* (dd, J = 13.7, 6.1 Hz, 0.4H), 2.59* (s, 1.2H), 2.45 (s, 1.8H), 2.23* (s, 1.2H), 2.18 (s, 1.8H), 1.93-1.83* (m, 0.4H), 1.73-1.57 (m, 1.2H), 1.54-1.45 (m, 0.6H), 1.45-1.30 (m, 1.2H), 1.21-1.05 (m, 0.6H).

^{19}F NMR (376 MHz, MeOD-*d₄*): δ = –44.21 (SCF_3, s, 3F), –44.28* (SCF_3, s, 3F), –77.06 (TFA, 3F).

HRMS (ESI) m/z: $[M + H]^+$ Calcd. for $C_{37}H_{42}F_3N_6O_5S^+$ 739.2884; Found: 739.2861.

TFA.H-Tyr-**ΨPro(CF$_3$)**-Trp-Phe-NH$_2$ (**L5**)

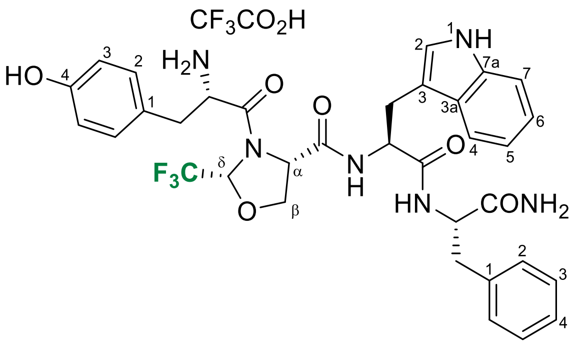

Synthesized following the <u>*General SPPS procedure*</u> using Rink amide AM resin (300 mg, 0.64 mmol/g, 0.192 mmol). The crude peptide was purified using semi-preparative RP-HPLC (80% MQ H_2O + 0.1% TFA/20% MeCN + 0.1% TFA → 60% MeCN + 0.1% TFA, 20 min runs) and freeze dried.

Yield: 18.5 mg (0.023 mmol, 12%) of white solid; *trans*/*cis* = 22:78 (determination by ^{1}H–^{1}H ROESY in MeOD-d_4, 18 mM).

^{1}H NMR (400 MHz, MeOD-d_4, 18 mM) major conformer (*cis*): δ = 7.63 (d, J = 7.9 Hz, 1H, C4-H Trp), 7.41 (d, J = 8.1 Hz, 1H, C7-H Trp), 7.26–7.13 (m, 6H, C6-H Trp, C2-H Phe, C3-H Phe, C4-H Phe), 7.11–7.01 (m, 2H, C5-H Trp, C2-H Trp), 6.89 (d, J = 8.5 Hz, 2H, C2-H Tyr), 6.73 (d, J = 8.5 Hz, 2H, C3-H Tyr), 5.81 (q, J = 5.2 Hz, 1H, Cδ-H ΨPro), 4.74 (dd, J = 9.0, 6.2 Hz, 1H, Cα-H Trp), 4.54 (dd, J = 8.5, 5.7 Hz, 1H, Cα-H Phe), 3.91 (d, J = 7.4 Hz, 2H, Cβ-H ΨPro), 3.60 (dd, J = 9.5, 6.3 Hz, 1H, Cα-H Tyr), 3.51 (t, J = 7.6 Hz, 1H, Cα-H ΨPro), 3.22 (dd, J = 14.7, 6.2 Hz, 1H, Cβ-Hb Trp), 3.12–3.03 (m, 2H, Cβ-Ha Trp, Cβ-Hb Phe), 3.01–2.83 (m, 3H, Cβ-Ha Phe, Cβ-H Tyr).

^{13}C{^{1}H} NMR (101 MHz, MeOD-d_4, 18 mM) of the major conformer (*cis*): δ = 175.6 (C=O, CONH$_2$), 173.2 (C=O, Trp-Phe), 169.2 (C=O, ΨPro-Trp), 158.7 (C, C4 Tyr), 138.2 (C, C1 Phe), 138.1 (C, C7a Trp), 131.5 (2 x CH, C2 Tyr), 130.3 (2 x CH, C2 Phe), 129.4 (2 x CH, C3 Phe), 128.6 (C, C3a Trp), 127.8 (CH, C4 Phe), 124.9 (C, C1 Tyr), 124.8 (CH, C2 Trp), 122.7 (CH, C6 Trp), 120.0 (CH, C5 Trp), 119.5 (CH, C7 Trp), 117.2 (2 x CH, C3 Tyr), 112.5 (CH, C4 Trp), 110.7 (C, C3 Trp), 72.1 (CH$_2$, Cβ ΨPro), 58.9 (CH, Cα ΨPro), 55.8 (CH, Cα Trp), 55.6 (CH, Cα Phe), 54.7 (CH, Cα Tyr), 38.8 (CH$_2$, Cβ Phe), 37.9 (CH$_2$, Cβ Tyr), 29.0 (CH$_2$, Cβ Trp).

CF$_3$ quartet, Cδ ΨPro and C=O Tyr-ΨPro resonances could not be observed.

^{19}F NMR (376 MHz, MeOD-d_4, 18 mM): δ = –76.92 (3F, TFA), –78.71 (s, 3F, CF$_3$ major), –78.79 (bs, 3F, CF$_3$ minor).

HRMS (ESI) m/z: $[M + H]^+$ Calcd. for $C_{34}H_{36}F_3N_6O_6^+$ 681.2643; Found: 681.2640.

TFA.H-Tyr-Pro-**Trp(2-SCF_3)**-Phe-NH_2 (**L6**)

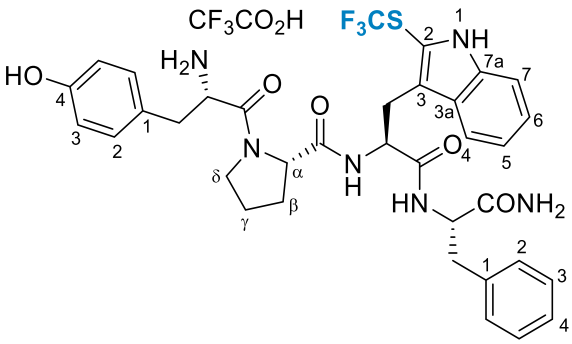

Synthesized following the *General SPPS procedure* using Rink amide AM resin (200 mg, 0.64 mmol/g, 0.128 mmol). The crude peptide was purified using semi-preparative RP-HPLC (70% MQ H_2O + 0.1% TFA/30% MeCN + 0.1% TFA → 70% MeCN + 0.1% TFA, 20 min runs) and freeze dried.

Yield: 27.1 mg (0.033 mmol, 26%) of white solid; *trans*/*cis* = 71:29 (determination by ^1H–^1H ROESY in MeOH-d_3, 20 mM).

^{1}H NMR (400 MHz, MeOH-d_3, 20 mM) major conformer (*trans*): δ = 11.35 (s, 1H, N1-H Trp), 8.02 (d, J = 7.0 Hz, 1H, NH Trp amide), 7.86 (d, J = 8.1 Hz, 1H, NH Phe amide), 7.73 (dd, J = 8.0, 0.9 Hz, 1H, C4-H Trp), 7.35 (d, J = 8.2 Hz, 1H, C7-H Trp), 7.31–6.86 (m, 10H, OH Tyr, C2-H Tyr, C5-H Trp, C6-H Trp, C2-H Phe, C3-H Phe, C4-H Phe), 6.73 (d, J = 8.5 Hz, 2H, C3-H Tyr), 4.65–4.57 (m, 1H, Cα-H Trp), 4.57–4.47 (m, 1H, Cα-H Phe), 4.41 (dd, J = 8.2, 4.7 Hz, 1H, Cα-H Pro), 4.23 (t, J = 7.1 Hz, 1H, Cα-H Tyr), 3.61–3.47 (m, 1H, Cδ-Hb Pro), 3.44–3.30 (m, 2H, Cβ-H Trp), 3.13–2.96 (m, 3H, Cδ-Ha Pro, Cβ-Hb Tyr, Cβ-Hb Phe), 2.94–2.80 (m, 2H, Cβ-Ha Tyr, Cβ-Ha Phe), 1.97 (ddt, J = 12.0, 8.4, 6.5 Hz, 1H, Cβ-Hb Pro), 1.87–1.79 (m, 2H, Cγ-H Pro), 1.76 (dt, J = 13.2, 5.1 Hz, 1H, Cβ-Ha Pro); **minor conformer (*cis*):** δ = 11.48 (s, 1H, N1-H Trp), 8.07 (d, J = 7.9 Hz, 1H, NH Phe amide), 7.85 (d, J = 8.1 Hz, 1H, NH Trp amide), 7.69 (dd, J = 8.1, 1.0 Hz, 1H, C4-H Trp), 7.41 (d, J = 8.3 Hz, 1H, C7-H Trp), 7.33 (s, 1H, OH Tyr), 7.31–6.86 (m, 9H, C2-H Tyr, C5-H Trp, C6-H Trp, C2-H Phe, C3-H Phe, C4-H Phe), 6.71 (d, J = 8.4 Hz, 2H, C3-H Tyr), 4.77–4.68 (m, 1H, Cα-H Trp), 4.57–4.47 (m, 1H, Cα-H Phe), 3.61–3.47 (m, 1H, Cα-H Tyr), 3.44–3.30 (m, 4H, Cδ-Hb Pro, Cβ-H Trp, Cα-H Pro), 3.28–3.20 (m, 1H, Cδ-Ha Pro), 3.13–2.96 (m, 2H, Cβ-Hb Tyr, Cβ-Hb Phe), 2.94–2.80 (m, 2H, Cβ-Ha Tyr, Cβ-Ha Phe), 1.57–1.42 (m, 3H, Cγ-Hb Pro, Cβ-H Pro), 1.23–1.12 (m, 1H, Cγ-Ha Pro).

^{13}C{^{1}H} NMR (101 MHz, MeOH-d_3, 20 mM) of the major conformer (*trans*): δ = 175.2 (C=O, $CONH_2$), 173.1 (C=O, Pro-Trp), 172.6 (C=O, Trp-Phe), 168.9 (C=O, Tyr-Pro), 158.2 (C, C4 Tyr), 139.3 (C, C7a Trp), 138.2 (C, C1 Phe), 131.7 (2 x CH, C2 Tyr), 130.2 (2 x CH, C2 Phe), 130.1 (q, C, J = 310.4 Hz, SCF_3), 129.2 (2 x CH, C3 Phe), 128.2 (C, C3a Trp), 127.5 (CH, C4 Phe), 125.6 (C, C1 Tyr), 125.3 (CH, C6 Trp), 121.6 (C, C3 Trp), 120.9 (CH, C5 Trp), 120.6 (CH, C4 Trp), 116.7 (2 x CH, C3 Tyr), 115.9 (C, C2 Trp), 112.7 (CH, C7 Trp), 61.4 (CH, Cα Pro), 55.8 (CH, Cα Trp), 54.7 (CH, Cα Tyr), 48.3 (CH_2, Cδ Pro), 38.7 (CH_2, Cβ Phe), 37.0 (CH_2, Cβ Tyr), 29.7 (CH_2, Cβ Pro), 28.1 (CH_2, Cβ Trp), 25.9 (CH_2, Cγ Pro); **minor conformer (*cis*):**

δ = 175.3 (C=O, $CONH_2$), 172.9 (C=O, Pro-Trp), 172.8 (C=O, Trp-Phe), 168.9 (C=O, Tyr-Pro), 158.5 (C, C4 Tyr), 139.3 (C, C7a Trp), 138.1 (C, C1 Phe), 131.3 (2 x CH, C2 Tyr), 130.2 (2 x CH, C2 Phe), 129.3 (2 x CH, C3 Phe), 128.2 (C, C3a Trp), 127.6 (CH, C4 Phe), 125.4 (C, C1 Tyr), 125.2 (CH, C6 Trp), 121.5 (C, C3 Trp), 121.0 (CH, C5 Trp), 120.5 (CH, C4 Trp), 116.7 (2 x CH, C3 Tyr), 115.7 (C, C2 Trp), 112.8 (CH, C7 Trp), 61.1 (CH, Cα Pro), 55.7 (CH, Cα Phe), 55.6 (CH, Cα Phe), 54.6 (CH, Cα Tyr), 47.9 (CH_2, Cδ Pro), 38.8 (CH_2, Cβ Phe), 38.1 (CH_2, Cβ Tyr), 32.3 (CH_2, Cβ Pro), 27.8 (CH_2, Cβ Trp), 22.7 (CH_2, Cγ Pro).

SCF_3 quartet resonance of the minor conformer could not be observed.

^{19}F NMR (376 MHz, MeOH-*d*$_3$, 20 mM): δ = –44.93 (3F, SCF_3 minor), –45.08 (3F, SCF_3 major), –77.07 (3F, TFA).

HRMS (ESI) m/z: $[M + H]^+$ Calcd. for $C_{35}H_{38}F_3N_6O_5S^+$ 711.2571; Found: 711.2585.

TFA.H-Tyr-Pro-**Trp(2-CF$_3$)**-Phe-NH_2 (**L7**)

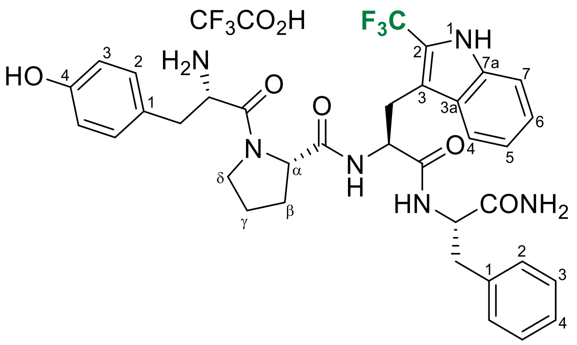

Synthesized following the <u>*General SPPS procedure*</u> using Rink amide AM resin (300 mg, 0.64 mmol/g, 0.192 mmol). The crude peptide was purified using semi-preparative RP-HPLC (80% MQ H_2O + 0.1% TFA/20% MeCN + 0.1% TFA → 60% MeCN + 0.1% TFA, 20 min runs) and freeze dried.

Yield: 34.4 mg (0.044 mmol, 23%) of white solid; *trans*/*cis* = 65:35 (determination by ^{1}H–^{1}H ROESY in MeOD-*d*$_4$, 20 mM).

^{1}H NMR (600 MHz, MeOD-*d*$_4$, 20 mM) major conformer (*trans*): δ = 7.73 (d, *J* = 8.3 Hz, 1H, C4-H Trp), 7.40 (dd, *J* = 8.4, 1.0 Hz, 1H, C7-H Trp), 7.28–7.10 (m, 9H, C2-H Tyr, C5-H Trp, C6-H Trp, C2-H Phe, C3-H Phe, C4-H Phe), 6.79 (d, *J* = 8.5 Hz, 2H, C3-H Tyr), 4.53 (dd, *J* = 8.3, 6.6 Hz, 1H, Cα-H Trp), 4.48 (dd, *J* = 7.6, 6.3 Hz, 1H, Cα-H Phe), 4.38 (dd, *J* = 8.2, 5.5 Hz, 1H, Cα-H Pro), 4.28 (dd, *J* = 7.6, 6.5 Hz, 1H, Cα-H Tyr), 3.57 (dt, *J* = 9.8, 6.7 Hz, 1H, Cδ-Hb Pro), 3.44–3.33 (m, 2H, Cβ-H Trp), 3.15–3.04 (m, 3H, Cβ-Hb Tyr, Cδ-Ha Pro, Cβ-Hb Phe), 2.94–2.87 (m, 2H, Cβ-Ha Tyr, Cβ-Ha Phe), 2.05–1.96 (m, 1H, Cβ-Hb Pro), 1.85 (dt, *J* = 16.4, 6.2 Hz, 2H, Cγ-H Pro), 1.72 (dt, *J* = 12.6, 6.4 Hz, 1H, Cβ-Ha Pro); **minor conformer (*cis*):** δ = 7.72 (d, *J* = 8.0 Hz, 1H, C4-H Trp), 7.43 (d, *J* = 8.3 Hz, 1H, C7-H Trp), 7.30 (ddd, *J* = 8.3, 7.0, 1.1 Hz, 1H, C6-H Trp), 7.28–7.10 (m, 6H, C5-H Trp, C2-H Phe, C3-H Phe, C4-H Phe), 6.96 (d, *J* = 8.5 Hz, 2H, C2-H Tyr), 6.73 (d, *J* = 8.5 Hz, 2H, C3-H Tyr), 4.65 (dd, *J* = 9.2, 6.5 Hz, 1H, Cα-H Trp), 4.56–4.52 (m, 1H, Cα-H Phe), 3.61 (dd, *J* = 9.9, 5.9 Hz, 1H, Cα-H Tyr),

3.44–3.33 (m, 3H, Cα-H Pro, Cδ-Hb Pro, Cβ-Hb Trp), 3.30–3.23 (m, 2H, Cδ-Ha Pro, Cβ-Ha Trp), 3.15–3.04 (m, 1H, Cβ-Hb Phe), 2.96 (dd, *J* = 13.2, 5.9 Hz, 1H, Cβ-Hb Tyr), 2.94–2.87 (m, 2H, Cβ-Ha Phe, Cβ-Ha Tyr), 1.56–1.50 (m, 1H, Cβ-Hb Pro), 1.50–1.44 (m, 2H, Cγ-Hb Pro, Cβ-Ha Pro), 1.18 (ddd, *J* = 14.0, 9.7, 7.3 Hz, 1H, Cγ-Ha Pro).

$^{13}C\{^{1}H\}$ NMR (151 MHz, MeOD-*d₄*, 20 mM) major conformer (*trans*): *δ* = 175.1 (C=O, $CONH_2$), 173.3 (C=O, Pro-Trp), 172.5 (C=O, Trp-Phe), 169.0 (C=O, Tyr-Pro), 162.9 (q, *J* = 34.7 Hz, TFA), 158.4 (C, C4 Tyr), 138.4 (C, C1 Phe), 137.4 (C, C7a Trp), 131.9 (2 x CH, C2 Tyr), 130.5 (2 x CH, C2 Phe), 129.4 (2 x CH, C3 Phe), 128.5 (C, C3a Trp), 127.7 (CH, C4 Phe), 125.8 (CH, C6 Trp), 125.6 (C, C1 Tyr), 124.0 (q, C, *J* = 36.7 Hz, C2 Trp), 123.7 (q, C, *J* = 268.3 Hz, CF_3), 121.4 (CH, C5 Trp), 121.2 (CH, C4 Trp), 116.9 (2 x CH, C3 Tyr), 113.2 (CH, C7 Trp), 113.0 (q, C, *J* = 2.9 Hz, C3 Trp), 61.8 (CH, Cα Pro), 56.5 (CH, Cα Trp), 55.8 (CH, Cα Phe), 54.7 (CH, Cα Tyr), 48.6 (CH_2, Cδ Pro), 38.8 (CH_2, Cβ Phe), 37.0 (CH_2, Cβ Tyr), 30.1 (CH_2, Cβ Pro), 27.3 (CH_2, Cβ Trp), 26.1 (CH_2, Cδ Phe); **minor conformer (*cis*):** *δ* = 175.4 (C=O, $CONH_2$), 173.0 (C=O, Pro-Trp), 172.8 (C=O, Trp-Phe), 169.0 (C=O, Tyr-Pro), 162.9 (q, *J* = 34.7 Hz, TFA), 158.6 (C, C4 Tyr), 138.3 (C, C1 Phe), 137.3 (C, C7a Trp), 131.5 (2 x CH, C2 Tyr), 130.4 (2 x CH, C2 Phe), 129.4 (2 x CH, C3 Phe), 128.5 (C, C3a Trp), 127.8 (CH, C4 Phe), 125.7 (CH, C6 Trp), 125.4 (C, C1 Tyr), 123.7 (q, C, *J* = 268.3 Hz, CF_3), 121.6 (CH, C5 Trp), 121.0 (CH, C4 Trp), 116.9 (2 x CH, C3 Tyr), 113.3 (CH, C7 Trp), 112.9 (q, C, *J* = 2.6 Hz, C3 Trp), 61.3 (CH, Cα Pro), 56.0 (CH, Cα Trp), 55.8 (CH, Cα Phe), 54.6 (CH, Cα Tyr), 48.1 (CH_2, Cδ Pro), 38.9 (CH_2, Cβ Phe), 38.1 (CH_2, Cβ Tyr), 32.4 (CH_2, Cβ Pro), 27.1 (CH_2, Cβ Trp), 22.9 (CH_2, Cγ Pro).

C2 Trp resonance of the minor conformer could not be observed.

^{19}F NMR (565 MHz, MeOD-*d₄*, 20 mM): *δ* = –58.89 (3F, CF_3 major), –58.96 (3F, CF_3 minor), –76.99 (3F, TFA).

HRMS (ESI) m/z: $[M + H]^+$ Calcd. for $C_{35}H_{38}F_3N_6O_5^+$ 679.2850; Found: 679.2849.

TFA.H-Tyr-Pro-Trp-**TFM**-NH_2 (**L8**)

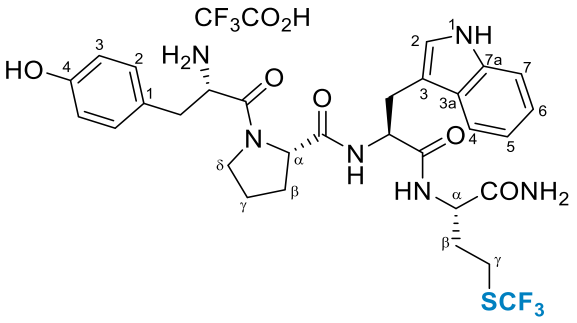

Synthesized following the *General SPPS procedure* using Rink amide AM resin (300 mg, 0.64 mmol/g, 0.192 mmol). The crude peptide was purified using semi-preparative RP-HPLC (80% MQ H_2O + 0.1% TFA/20% MeCN + 0.1% TFA → 60% MeCN + 0.1% TFA, 20 min runs) and freeze dried.

Yield: 14.4 mg (0.019 mmol, 10%) of white solid; *trans*/*cis* = 68:32 (determination by ^{1}H–^{1}H ROESY in MeOD-d_4, 10 mM).

^{1}H NMR (600 MHz, MeOD-d_4, 10 mM) major conformer (*trans*): δ = 8.08 (d, J = 6.4 Hz, 1H, NH not exch.), 7.63 (dd, J = 7.8, 1.1 Hz, 1H, C4-H Trp), 7.30 (d, J = 8.0 Hz, 1H, C7-H Trp), 7.20 (s, 1H, C2-H Trp), 7.11–7.02 (m, 4H, C2-H Tyr, C5-H Trp, C6-H Trp), 6.77 (d, J = 8.5 Hz, 2H, C3-H Tyr), 4.61 (t, J = 6.6 Hz, 1H, Cα-H Trp), 4.52 (dd, J = 8.4, 4.6 Hz, 1H, Cα-H Pro), 4.40 (dd, J = 9.4, 4.6 Hz, 1H, Cα-H TFM), 4.26 (dd, J = 7.8, 6.2 Hz, 1H, Cα-H Tyr), 3.63–3.54 (m, 1H, Cδ-Hb Pro), 3.28 (d, J = 7.0 Hz, 2H, Cβ-H Trp), 3.20–3.13 (m, 1H, Cδ-Ha Pro), 3.03–2.88 (m, 1H, Cβ-Hb Tyr), 2.87–2.72 (m, 3H, Cβ-Ha Tyr, Cγ-H TFM), 2.26–2.13 (m, 1H, Cβ-Hb TFM), 2.13–2.07 (m, 1H, Cβ-Hb Pro), 2.05–1.85 (m, 4H, Cβ-Ha Pro, Cγ-H Pro, Cβ-Ha TFM); **minor conformer (*cis*):** δ = 7.84 (d, J = 7.2 Hz, 1H, NH not exch.), 7.63 (dd, J = 7.8, 1.1 Hz, 1H, C4-H Trp), 7.38 (dd, J = 8.2, 0.9 Hz, 1H, C7-H Trp), 7.16–7.12 (m, 2H, C2-H Trp, C6-H Trp), 7.11–7.02 (m, 1H, C5-H Trp), 6.94 (d, J = 8.5 Hz, 2H, C2-H Tyr), 6.74 (d, J = 8.5 Hz, 2H, C3-H Tyr), 4.72–4.67 (m, 1H, Cα-H Trp), 4.44 (dd, J = 9.4, 4.6 Hz, 1H, Cα-H TFM), 3.63–3.54 (m, 1H, Cα-H Tyr), 3.39 (ddd, J = 11.9, 8.1, 4.0 Hz, 1H, Cδ-Hb Pro), 3.36–3.32 (m, 2H, Cα-H Pro, Cβ-Hb Trp), 3.30–3.25 (m, 1H, Cδ-Ha Pro), 3.20–3.13 (m, 1H, Cβ-Ha Trp), 3.03–2.88 (m, 4H, Cβ-H Tyr, Cγ-H TFM), 2.26–2.13 (m, 1H, Cβ-Hb TFM), 2.05–1.85 (m, 1H, Cβ-Ha TFM), 1.70 (dt, J = 10.3, 3.4 Hz, 1H, Cβ-Hb Pro), 1.67–1.62 (m, 1H, Cβ-Ha Pro), 1.53 (ddd, J = 11.0, 7.2, 4.0 Hz, 1H, Cγ-Hb Pro), 1.34–1.30 (m, 1H, Cγ-Ha Pro).

^{13}C{^{1}H} NMR (151 MHz, MeOD-d_4, 10 mM) major conformer (*trans*): δ = 175.2 (C=O, $CONH_2$), 174.1 (C=O, Trp-TFM), 173.4 (C=O, Pro-Trp), 169.0 (C=O, Tyr-Pro), 158.4 (C, C4 Tyr), 138.0 (C, C7a Trp), 132.7 (q, C, J = 305.1 Hz, SCF_3), 131.9 (2 x CH, C2 Tyr), 128.7 (C, C3a Trp), 125.7 (C, C1 Tyr), 125.0 (CH, C2 Trp), 122.7 (CH, C6 Trp), 120.1 (CH, C5 Trp), 119.4 (CH, C4 Trp), 116.9 (2 x CH, C3 Tyr), 112.5 (CH, C7 Trp), 110.4 (C, C3 Trp), 61.6 (CH, Cα Pro), 56.3 (CH, Cα Trp), 54.7 (CH, Cα Tyr), 53.0 (CH, Cα TFM), 48.5 (CH_2, Cδ Pro), 36.9 (CH_2, Cβ Tyr), 33.4 (CH_2, Cβ TFM), 30.0 (CH_2, Cβ Pro), 28.3 (CH_2, Cβ Trp), 27.2 (CH_2, Cγ TFM), 26.0 (CH_2, Cγ Pro); **minor conformer (*cis*):** δ = 175.1 (C=O, $CONH_2$), 174.3 (C=O, Trp-TFM), 173.4 (C=O, Pro-Trp), 169.0 (C=O, Tyr-Pro), 158.6 (C, C4 Tyr), 138.1 (C, C7a Trp), 131.5 (2 x CH, C2 Tyr), 128.6 (C, C3a Trp), 125.4 (C, C1 Tyr), 124.8 (CH, C2 Trp), 122.8 (CH, C6 Trp), 120.2 (CH, C5 Trp), 119.3 (CH, C4 Trp), 116.9 (2 x CH, C3 Tyr), 112.6 (CH, C7 Trp), 110.7 (C, C3 Trp), 61.3 (CH, Cα Pro), 56.1 (CH, Cα Trp), 54.6 (CH, Cα Tyr), 53.1 (CH, Cα TFM), 48.2 (CH_2, Cδ Pro), 38.1 (CH_2, Cβ Tyr), 33.7 (CH_2, Cβ TFM), 32.6 (CH_2, Cβ Pro), 28.4 (CH_2, Cβ Trp), 27.2 (CH_2, Cγ TFM), 22.9 (CH_2, Cγ Pro).

SCF_3 quartet resonance of the minor conformer could not be observed.

^{19}F NMR (565 MHz, MeOD-d_4, 10 mM): δ = –42.72 (3F, SCF_3 major), –42.76 (3F, SCF_3 minor), –77.06 (3F, TFA).

HRMS (ESI) m/z: $[M + H]^+$ Calcd. for $C_{30}H_{36}F_3N_6O_5S^+$ 649.2415; Found: 649.2413.

TFA.H-Tyr-Pro-Trp-**Phe(4-CF_3)**-NH_2 (**L9**)

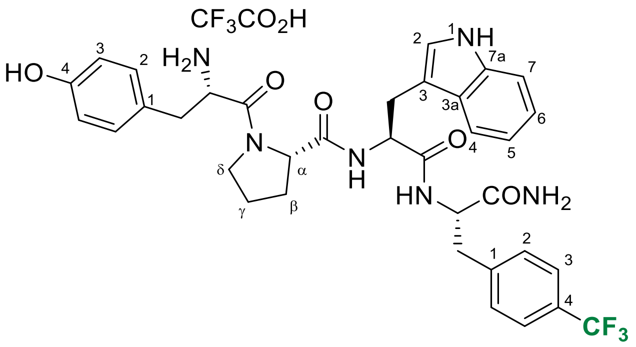

Synthesized following the *General SPPS procedure* using Rink amide AM resin (243 mg, 0.412 mmol/g, 0.10 mmol). The crude peptide was purified using preparative RP-HPLC (70% MQ H_2O + 0.1% TFA/30% MeCN + 0.1% TFA → 70% MeCN + 0.1% TFA) and freeze dried.

Yield: 35.2 mg (0.044 mmol, 44%) of white solid, as a mixture of two rotamers in a ratio 70:30 in MeOH at 298K.

^{1}H NMR (400 MHz, MeOH-*d₄*) **minor rotamer when visible* δ = 7.64 (d, J = 7.9 Hz, 1H), 7.60-7.50 (m, 2H), 7.44-7.6 (m, 1H), 7.36-7.28 (m, 2H), 7.20-7.03 (m, 4.4H), 6.94-6.89 (m, 0.7H), 6.81-6.76 (m, 1.4H), 6.75-6.72* (m, 0.6H), 4.72* (dd, J = 9.4, 5.9 Hz, 0.3H), 4.68-4.61 (m, 1H), 4.59 (dd, J = 7.9, 5.9 Hz, 0.7H), 4.46 (dd, J = 8.3, 4.9 Hz, 0.7H), 4.27 (dd, J = 7.5, 6.5 Hz, 0.7H), 3.62-3.48 (m, 1H), 3.40-3.30* (m, 0.3H), 3.30-2.87 (m, 7H), 2.85-2.77 (m, J = 14.0, 7.9 Hz), 2.07-1.98 (m, 0.7H), 1.92-1.74 (m, 2H), 1.65-1.54 (m, 0.7H), 1.54-1.42* (m, 0.3H), 1.33-1.19* (m, 0.3H).

^{19}F NMR (376 MHz, MeOD-*d₄*): δ = –63.87 (CF_3, s, 3F), –63.88* (CF,s, 3F), –77.02 (TFA, 3F).

HRMS (ESI) m/z: $[M + H]^+$ Calcd. for $C_{35}H_{38}F_3N_6O_5^+$ 679.2850; Found: 679.2834.

TFA.H-**Dmt**-Pro-Trp-**Phe(4-CF_3)**-NH_2 (**L10**)

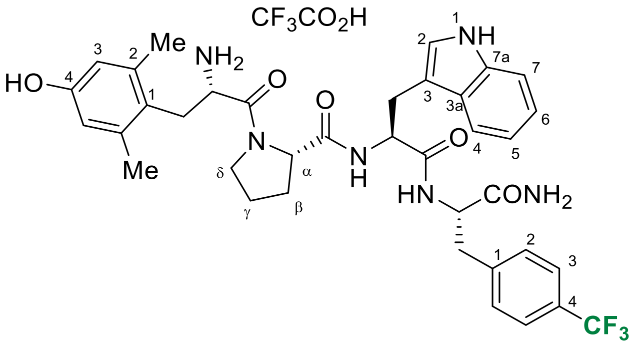

Synthesized following the *General SPPS procedure* using Rink amide AM resin (243 mg, 0.412 mmol/g, 0.1 mmol). The coupling with Fmoc-L-Dmt-OH **5b** was performed using DIC/Oxyma (1.5 equiv. each) for 3–4 hours. The crude peptide was purified using preparative

RP-HPLC (70% MQ H_2O + 0.1% TFA/30% MeCN + 0.1% TFA → 70% MeCN + 0.1% TFA) and freeze dried.

Yield: 28.8 mg (0.035 mmol, 35%) of white solid, as a mixture of two rotamers in a ratio 70:30 in MeOH at 298K.

^{1}H NMR (400 MHz, MeOH-*d4*) **minor rotamer when visible* δ = 7.64* (d, *J* = 7.9 Hz, 0.3H), 7.61 (d, *J* = 7.9 Hz, 0.7H), 7.56 (d, *J* = 8.1 Hz, 1.4H), 7.51* (d, *J* = 8.1 Hz, 0.6H), 7.41 (d, *J* = 8.1 Hz, 1.4H), 7.37* (d, *J* = 8.1 Hz, 0.6H), 7.32* (d, *J* = 7.6 Hz, 0.3H), 7.27 (d, *J* = 8.0 Hz, 0.7H), 7.17-7.00 (m, 3H), 6.54* (s, 0.6H), 6.48 (s, 1.4H), 4.67-4.60 (m, 1.4H), 4.60-4.52* (m, 0.6H), 4.45-4.36* (m, 0.3H), 4.27* (dd, *J* = 8.8, 7.1 Hz, 0.3H), 3.73 (dd, *J* = 12.0, 4.0 Hz, 0.7H), 3.46-3.35 (m, 0.7H), 3.29-2.87 (m, 8H), 2.25* (s, 1.8H), 2.10 (s, 4.2H), 1.81-1.67 (m, 1H), 1.60-1.39 (m, 2.3H), 1.22-1.07 (m, 0.7H).

^{19}F NMR (376 MHz, MeOD-*d4*): δ = –63.89 (CF_3, s, 3F), –77.07 (TFA, 3F).

HRMS (ESI) m/z: $[M + H]^+$ Calcd. for $C_{37}H_{42}F_3N_6O_5^+$ 707.3169; Found: 707.3141.

4. Chemical structure and RP-HPLC chromatograms of opioid ligands

a. TFA.H-Tyr-Pro-Trp-Phe-NH$_2$ (**EM1**)

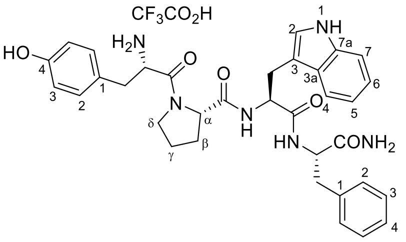

RP-HPLC analysis: >99% purity; t_R = 8.06 min (20% → 60% MeCN + 0.1% TFA in MQ H_2O + 0.1% TFA, λ = 210 nm, 20 min).

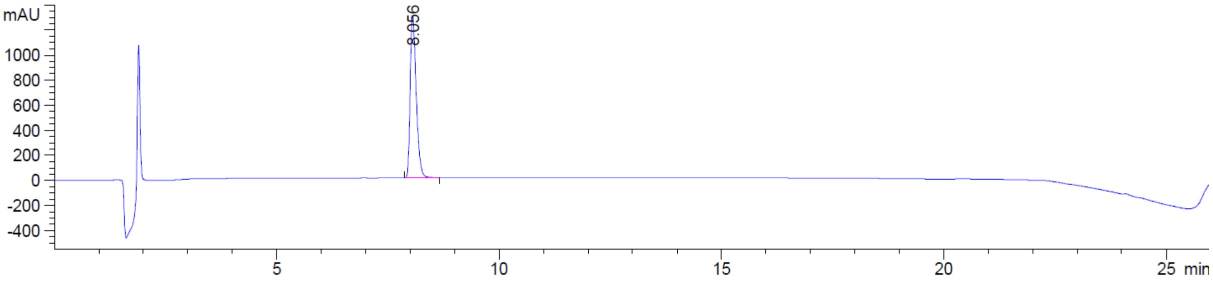

Figure S1: RP-HPLC chromatogram of EM1

b. TFA.H-**Dmt**-Pro-Trp-Phe-NH$_2$ (**L0**)

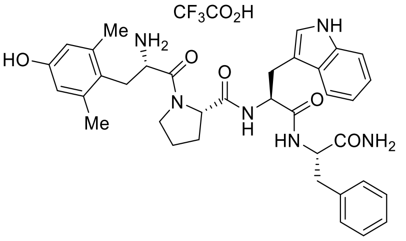

RP-HPLC analysis: >99% purity; t_R = 8.72 min (20% → 60% MeCN + 0.1% TFA in MQ H_2O + 0.1% TFA, λ = 210 nm, 20 min).

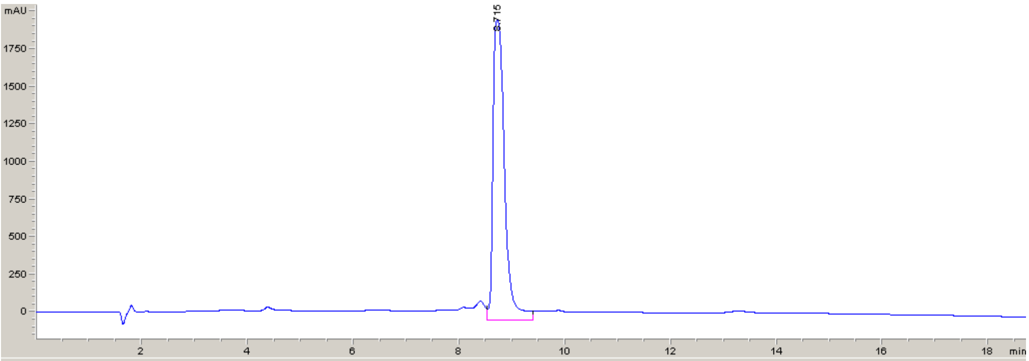

Figure S2: RP-HPLC chromatogram of **L0**

c. TFA.H-**Tyr(3-SCF₃)**-Pro-Trp-Phe-NH₂ (**L1**)

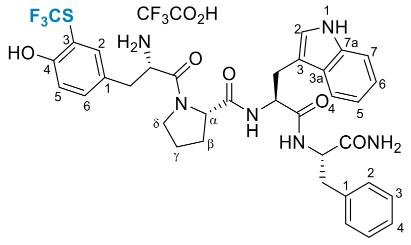

RP-HPLC analysis: >99% purity; t_R = 11.68 min (20% → 60% MeCN + 0.1% TFA in MQ H_2O + 0.1% TFA, λ = 210 nm, 20 min).

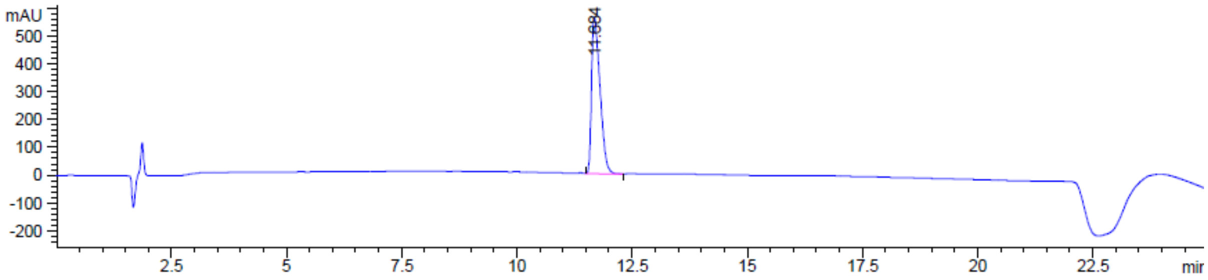

Figure S3: RP-HPLC chromatogram of **L1**

d. TFA.H-**Tyr(3-CF₃)**-Pro-Trp-Phe-NH₂ (**L2**)

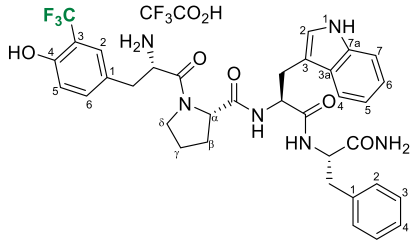

RP-HPLC analysis: >99% purity; t_R = 10.94 min (20% → 60% MeCN + 0.1% TFA in MQ H_2O + 0.1% TFA, λ = 210 nm, 20 min).

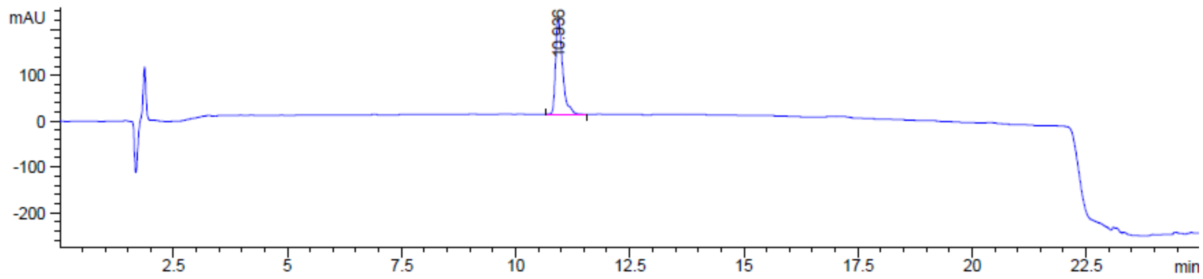

Figure S4: RP-HPLC chromatogram of **L2**

e. TFA.H-**Tyr(3,5-CF3)**-Pro-Trp-Phe-NH2 (**L3**)

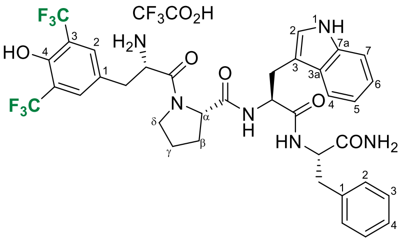

RP-HPLC analysis: 97% purity; t_R = 12.25 min (20% → 60% MeCN + 0.1% TFA in MQ H_2O + 0.1% TFA, λ = 210 nm, 20 min).

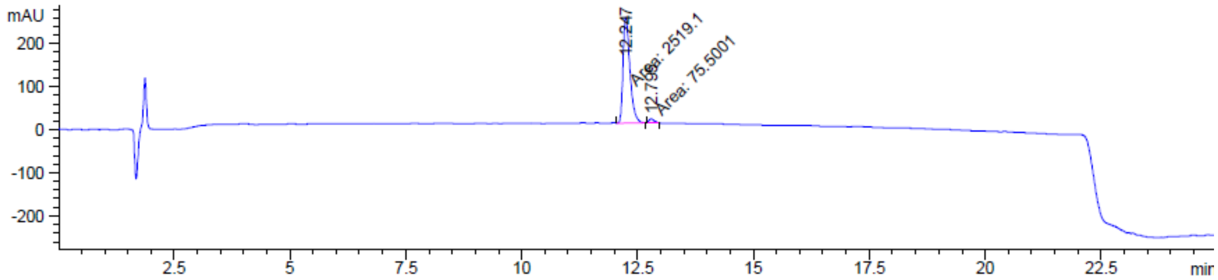

Figure S5: RP-HPLC chromatogram of **L3**

f. TFA.H-**Dmt(3-SCF3)**-Pro-Trp-Phe-NH2 (**L4**)

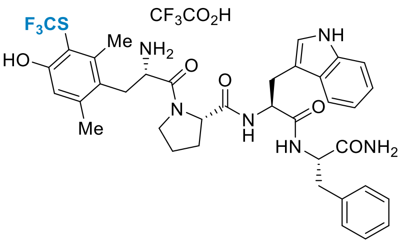

RP-HPLC analysis: 98% purity; t_R = 12.43–12.87 min (20% → 60% MeCN + 0.1% TFA in MQ H_2O + 0.1% TFA, λ = 210 nm, 20 min).

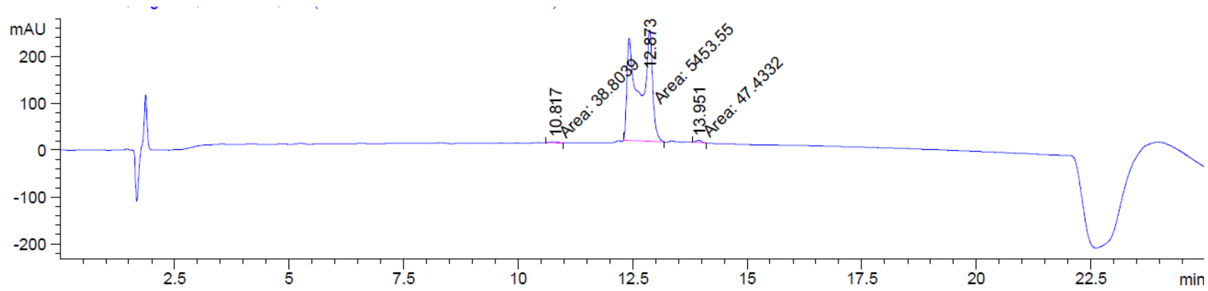

Figure S6: RP-HPLC chromatogram of **L4**

The eluent of the first major peak was collected and reinjected. It exhibited a similar distribution, indicating that the two major peaks are resolved rotamers.

g. TFA.H-Tyr-**ΨPro(CF$_3$)**-Trp-Phe-NH$_2$ (**L5**)

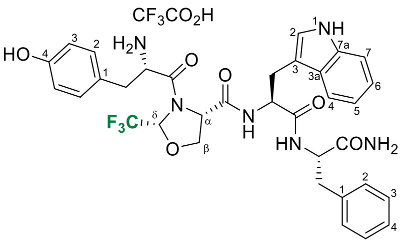

RP-HPLC analysis: 97% purity; t_R = 8.48 min (20% → 60% MeCN + 0.1% TFA in MQ H_2O + 0.1% TFA, λ = 210 nm, 20 min).

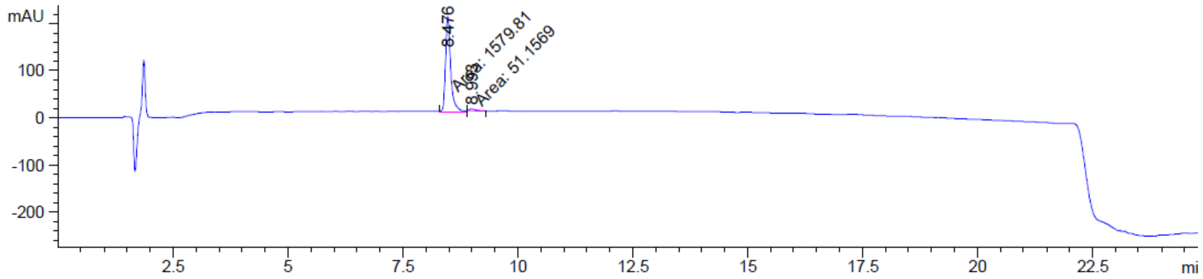

Figure S7: RP-HPLC chromatogram of **L5**

h. TFA.H-Tyr-Pro-**Trp(2-SCF$_3$)**-Phe-NH$_2$ (**L6**)

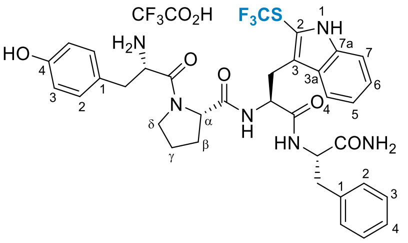

RP-HPLC analysis: >99% purity; t_R = 11.07 min (20% → 60% MeCN + 0.1% TFA in MQ H_2O + 0.1% TFA, λ = 210 nm, 20 min).

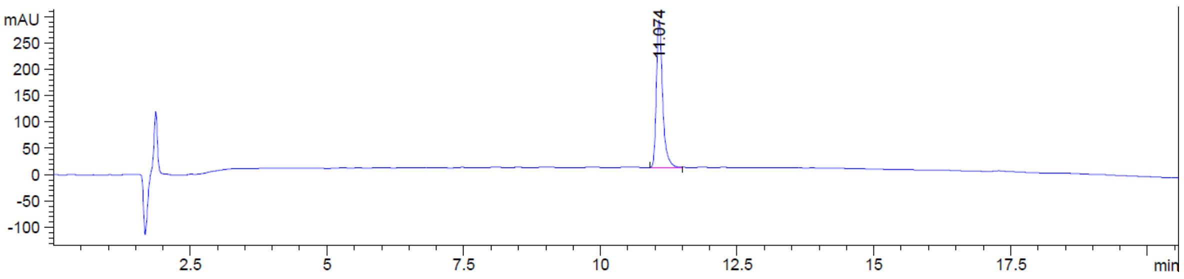

Figure S8: RP-HPLC chromatogram of **L6**

i. TFA.H-Tyr-Pro-**Trp(2-CF$_3$)**-Phe-NH$_2$ (**L7**)

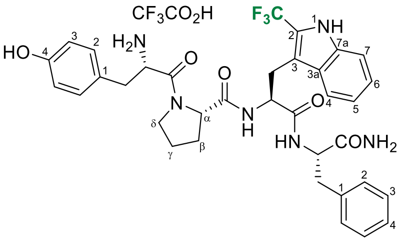

RP-HPLC analysis: >99% purity; t_R = 9.62 min (20% → 60% MeCN + 0.1% TFA in MQ H_2O + 0.1% TFA, λ = 210 nm, 20 min).

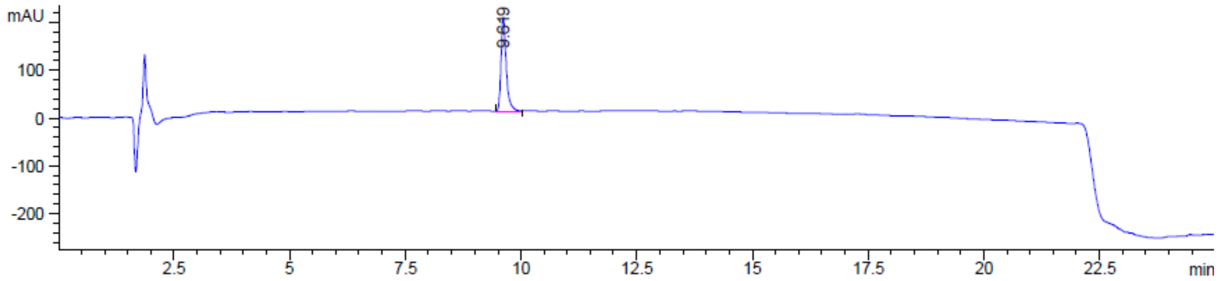

Figure S9: RP-HPLC chromatogram of **L7**

j. TFA.H-Tyr-Pro-Trp-**TFM**-NH$_2$ (**L8**)

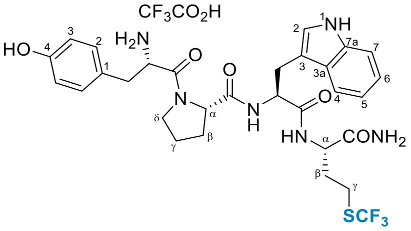

RP-HPLC analysis: >99% purity; t_R = 9.00 min (20% → 60% MeCN + 0.1% TFA in MQ H_2O + 0.1% TFA, λ = 210 nm, 20 min).

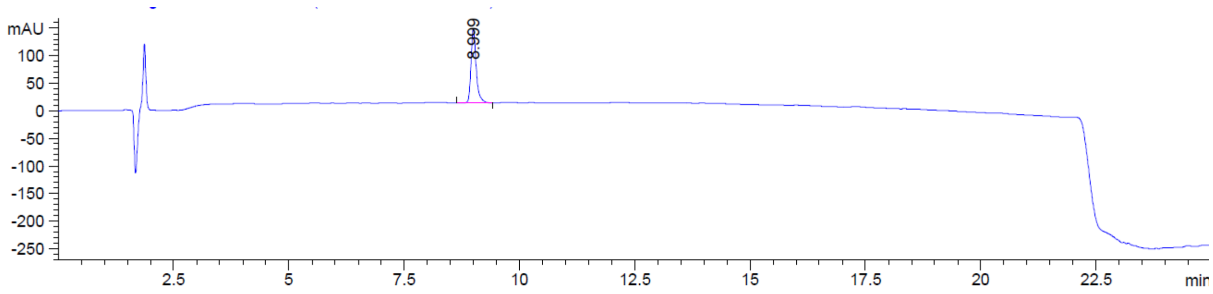

Figure S10: RP-HPLC chromatogram of **L8**

k. TFA.H-Tyr-Pro-Trp-**Phe(4-CF$_3$)**-NH$_2$ (**L9**)

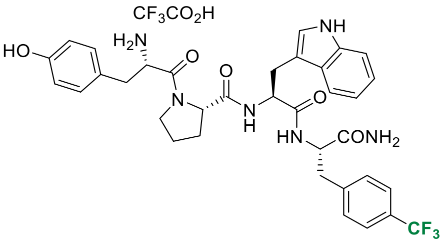

RP-HPLC analysis: 97% purity; t_R = 11.13 min (20% → 60% MeCN + 0.1% TFA in MQ H_2O + 0.1% TFA, λ = 210 nm, 20 min).

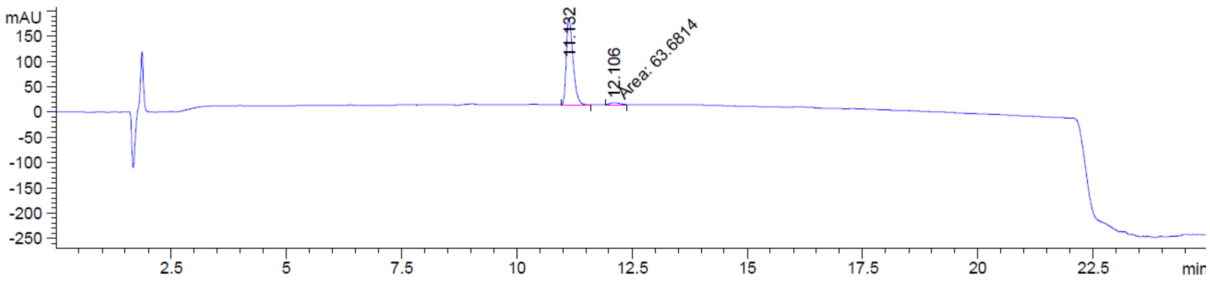

Figure S11: RP-HPLC chromatogram of **L9**

l. TFA.H-**Dmt**-Pro-Trp-**Phe(4-CF$_3$)**-NH$_2$ (**L10**)

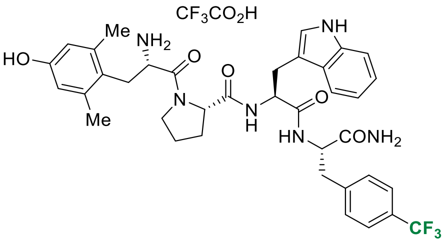

RP-HPLC analysis: 96% purity; t_R = 11.90–12.13 min (20% → 60% MeCN + 0.1% TFA in MQ H_2O + 0.1% TFA, λ = 210 nm, 20 min).

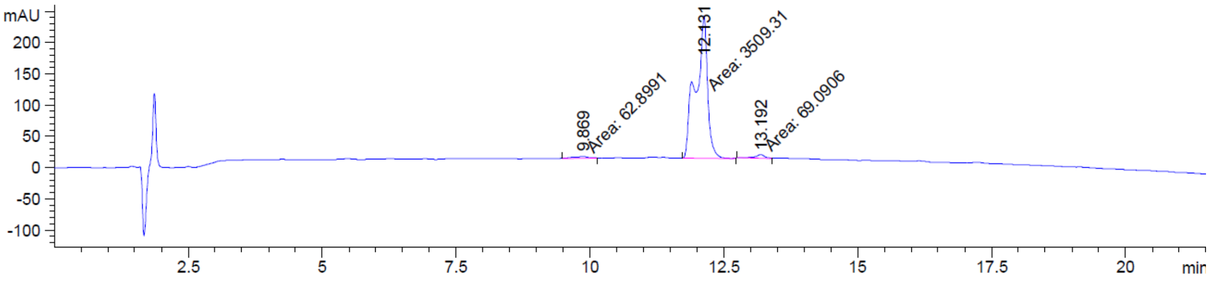

Figure S12: RP-HPLC chromatogram of **L10**

5. Biological activity assays

Materials

HEK293 (human embryonic kidney) cells stably expressing the mouse 3xHA-MOP were grown at 37 °C in DMEM supplemented with 10% fetal bovine serum and 10 IU penicillin, 100 µg/mL streptomycin and 2 mM glutamine. Plasmid expression was retained by growing the cells in geneticin (250 mg/L)-containing medium. Cells were maintained in a humidified atmosphere of 95% air and 5% CO_2.

Binding on MOP

We evaluated the binding affinity (IC_{50}) of the different compounds for MOP using membrane extracts from HEK293 cells. Briefly, HEK293 cells stably expressing MOP grown to confluence in 150 mm Petri dishes were frozen at –80°C until use. On the day of the experiment, cells were submitted to heat shock by placing the Petri dishes at 37 °C for 60 seconds before returning to ice. Cells were then harvested in ice-cold buffer A (Tris-HCl 20 mM, $MgCl_2$ 5 mM, NaCl 150 mM, pH 7.4) using a cell scraper and centrifuged at 3200 g for 15 minutes at 4 °C. The pellet containing the membrane extract was resuspended in 1 mL of buffer A. The protein concentration was determined with Bio-Rad DC™ Protein Assay reagents (Bio-Rad Laboratories, Mississauga, Ontario, Canada) and the pellet was further diluted in buffer A containing 0.1% BSA and 0.01% bacitracin and distributed in 96-well plate. [^{125}I]-DAMGO was used to determine the binding affinity of the compounds in a competitive binding assay. Experiments were performed using 15–20 µg of membrane proteins and 50000 cpm of the radiolabeled ligand. Non-specific binding was determined using 10 µM of non-radioactive DAMGO. Incubations were performed for 60 minutes at ambient temperature and the reaction was stopped by filtration using ice-cold buffer A on filtered 96-well plates. Filters were placed in 5 mL tubes and the radioactivity was determined using a Wizard Automatic Gamma Counter (PerkinElmer Canada). Data were analyzed using a non-linear fitting analysis, and the IC_{50} values were determined using GraphPad Prism 10 (GraphPad Software, San Diego, California, USA). IC_{50} were expressed as the mean ± S.E.M. from four independent experiments.

Inhibition of cAMP production

HEK293 cells were maintained in Dulbeccos-modified Eagles medium supplemented with 10% fetal bovine serum, penicillin (100 IU/mL) and streptomycin (100 µg/mL) at 37 °C in a humidified 5% CO_2 atmosphere. 500 ng of the indicated receptor and 100 ng of RLuc2-EPAC-GFP10 per microgram of total transfected DNA were prepared in 150 mM NaCl. Salmon sperm DNA was used to bring total transfected DNA to 1 µg, and each mixture was incubated for 20 minutes with 3 µg of polyethylenimine (Polysciences, Warrington, Pennsylvania, USA) per microgram of total transfected DNA before adding cells (350×10^3 cells/mL). Cells were plated at 35×10^3 cells per well in 96-well, flat-bottom, white opaque tissue culture plates. 48 hours

after seeding, cells were gently washed with stimulation buffer (10 mM Hepes, 1 mM $CaCl_2$, 0.5 mM $MgCl_2$, 4.2 mM KCl, 146 mM NaCl and 5.5 mM glucose), and 80 µl of stimulation buffer was added to each well. Coelenterazine 400A (Gold Biotechnology Inc., St. Louis, Misuri, USA) was added to a final concentration of 5 µM, 10 minutes before stimulation. For the EPAC (exchange protein directly activated by cAMP) assay, cells were stimulated with increasing concentrations of indicated ligand containing 3 µM forskolin (to increase cAMP) (Tocris Bioscience, Oakville, Ontario, Canada) for 10 minutes before signal acquisition. BRET2 (bioluminescence resonance energy transfer 2) signals were measured using a Berthold TriStar2 LB 942 Multimode Reader (Berthold, Bad Wildbad, Germany) RLuc2 and GFP10 emissions were collected in the 400-450 nm window (RLuc2) and 500-550 nm window (GFP10). The BRET2 signal was calculated as the ratio of light emitted by the acceptor GFP10 over the light emitted by the donor RLuc2. Data were normalized as percentage of the maximal response, where 100% was the maximal effect measure for DAMGO on µOR. All data were analyzed using the nonlinear curve fitting equations in GraphPad Prism 10 to estimate the EC_{50} values of the curves for the different pathways. Results are expressed as means ± S.E.M. from n = 3 independent experiments, each performed in duplicate.

6. Plasma stability assay

Human plasma was obtained from the Belgian Red Cross (Vlaams-Brabant, Leuven) and stored at –20°C. First the selectivity of the method and the stability of the peptide in the injection solvent were evaluated. Lyophilized peptides were dissolved in milliQ water (2 mM stock solution) and consecutive dilutions were prepared. Three independent calibration curves were constructed, and the linearity and precision of the method was determined. Once the method was validated, the stability experiments were performed in triplicate. Frozen human plasma samples were thawed and thermostated to 37 °C in an incubator for at least 30 minutes. Aqueous peptide solutions (1120 µM) were spiked in human plasma (10:90 v/v peptide solution/plasma) resulting in a final peptide concentration of 14 µM after precipitation. During the plasma stability study, samples were taken after 0, 1, 3, 4, 5, 6, 8, 10, 12.5 and 15 min for peptide **EM1**; 0, 9, 18, 27, 36, 45, 54, 63 and 72 min for peptide **L0**; 0, 90, 180, 270, 360, 450, 540, 630, 780, 870, 960, 1050, 1140, 1230 and 1440 min for peptide **L4** and 0, 20, 40, 60, 80, 100, 120, 140, 160, 180 and 200 min for peptide **L10**.

At every time point, 100 µL of spiked plasma was taken and 300 µL of cold (4 °C) precipitation solvent (methanol containing 0.1% TFA v/v) was added, allowing a protein crash. The resulting suspensions were vortexed for 15 seconds and placed at 4 °C for 30 minutes. After centrifugation for 15 minutes at 18625 g, 100 µL supernatant was diluted with 100 µL water in the injection vial. Peptide half-life was calculated based on the points with an area under the curve (AUC) higher than the AUC of the lowest standard concentration. Concentrations were calculated by use of the calibration curve and transferred to a semi-log chart presenting the log concentrations as a function of time. The optimum curve was used to calculate the peptide half-life. Data analysis was performed using Microsoft® Office 365 Excel.

7. Copies of NMR spectra

Starting substrates and reagents

Figure S13: ^{1}H NMR spectrum of **1** in $CDCl_3$, 500 MHz

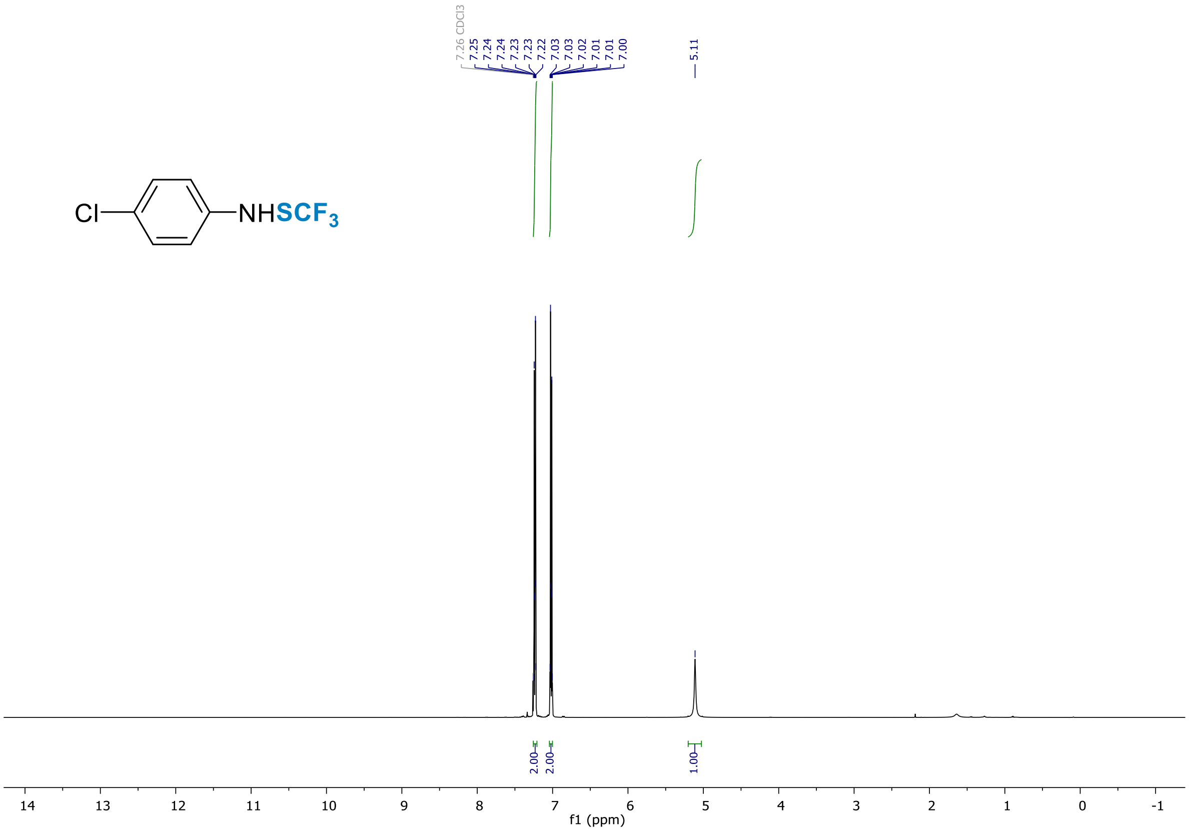

Figure S14: ^{19}F NMR spectrum of **1** in $CDCl_3$, 471 MHz

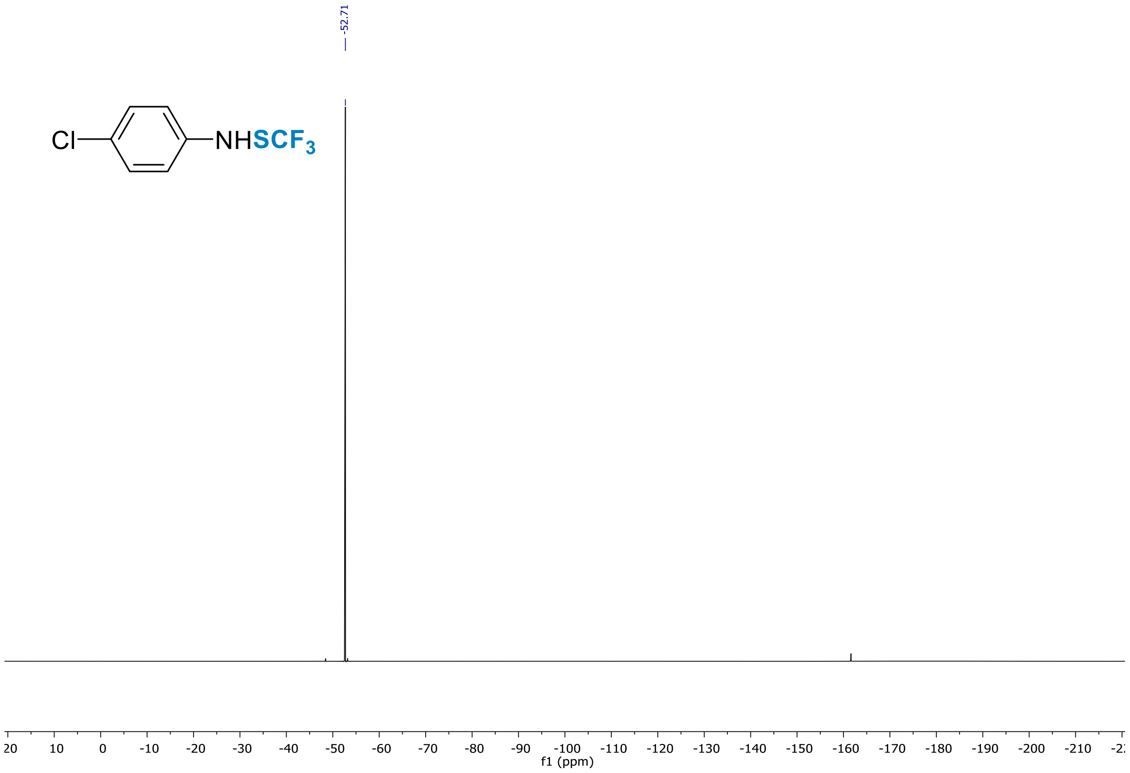

Figure S15: ^{1}H NMR spectrum of **2b** in $CDCl_3$, 600 MHz

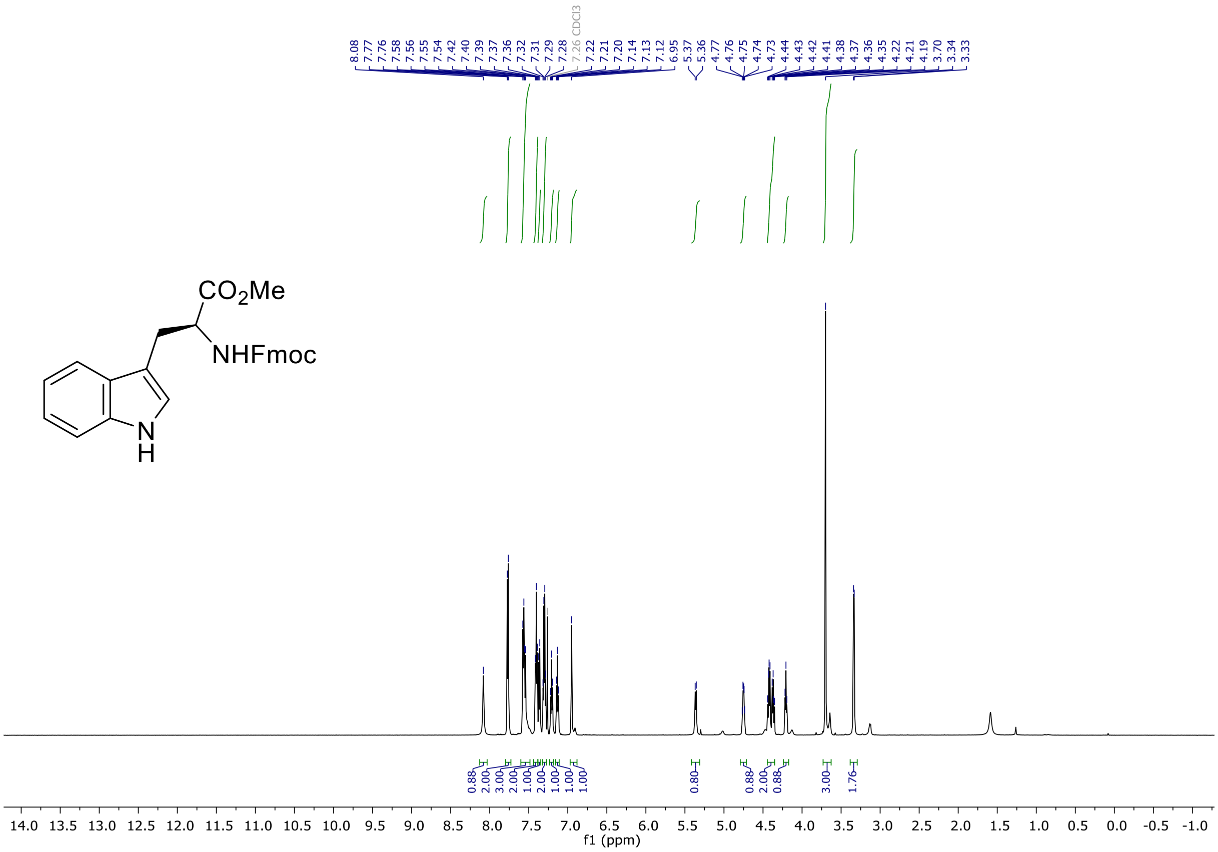

Figure S16: ^{1}H NMR spectrum of **4c** in $CDCl_3$, 400 MHz

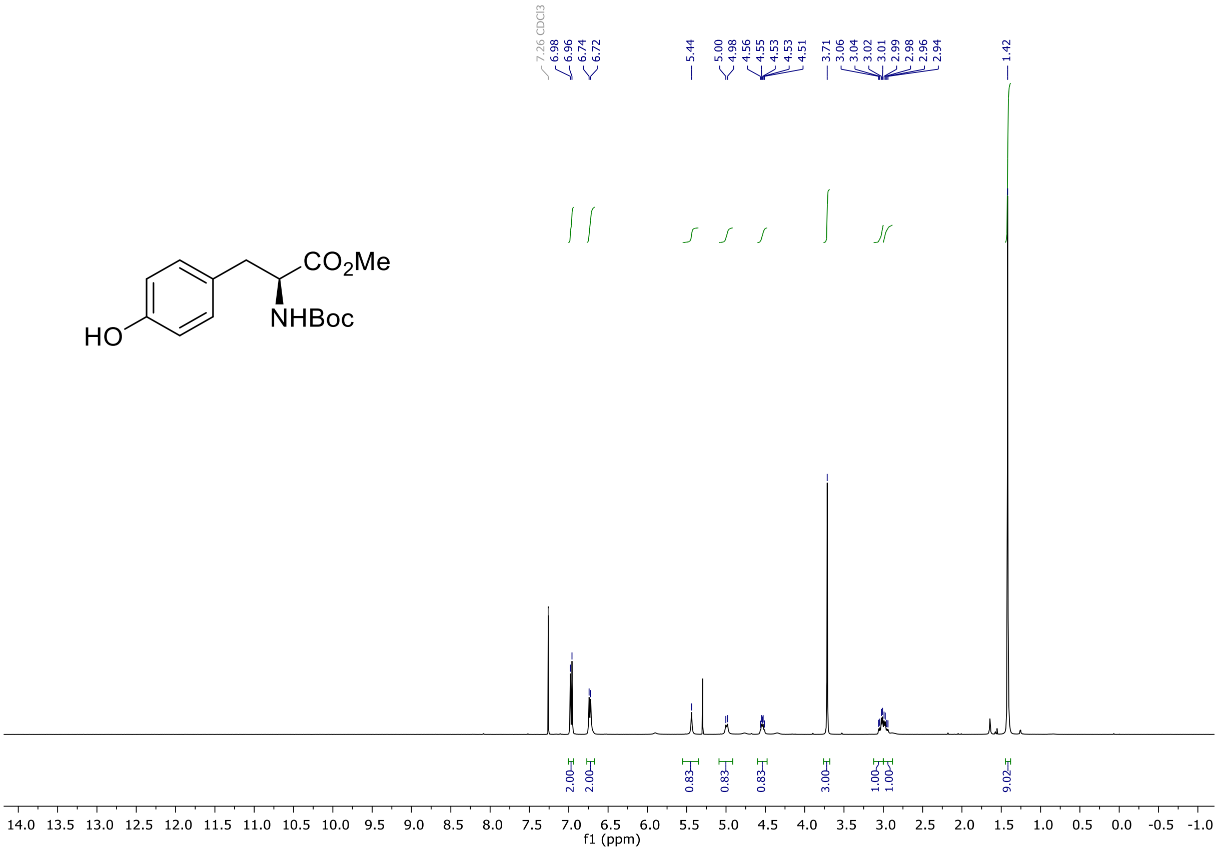

Fluorinated building blocks

Figure S17: ^{1}H NMR spectrum of **3a** in DMSO-d_6, 600 MHz

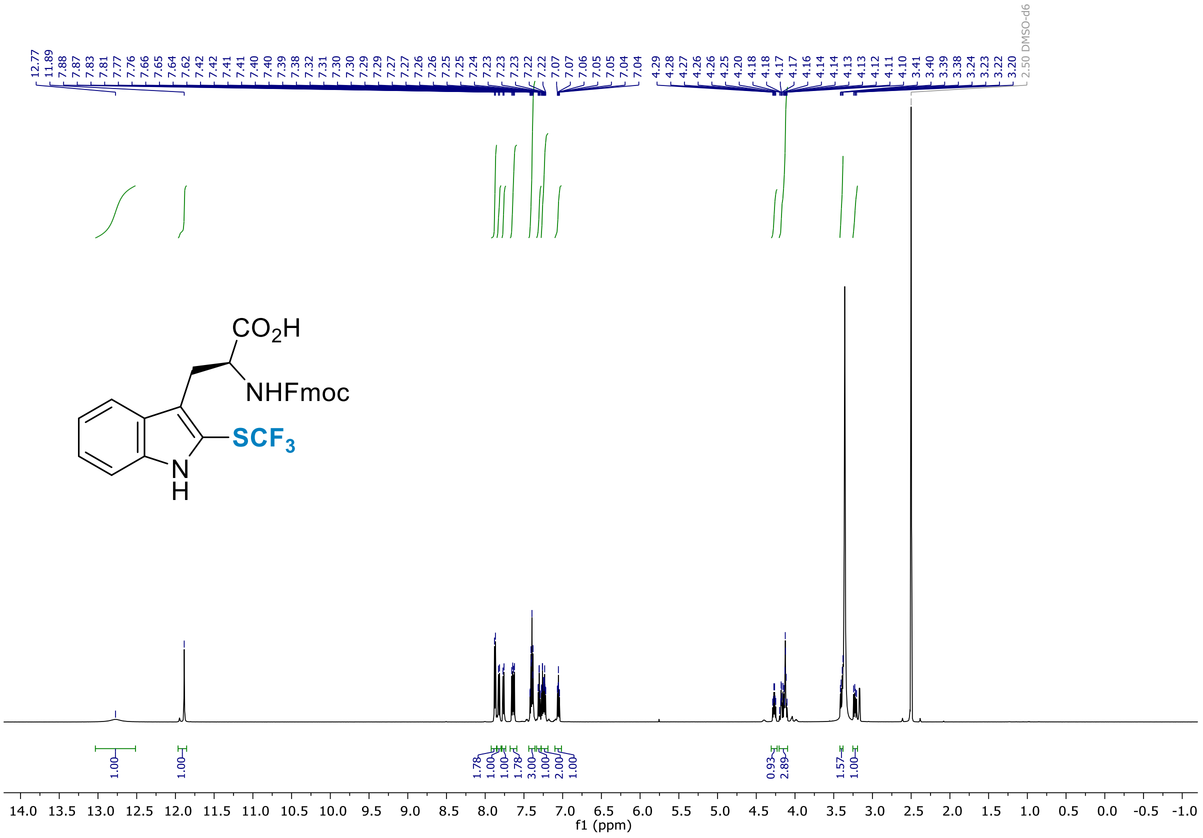

Figure S18: ^{19}F NMR spectrum of **3a** in DMSO-d_6, 565 MHz

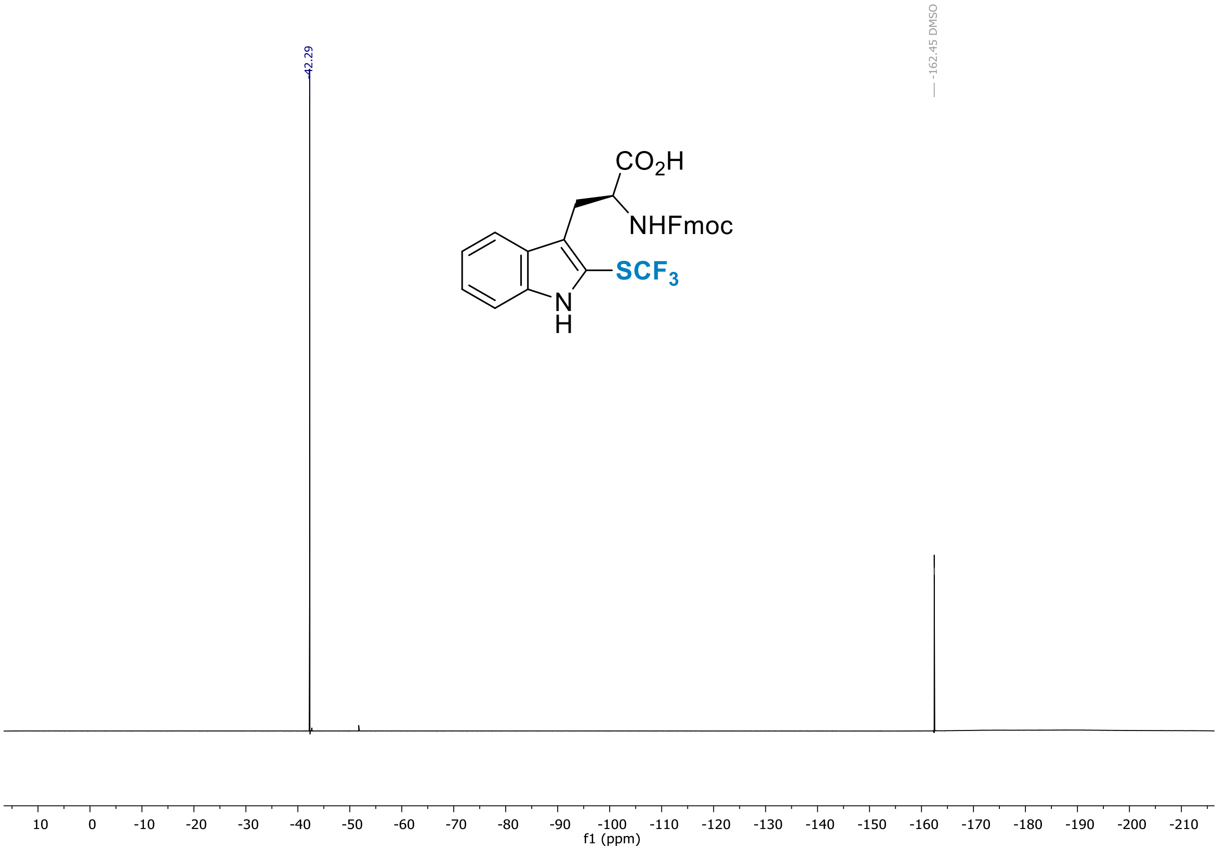

Figure S19: ^{1}H NMR spectrum of **5a** in DMSO-d_6, 600 MHz

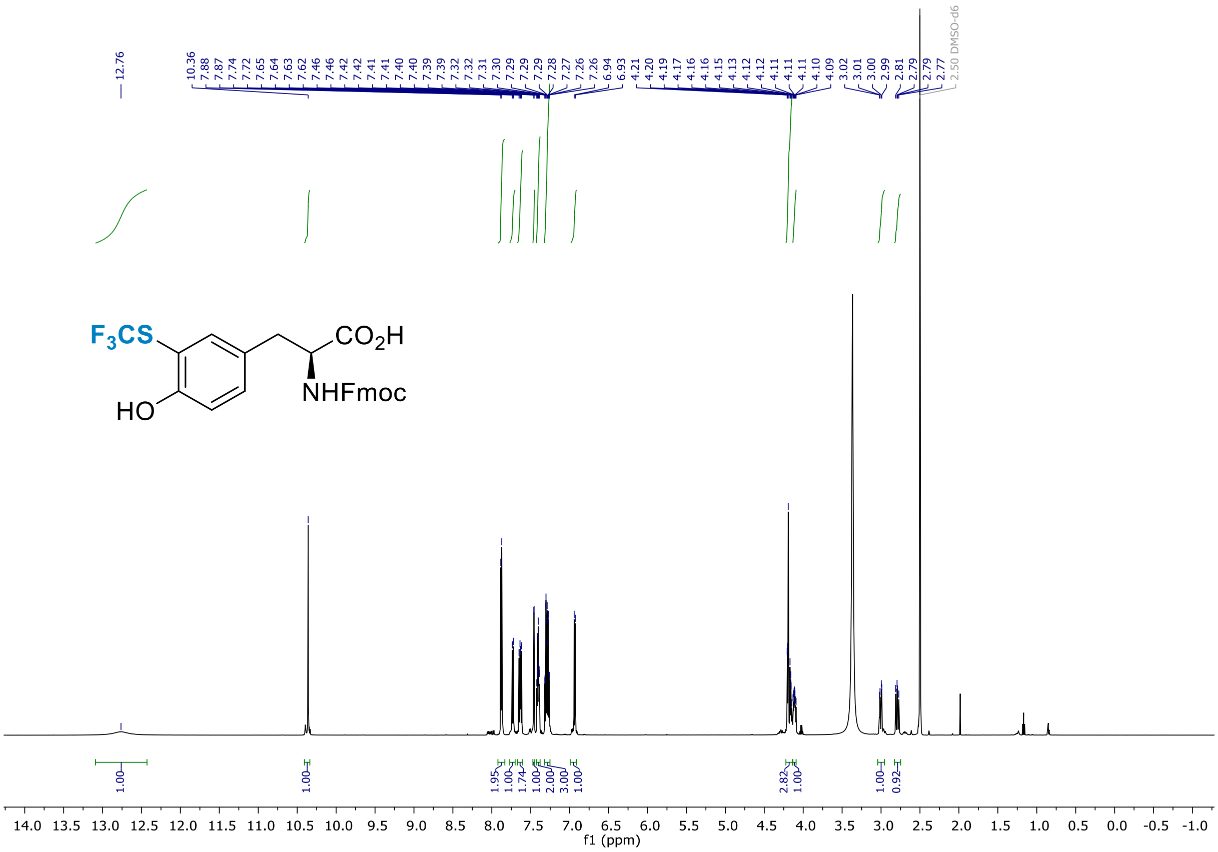

Figure S20: ^{19}F NMR spectrum of **5a** in DMSO-d_6, 565 MHz

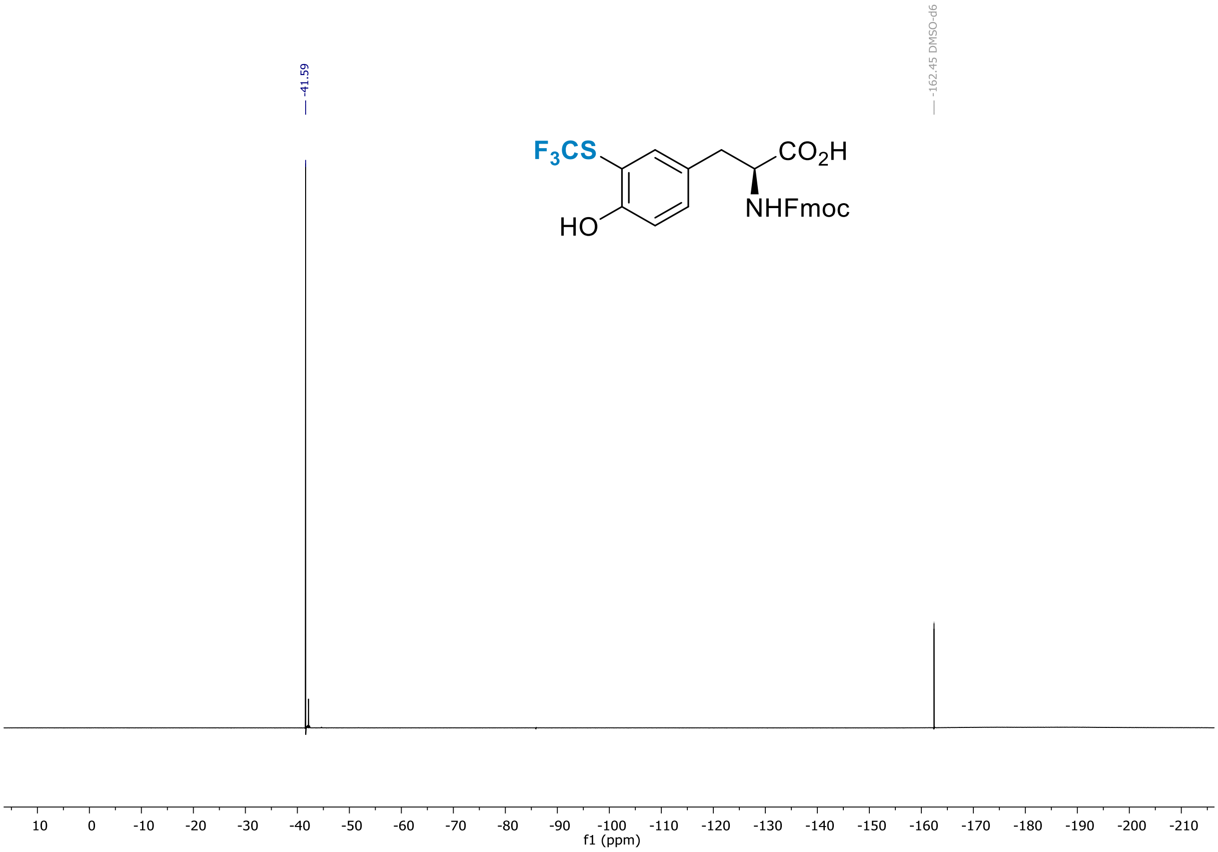

Figure S21:^{1}H NMR spectrum of **5b** in MeOD-*d₄*, 600 MHz

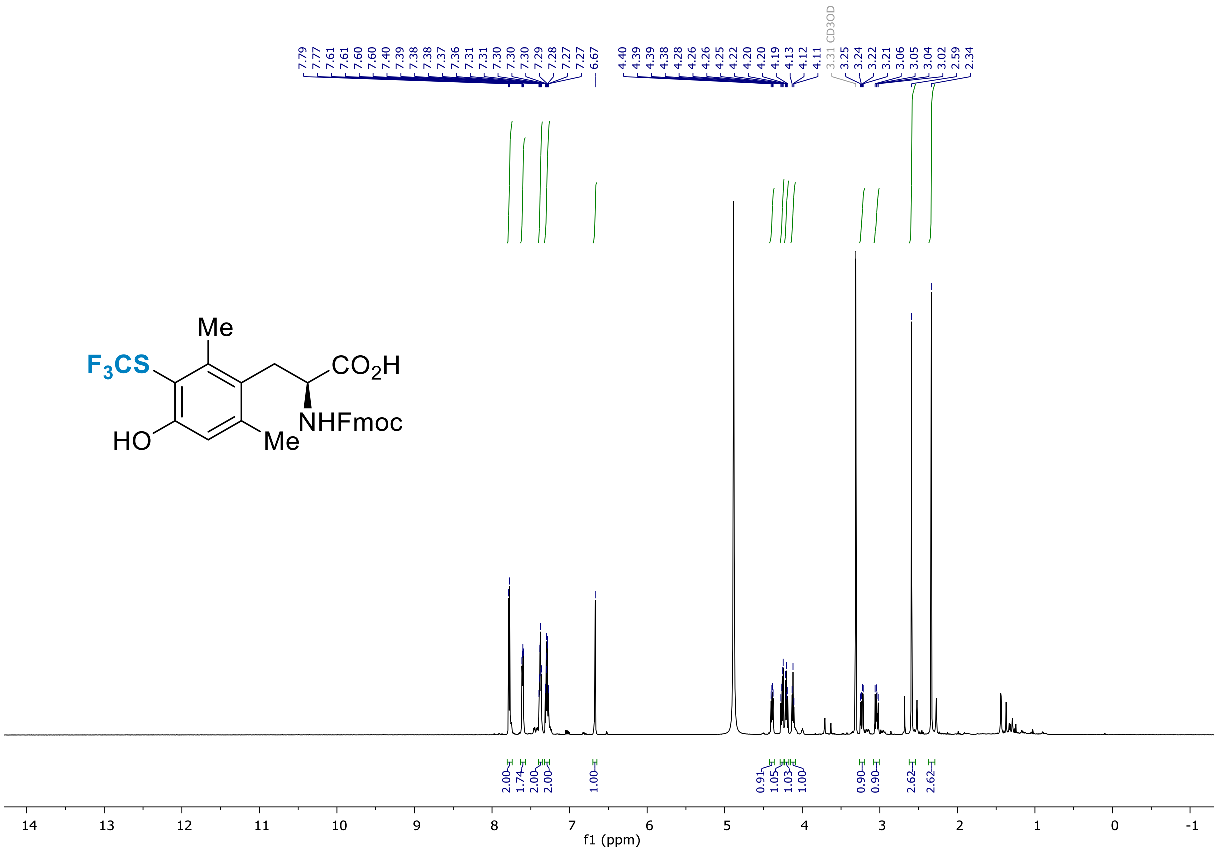

Figure S22: ^{13}C{^{1}H} NMR spectrum of **5b** in MeOD-*d₄*, 101 MHz

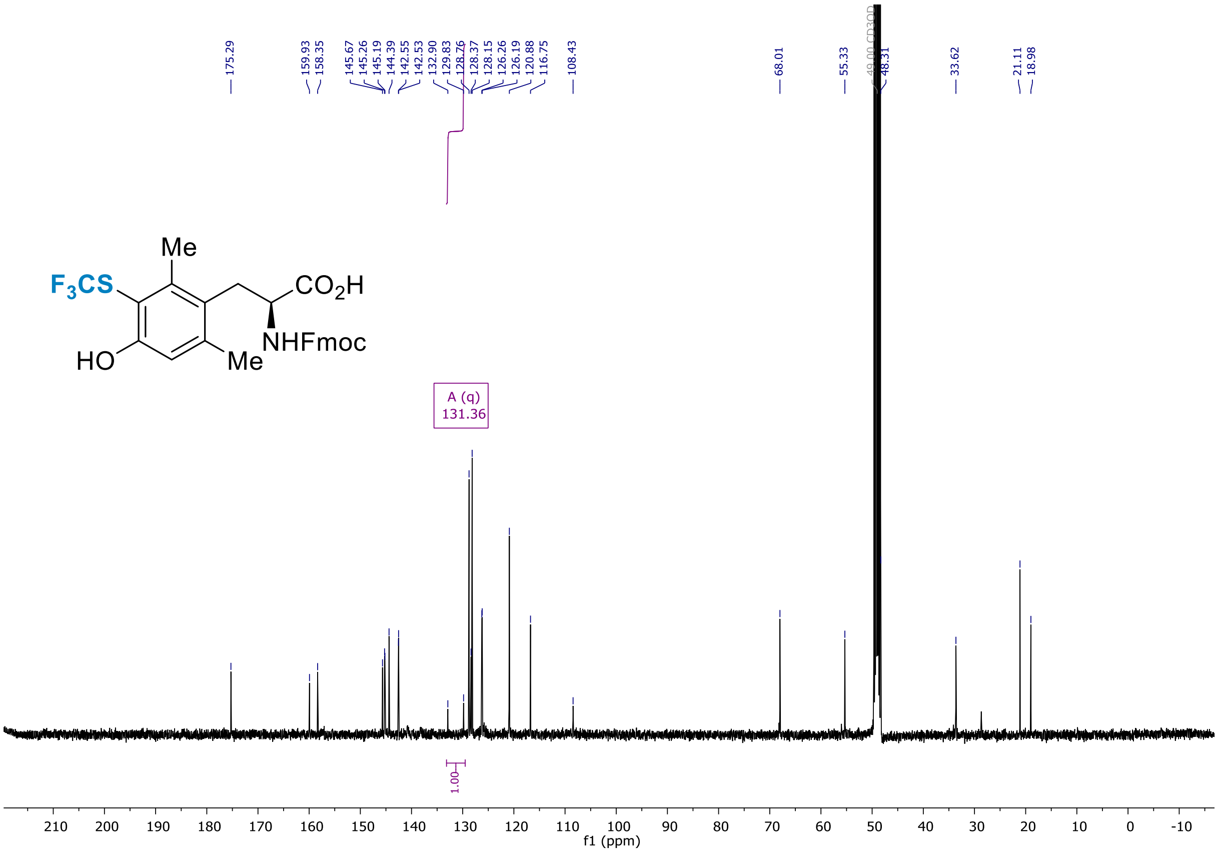

Figure S23: ^{19}F NMR spectrum of **5b** in MeOD-d_4, 565 MHz

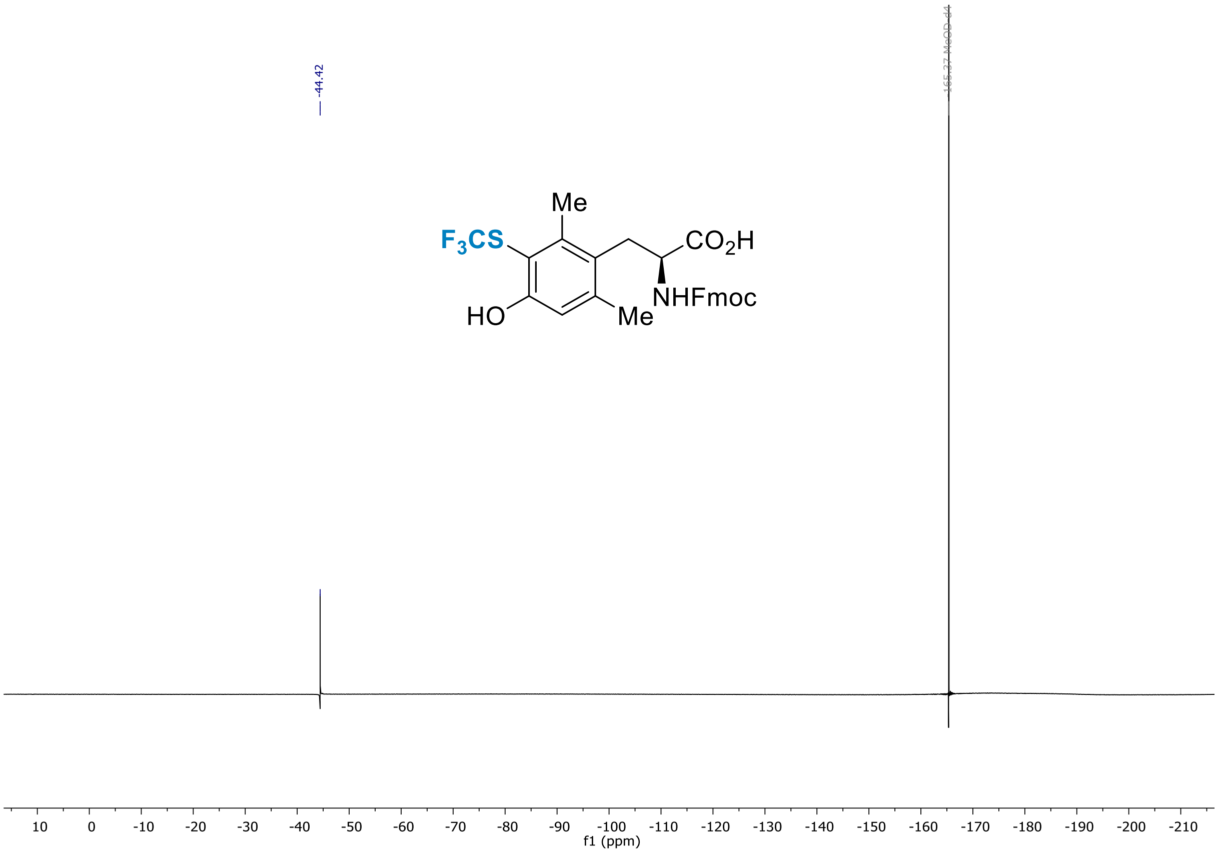

Figure S24: ^{1}H NMR spectrum of **6a** in $CDCl_3$, 600 MHz

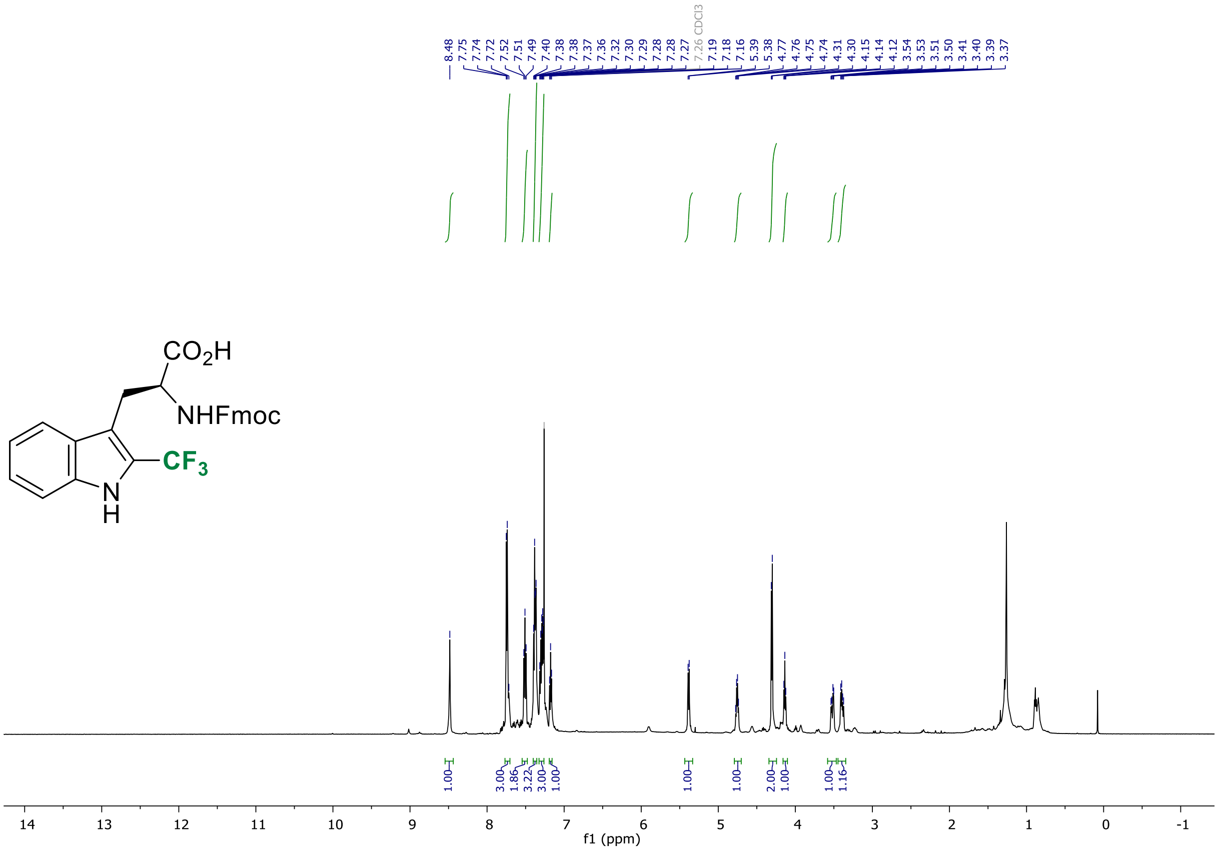

Figure S25: $^{13}C\{^{1}H\}$ spectrum of **6a** in $CDCl_3$, 151 MHz

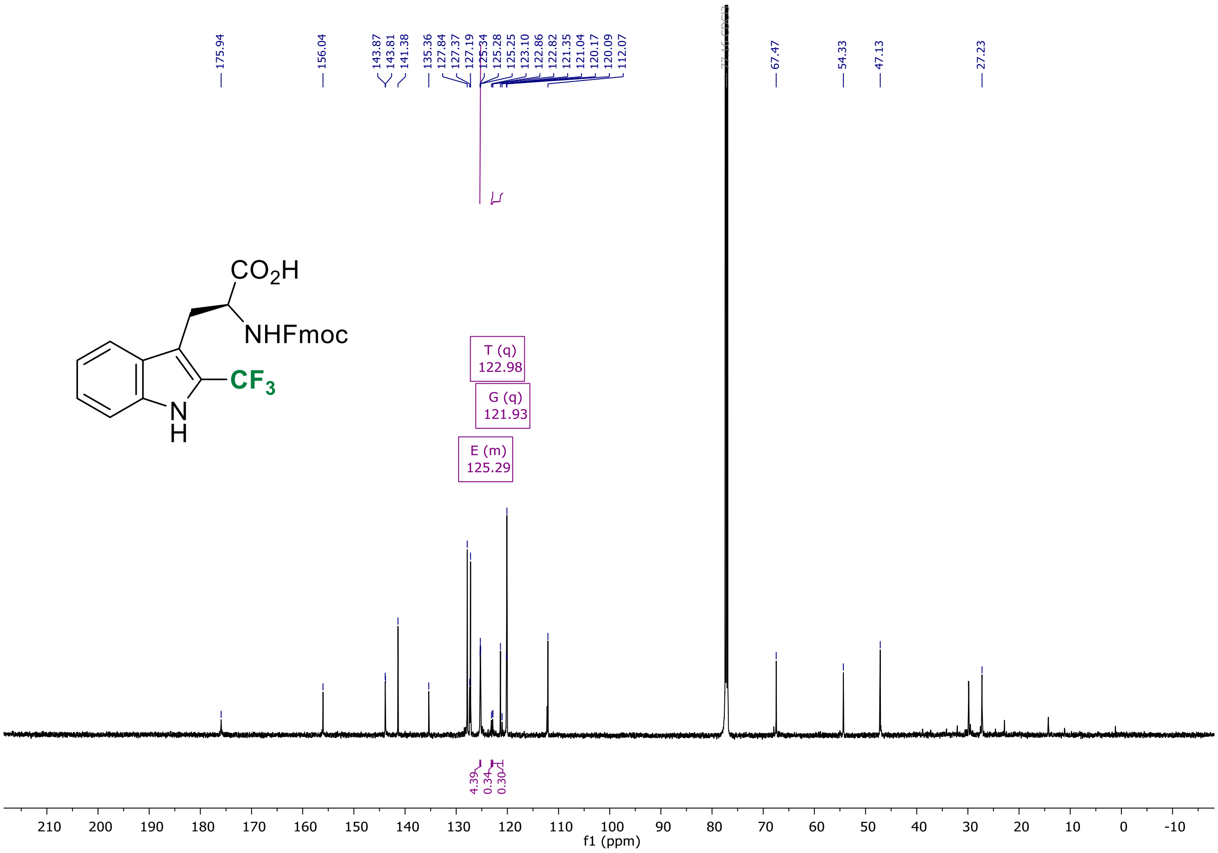

Figure S26: ^{19}F NMR spectrum of **6a** in $CDCl_3$, 565 MHz

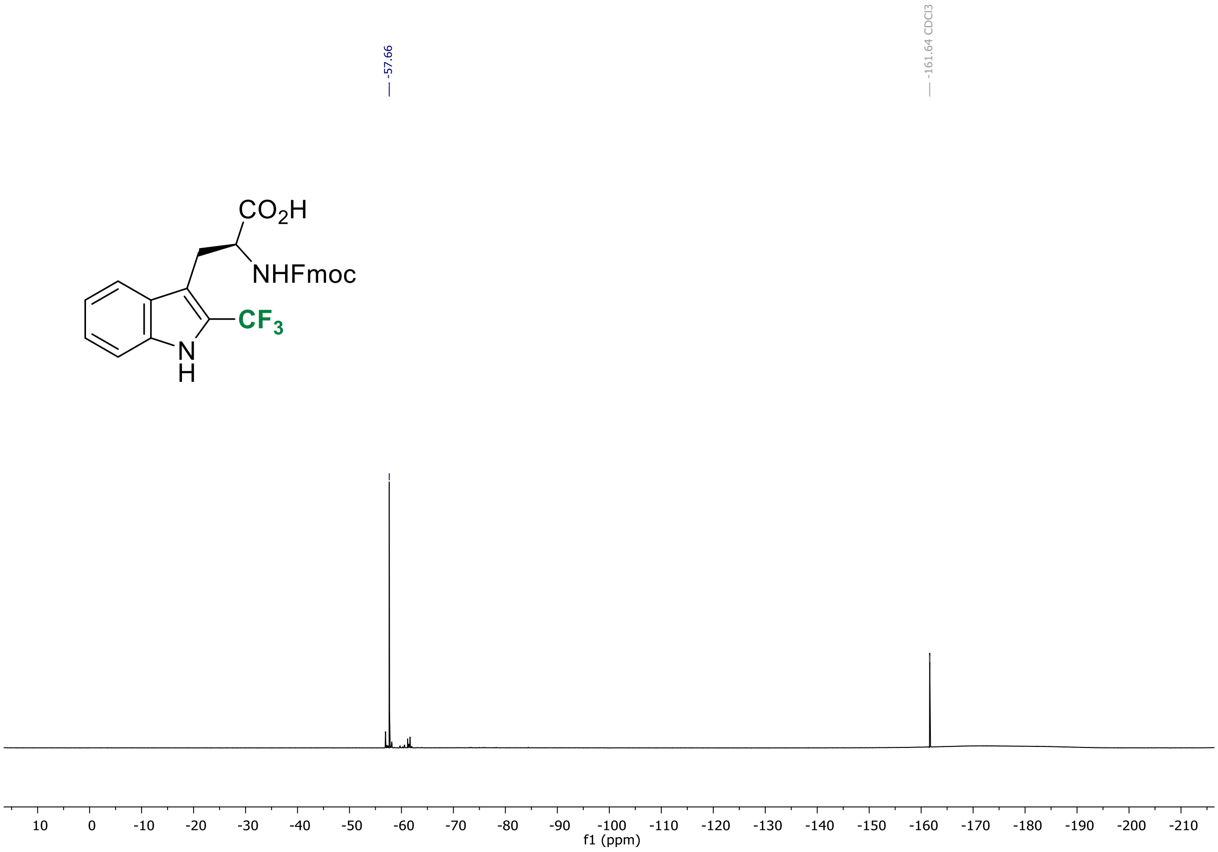

Figure S27: ^{1}H NMR spectrum of **6b** in $CDCl_3$, 600 MHz

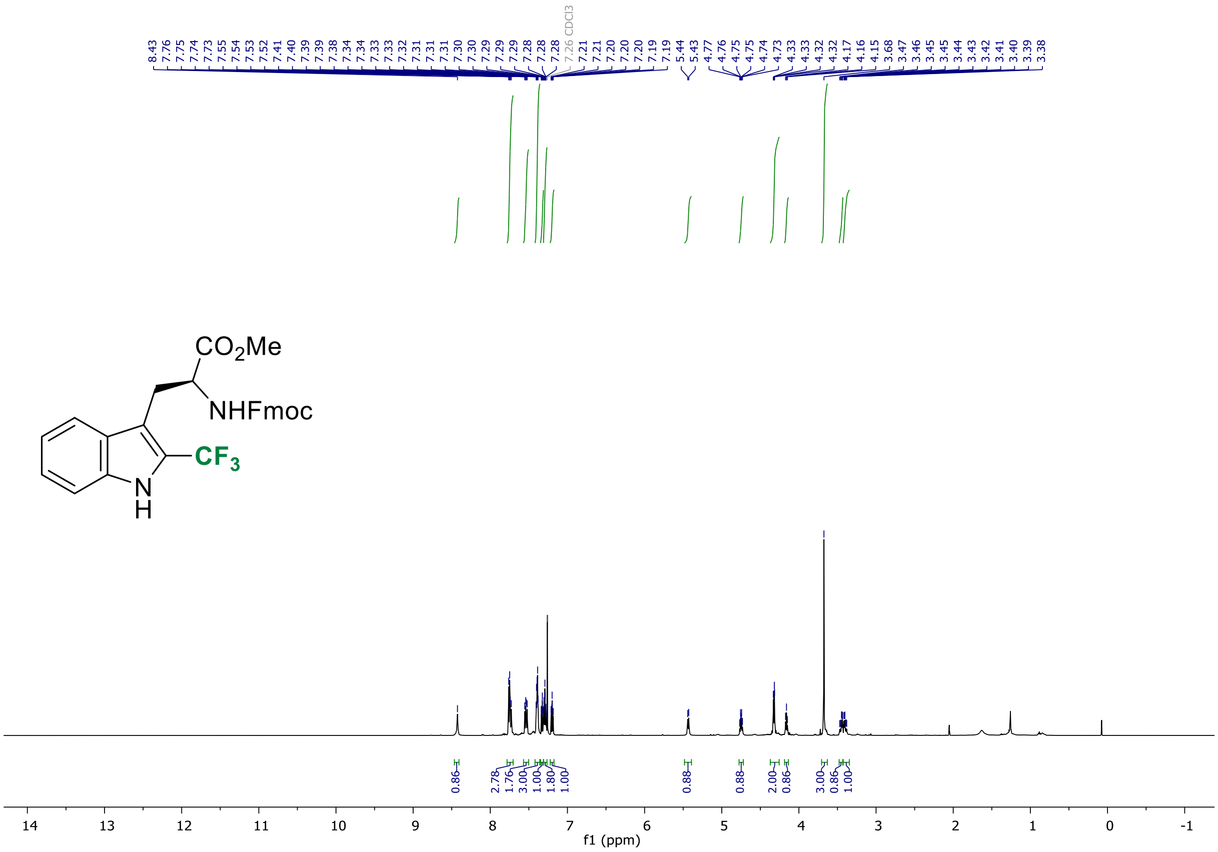

Figure S28: ^{13}C{^{1}H} spectrum of **6b** in $CDCl_3$, 151 MHz

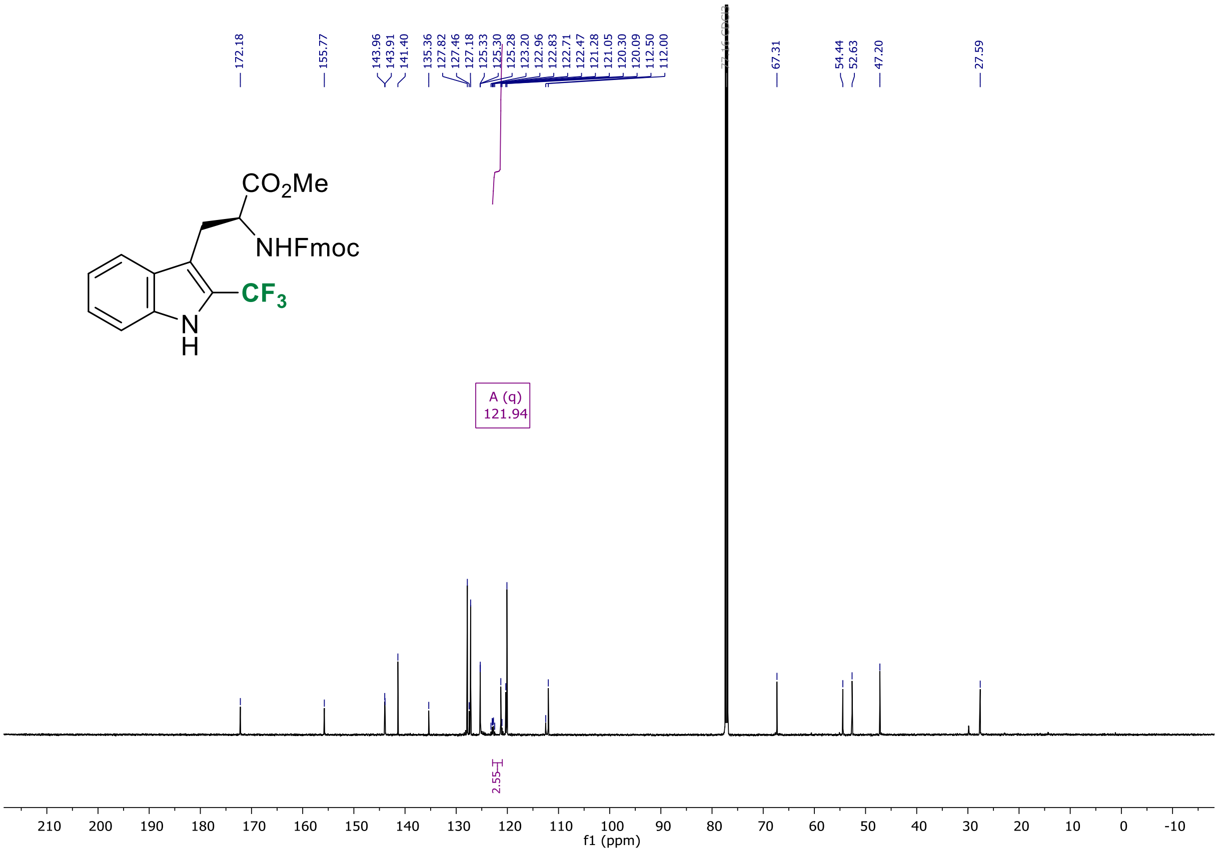

Figure S29: ^{19}F NMR spectrum of **6b** in $CDCl_3$, 565 MHz

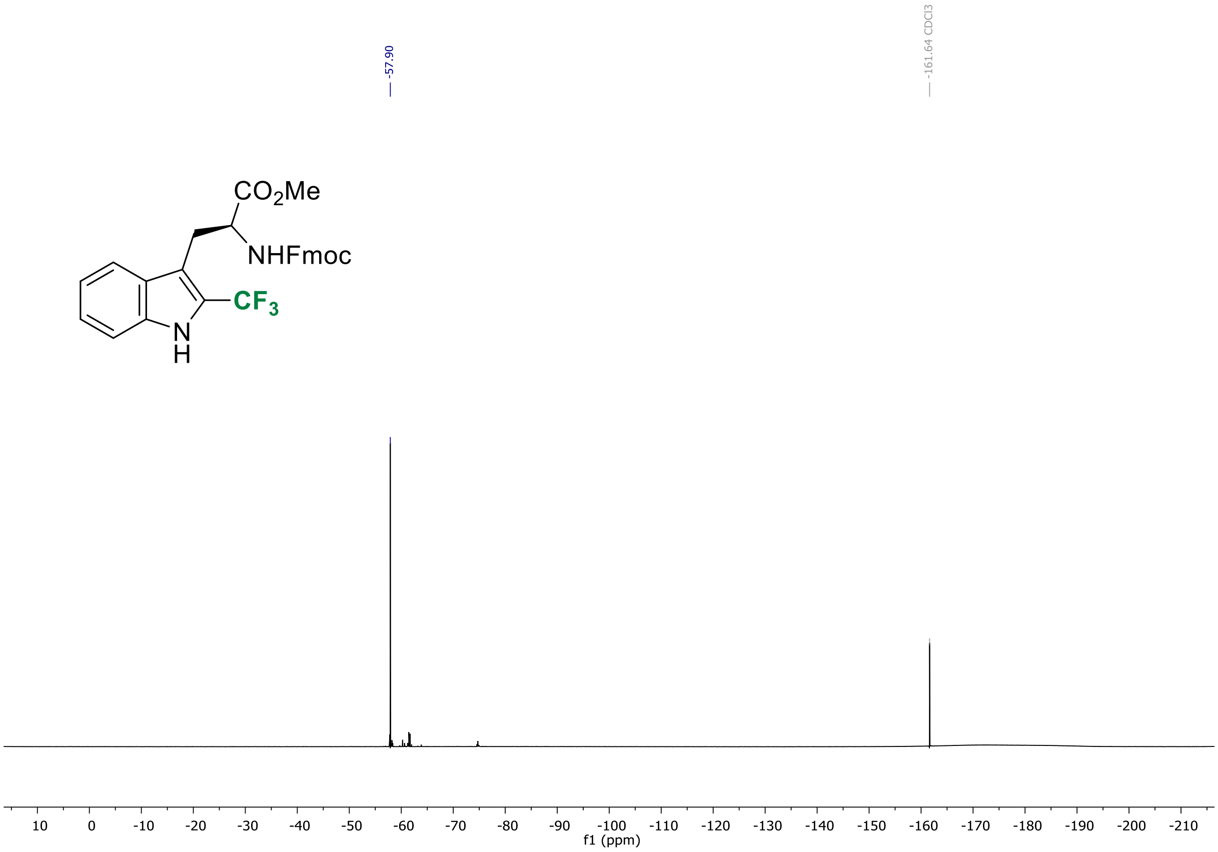

Figure S30: ^{1}H NMR spectrum of **7a** in $CDCl_3$, 400 MHz

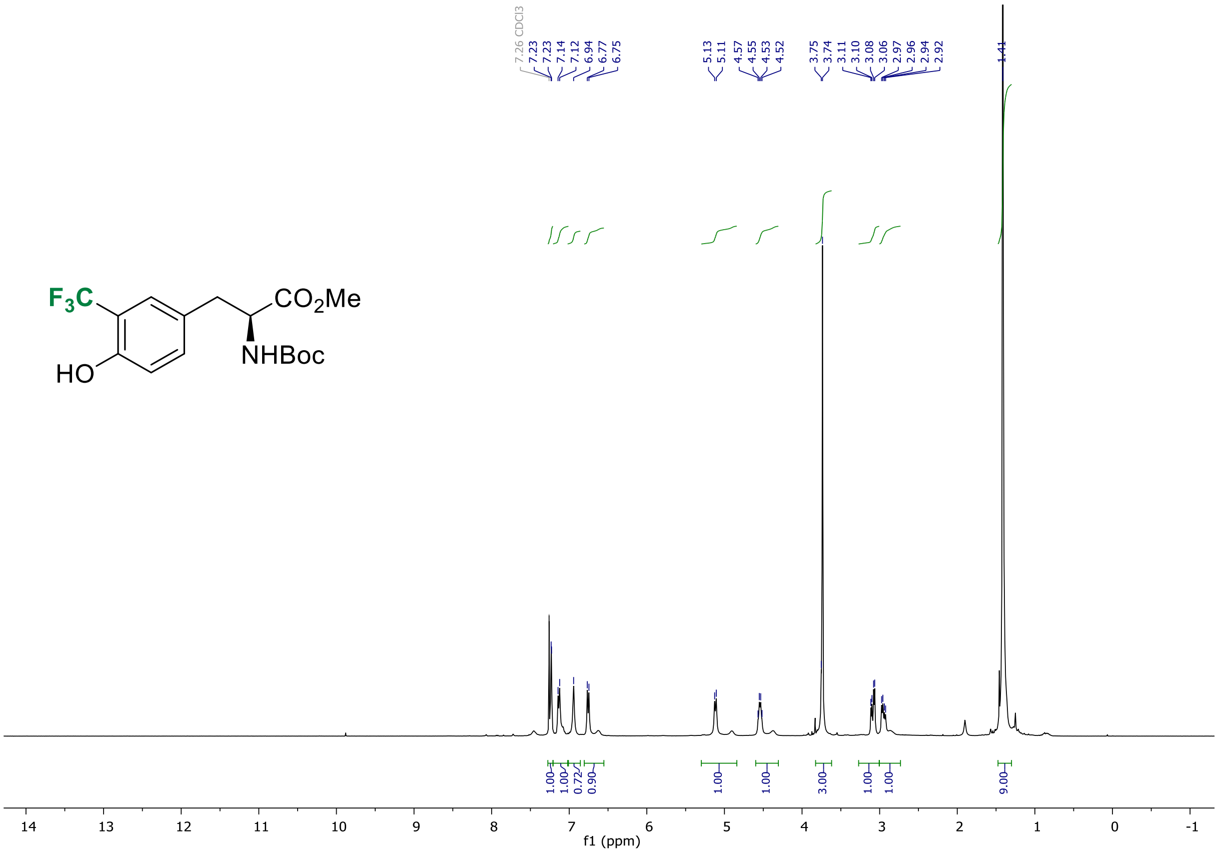

Figure S31: $^{13}C\{^{1}H\}$ NMR spectrum of **7a** in $CDCl_3$, 101 MHz

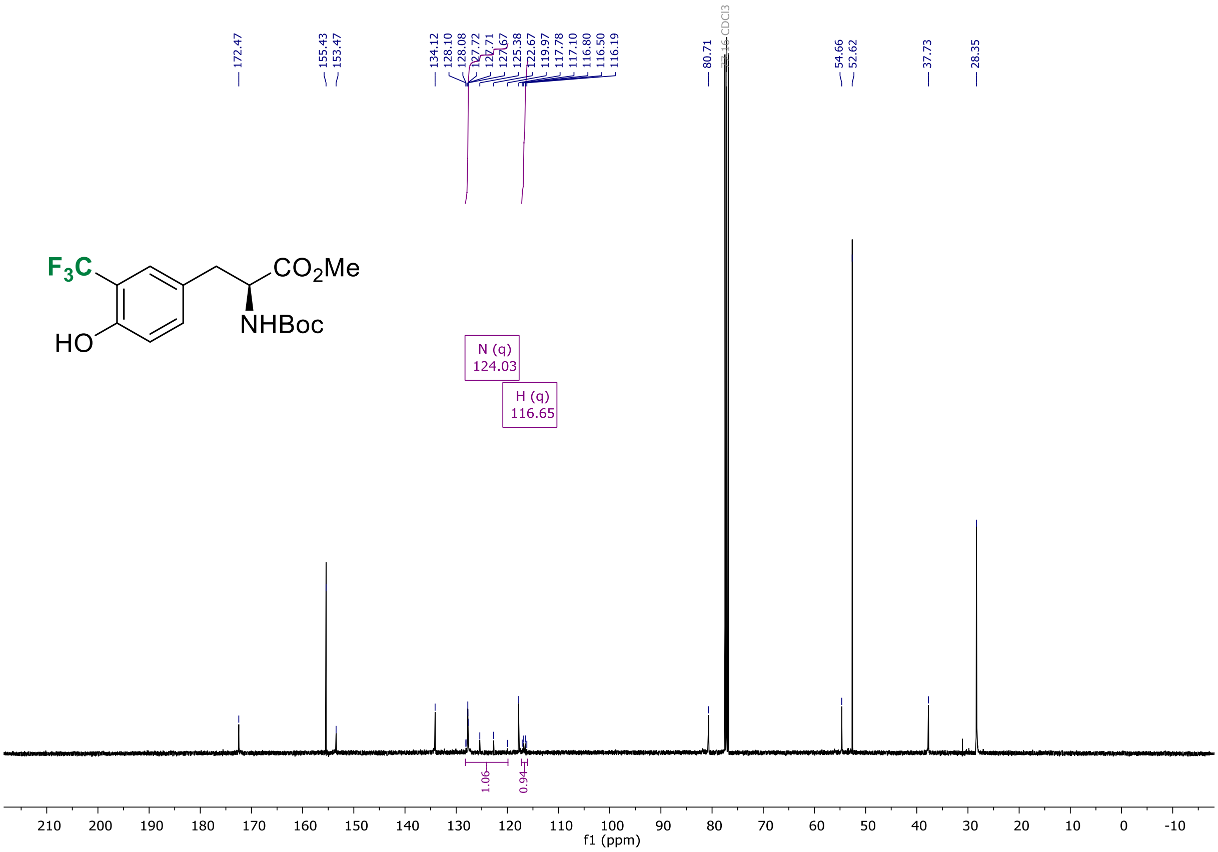

Figure S32: ^{19}F NMR spectrum of **7a** in $CDCl_3$, 376 MHz

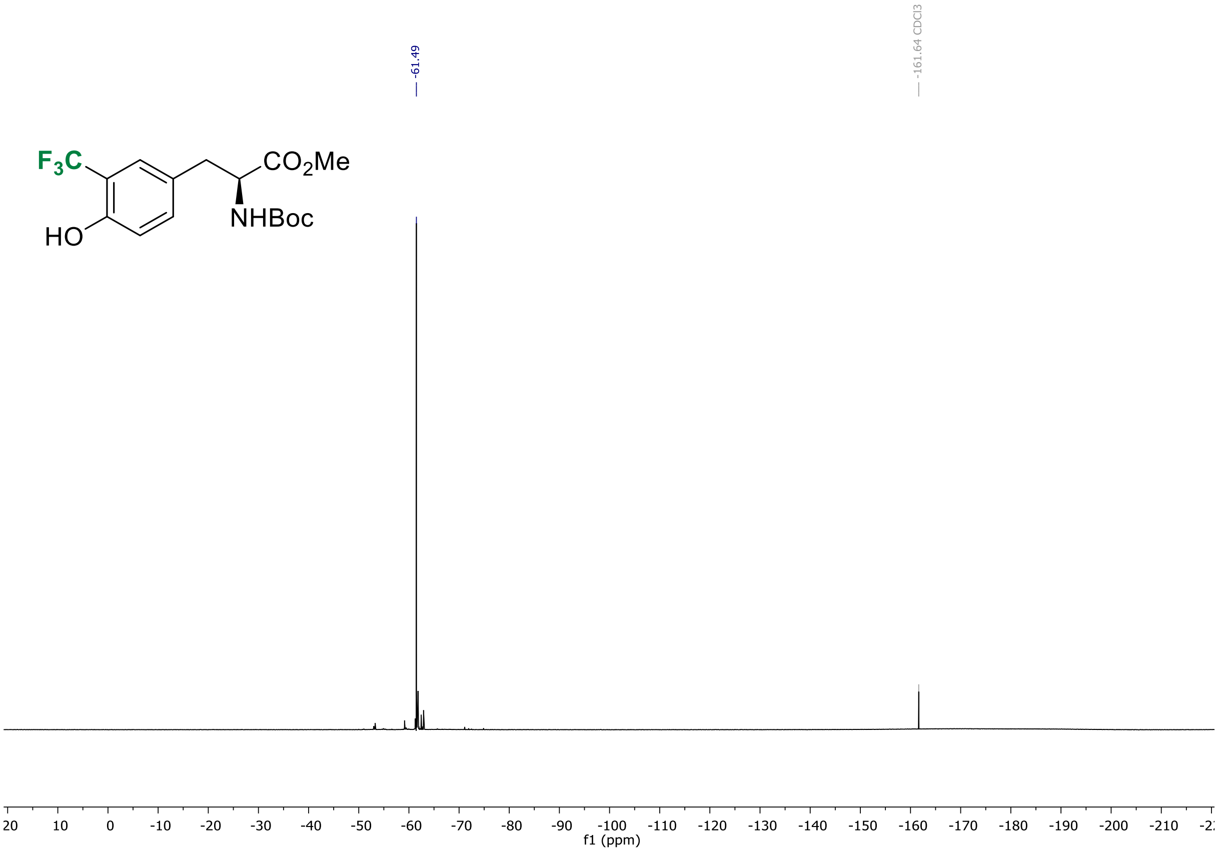

Figure S33: ^{1}H NMR spectrum of **7b** in $CDCl_3$, 400 MHz

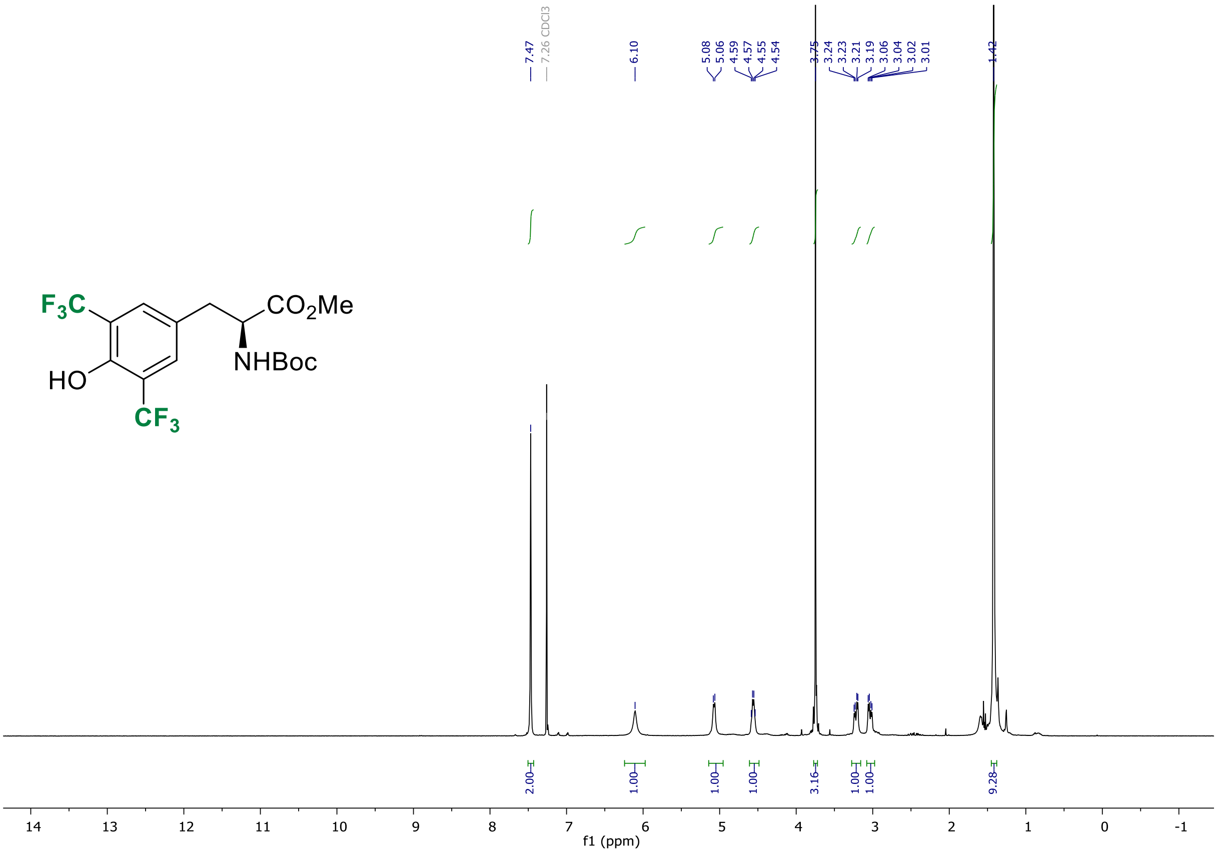

Figure S34: ^{13}C{^{1}H} NMR spectrum of **7b** in $CDCl_3$, 101 MHz

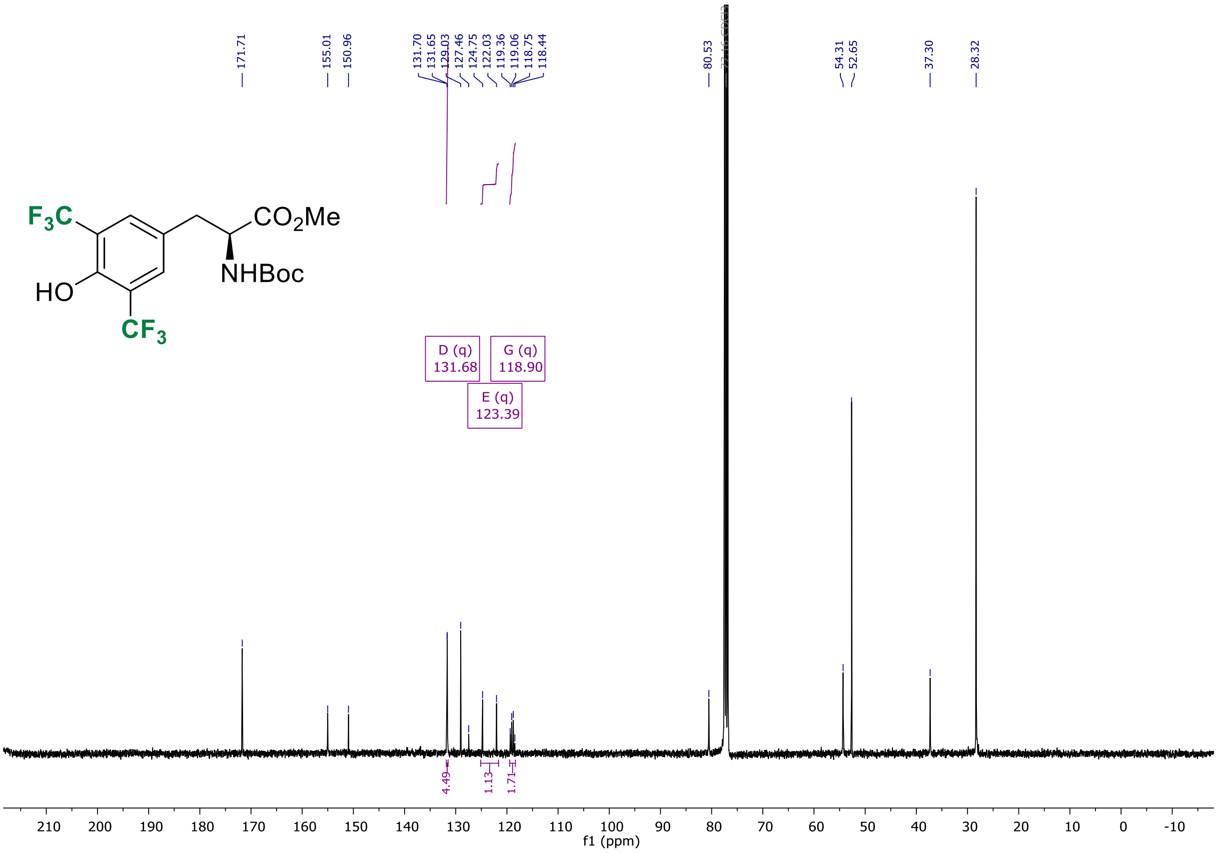

Figure S35: ^{19}F NMR spectrum of **7b** in $CDCl_3$, 376 MHz

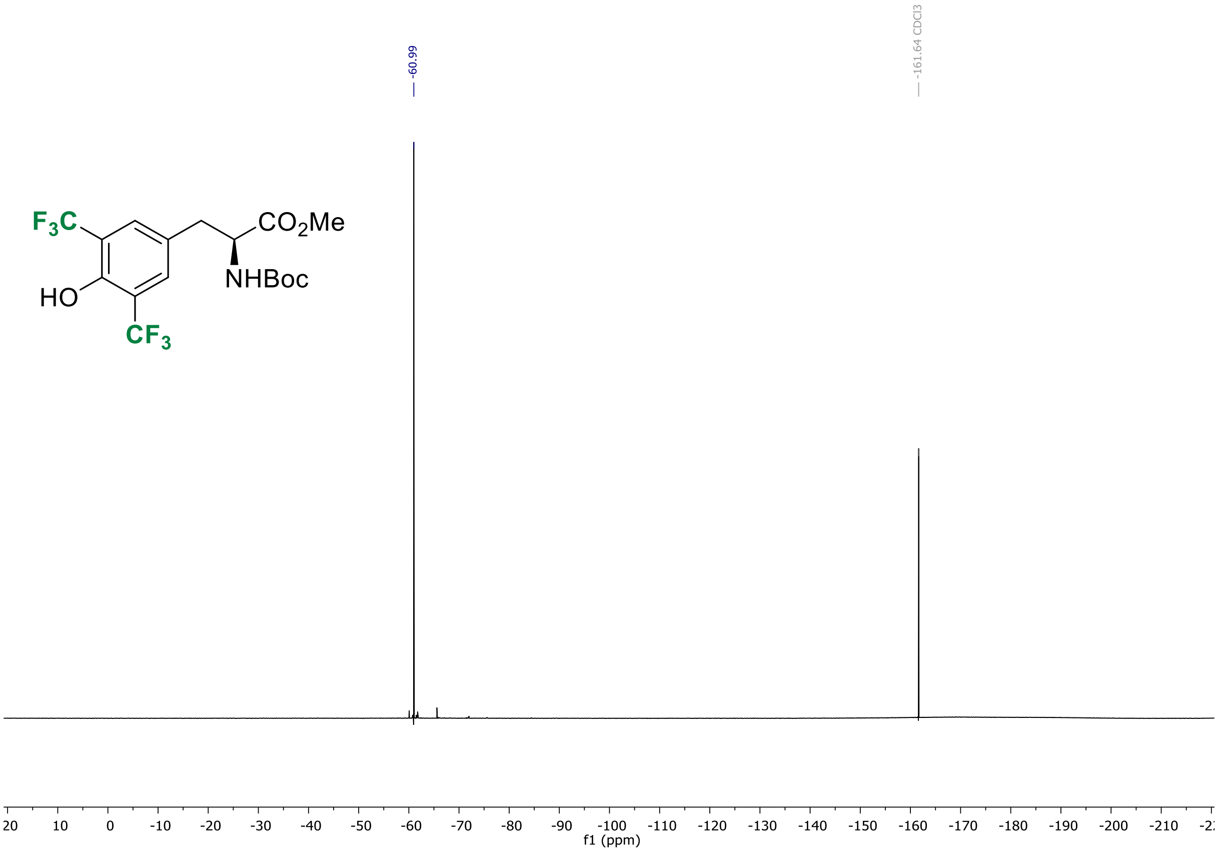

Figure S36: ^{1}H NMR spectrum of **8a** in MeOD-d_4, 400 MHz

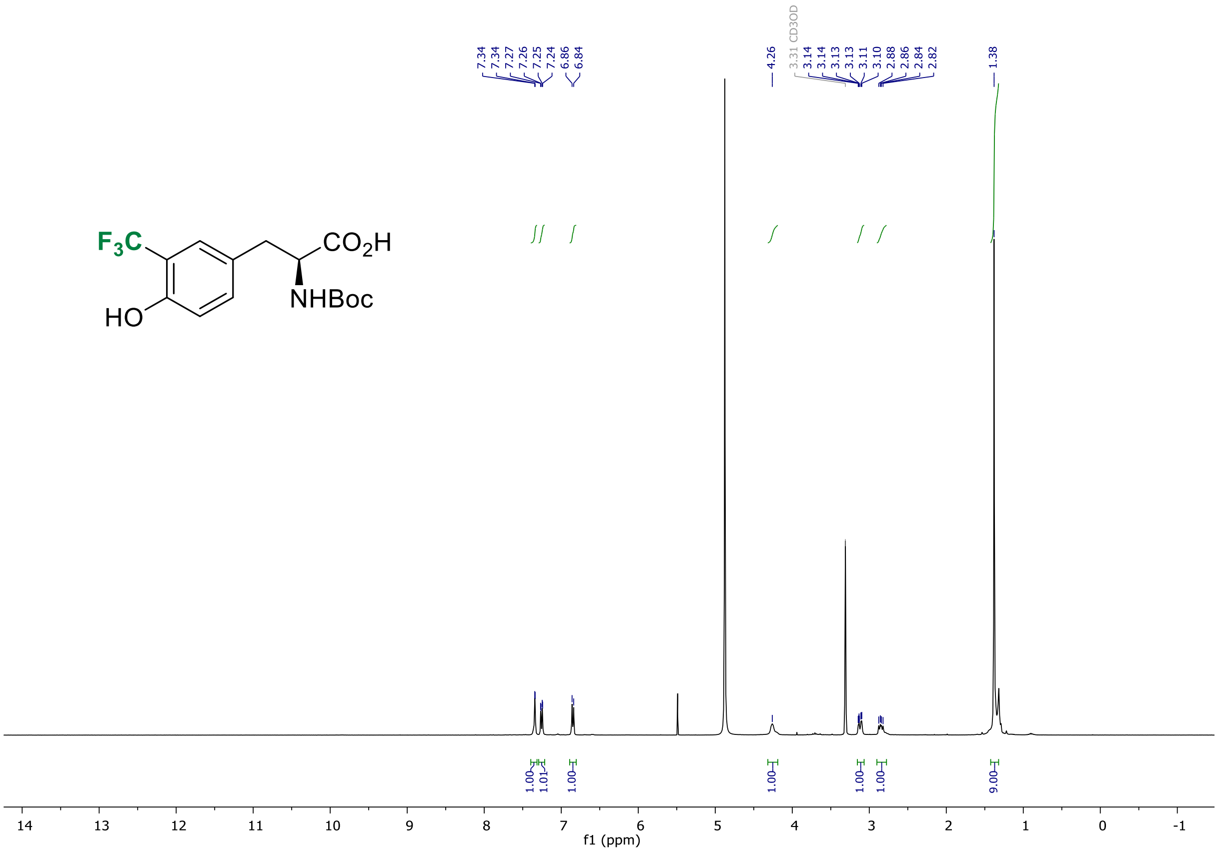

Figure S37: $^{13}C\{^{1}H\}$ NMR spectrum of **8a** in MeOD-d_4, 101 MHz

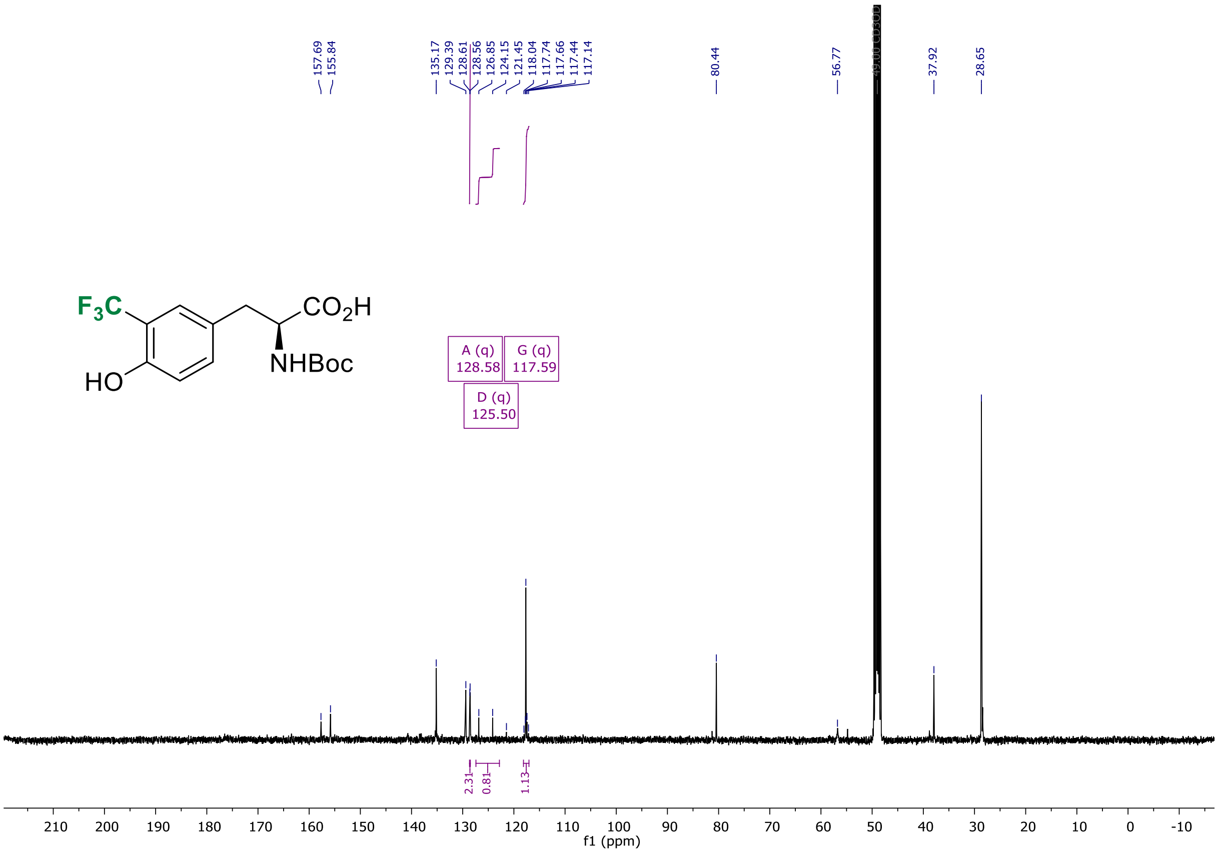

Figure S38: ^{19}F NMR spectrum of **8a** in MeOD-d_4, 376 MHz

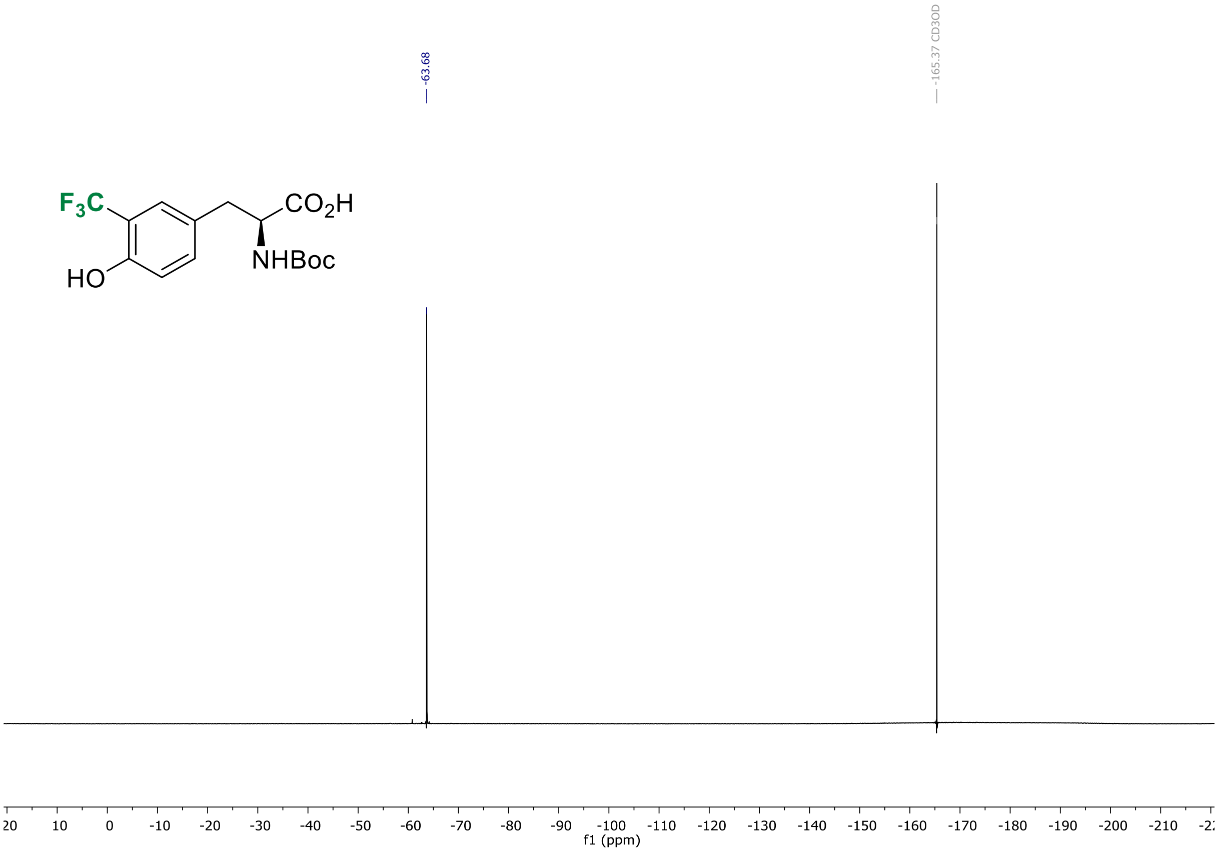

Figure S39: ^{1}H NMR spectrum of **8b** in MeOD-d_4, 400 MHz

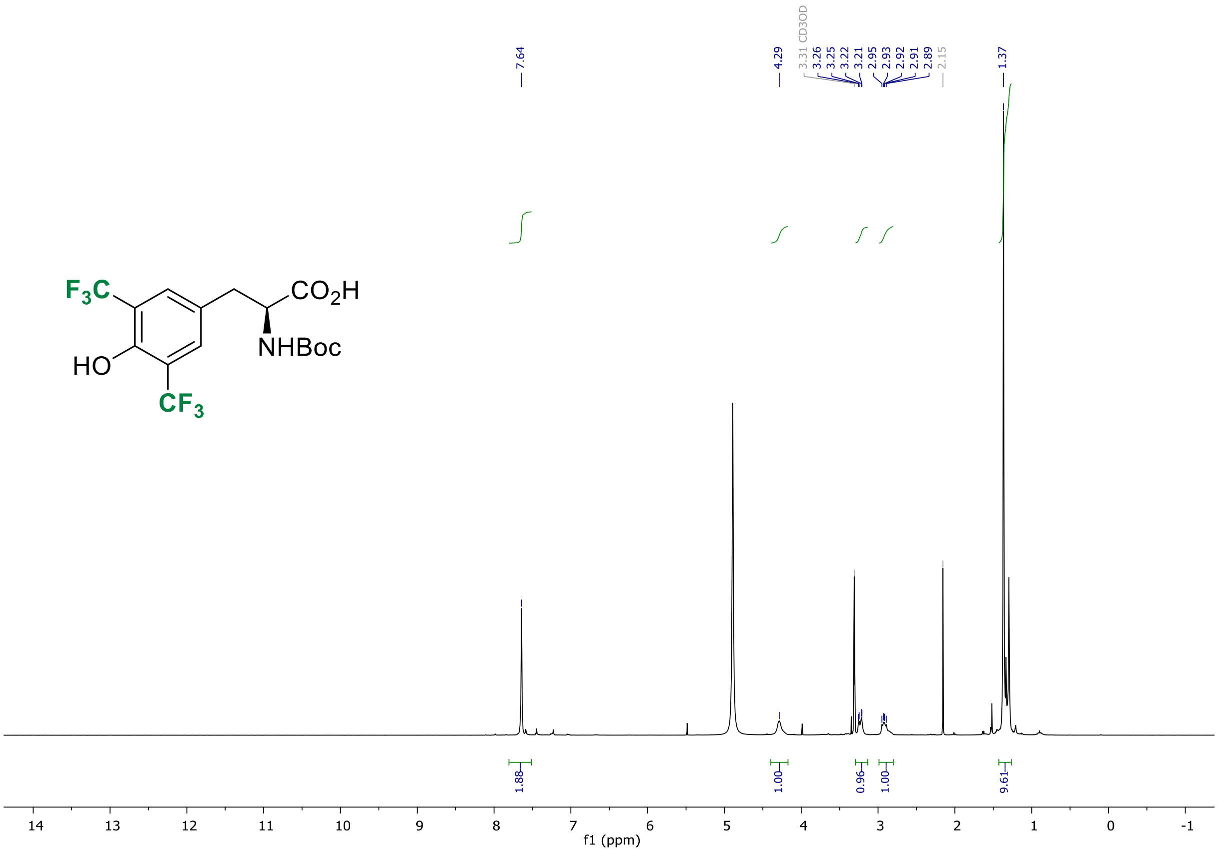

Figure S40: $^{13}C\{^{1}H\}$ NMR spectrum of **8b** in MeOD-d_4, 101 MHz

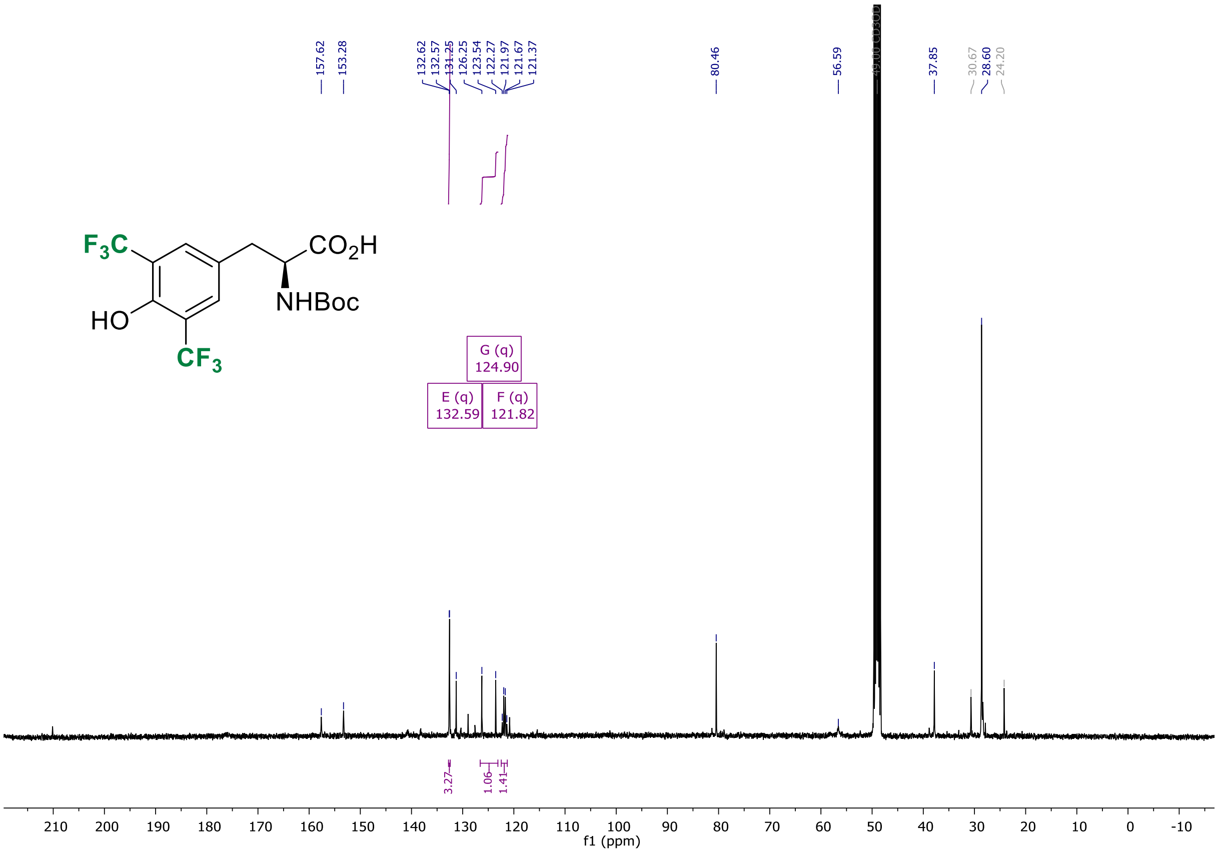

Figure S41: ^{19}F NMR spectrum of **8b** in MeOD-d_4, 376 MHz

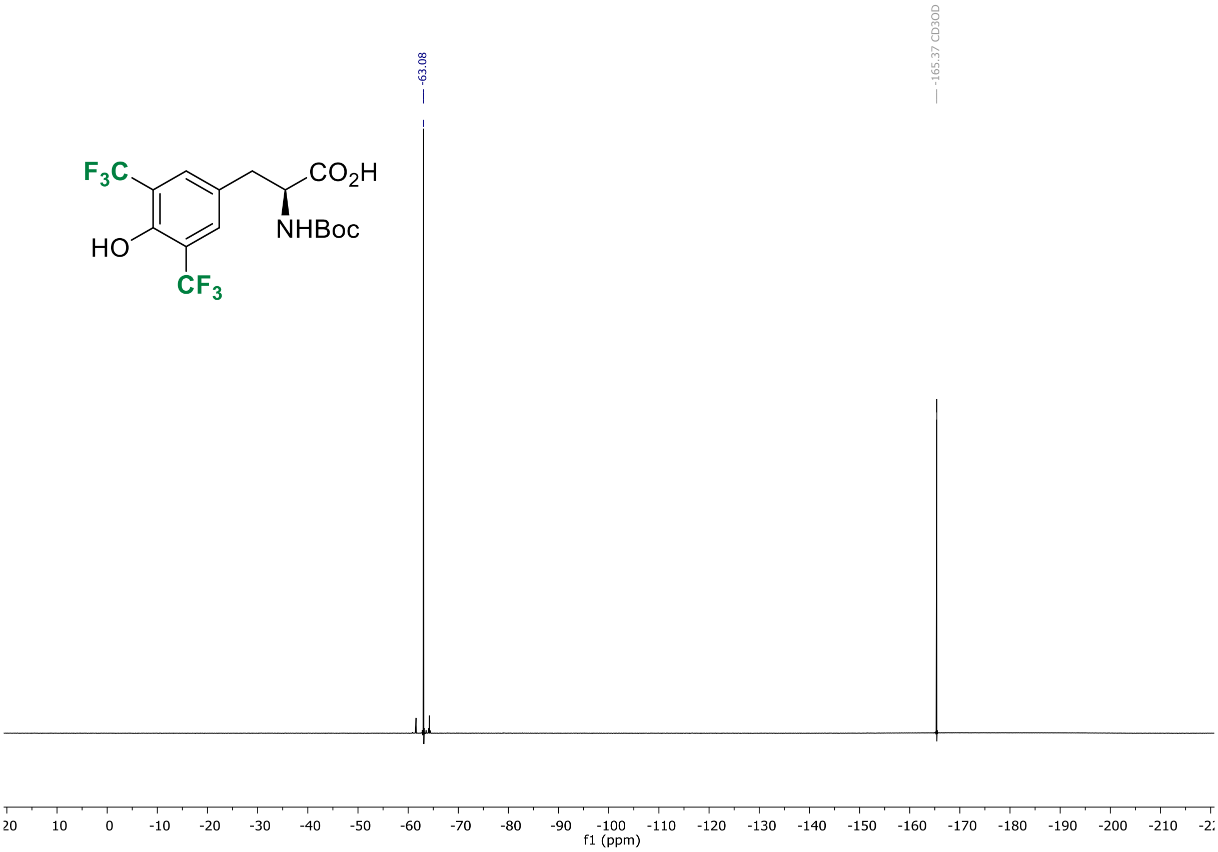

Figure S42: ^{1}H NMR spectrum of **11** in $CDCl_3$, 400 MHz

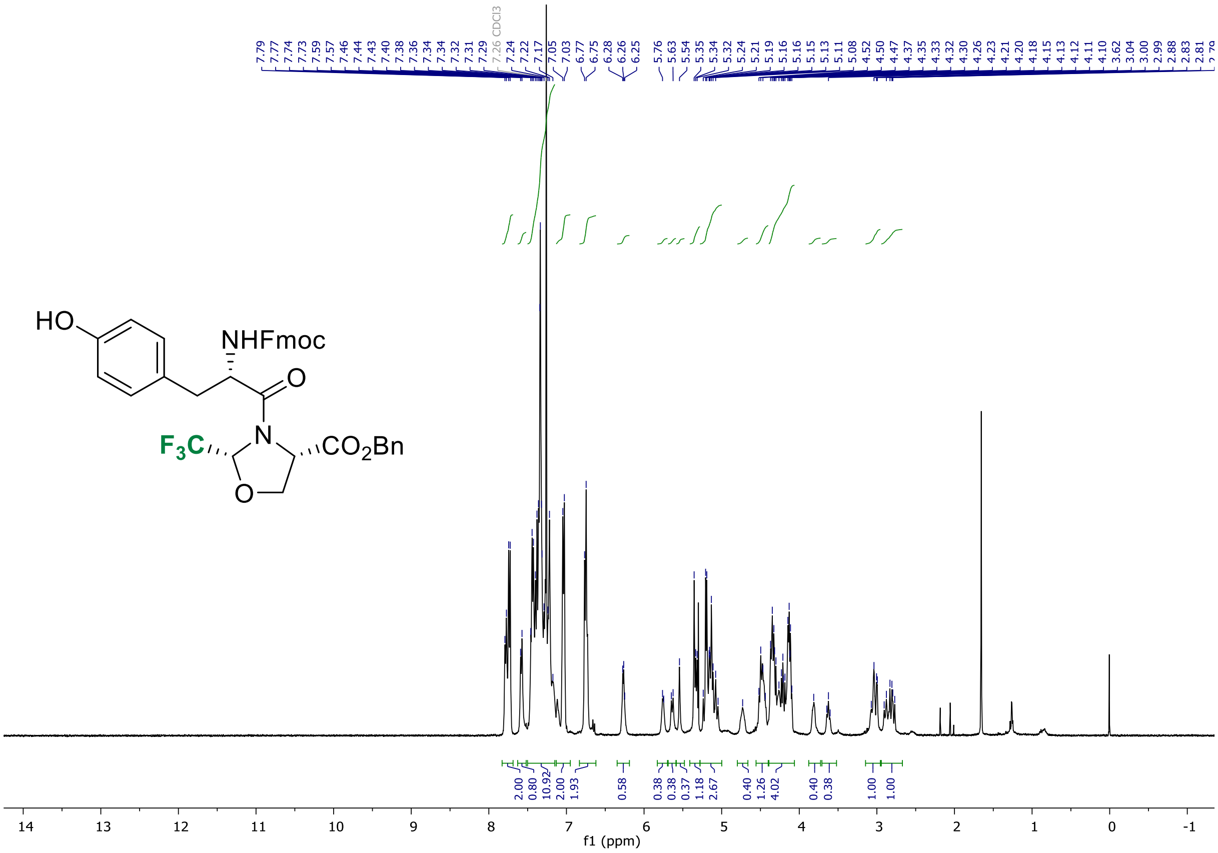

Figure S43: $^{13}C\{^1H\}$ NMR spectrum of **11** in $CDCl_3$, 101 MHz

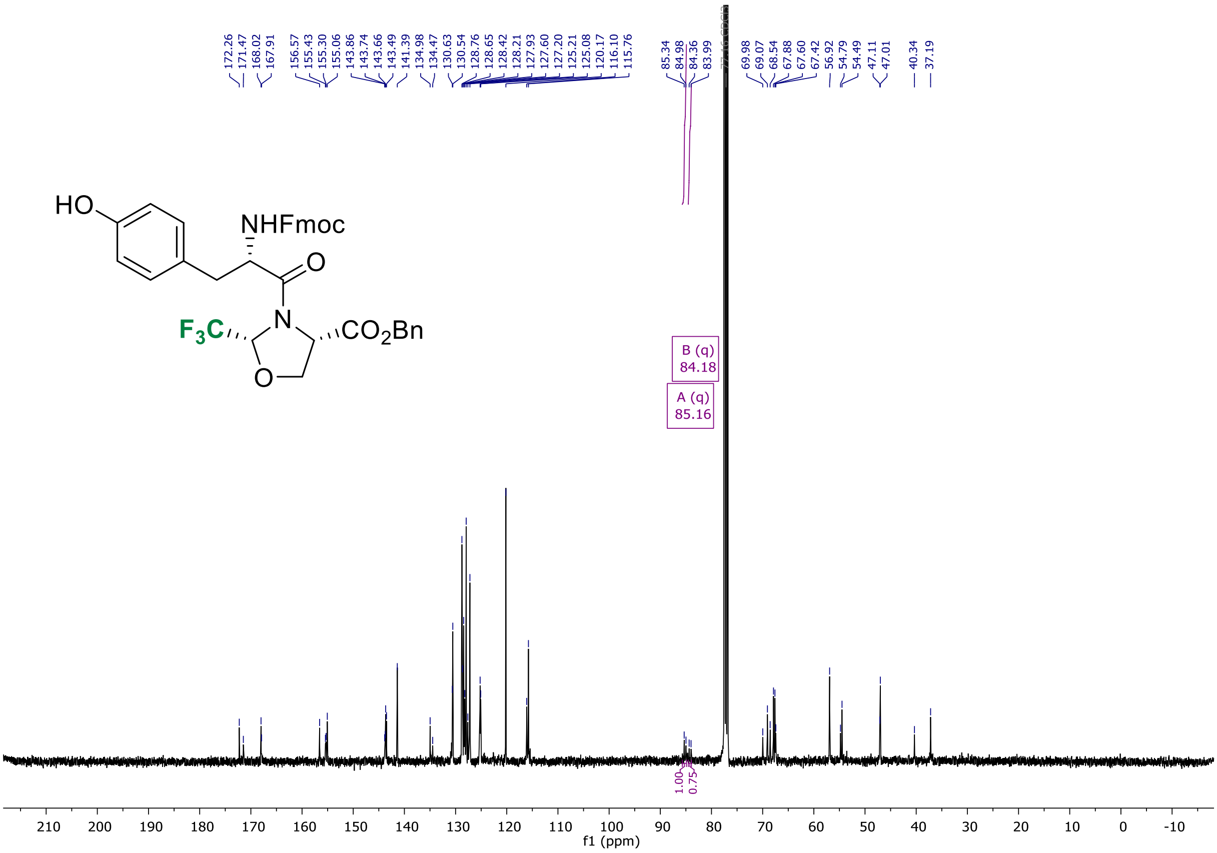

Figure S44: ^{19}F NMR spectrum of **11** in $CDCl_3$, 376 MHz

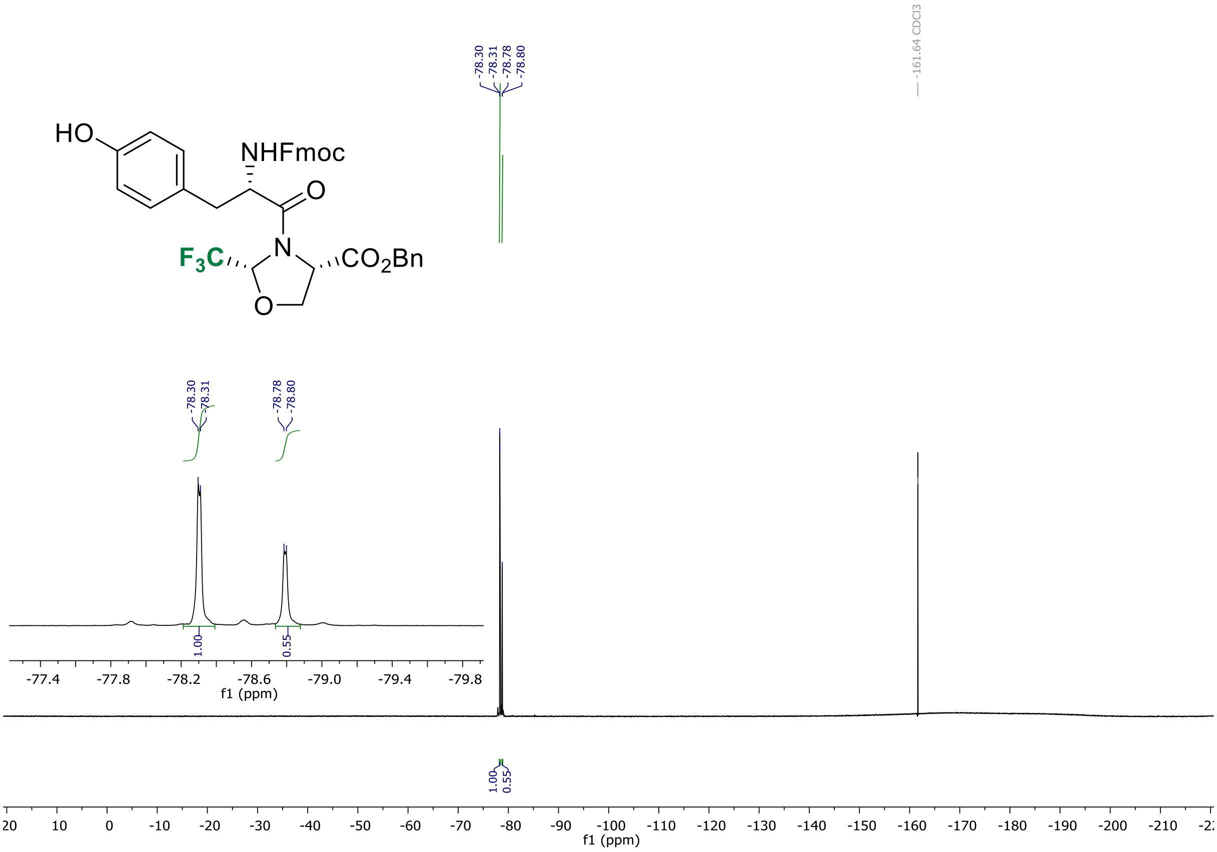

Figure S45: ^{1}H NMR spectrum of **12** in $CDCl_3$, 600 MHz

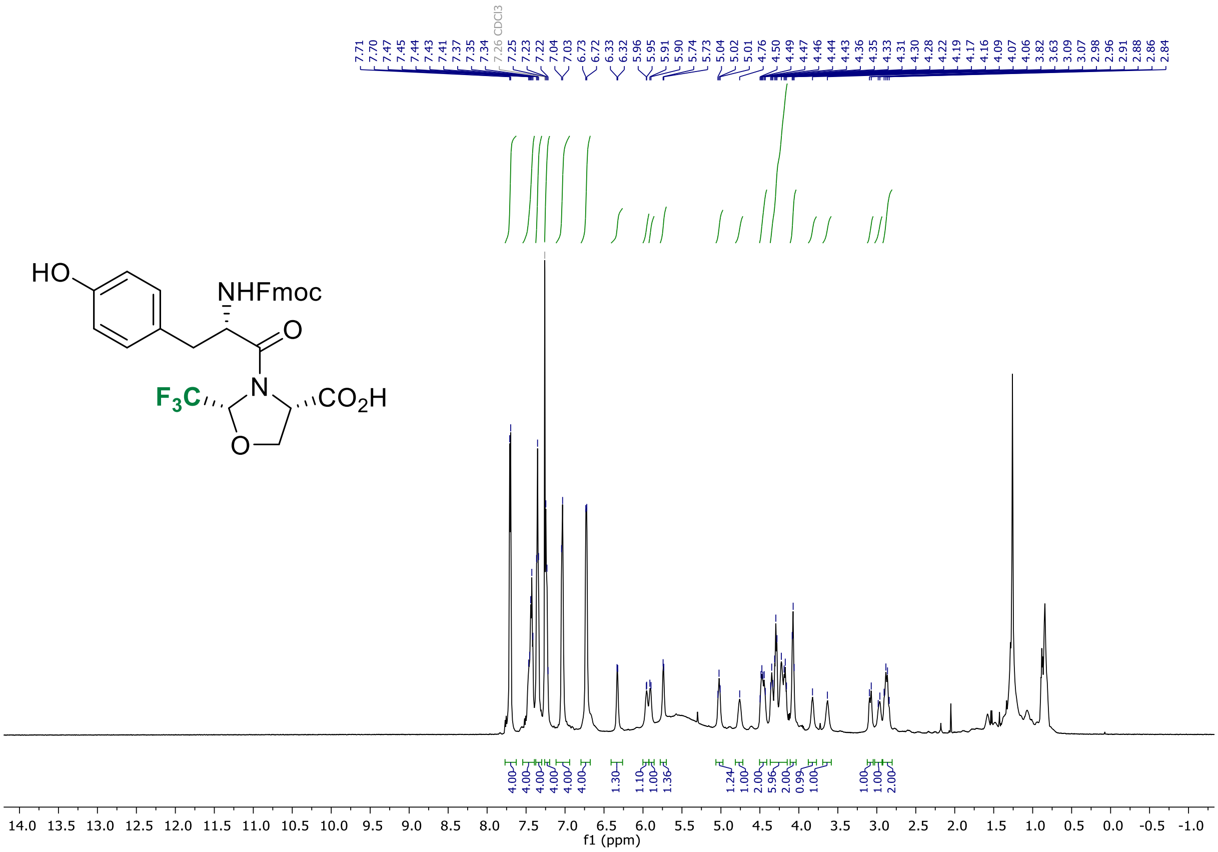

Figure S46: $^{13}C\{^{1}H\}$ NMR spectrum of **12** in $CDCl_3$, 151 MHz

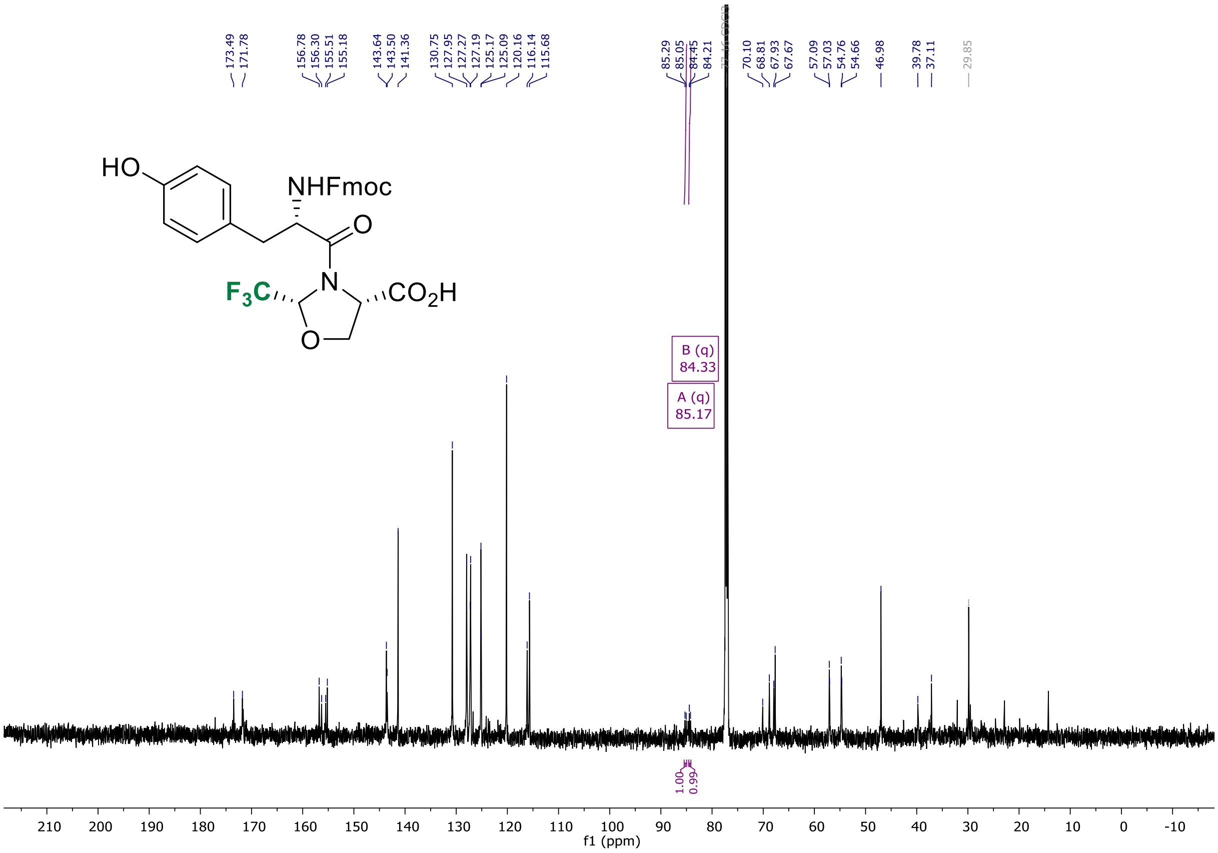

Figure S47: ^{19}F NMR spectrum of **12** in $CDCl_3$, 565 MHz

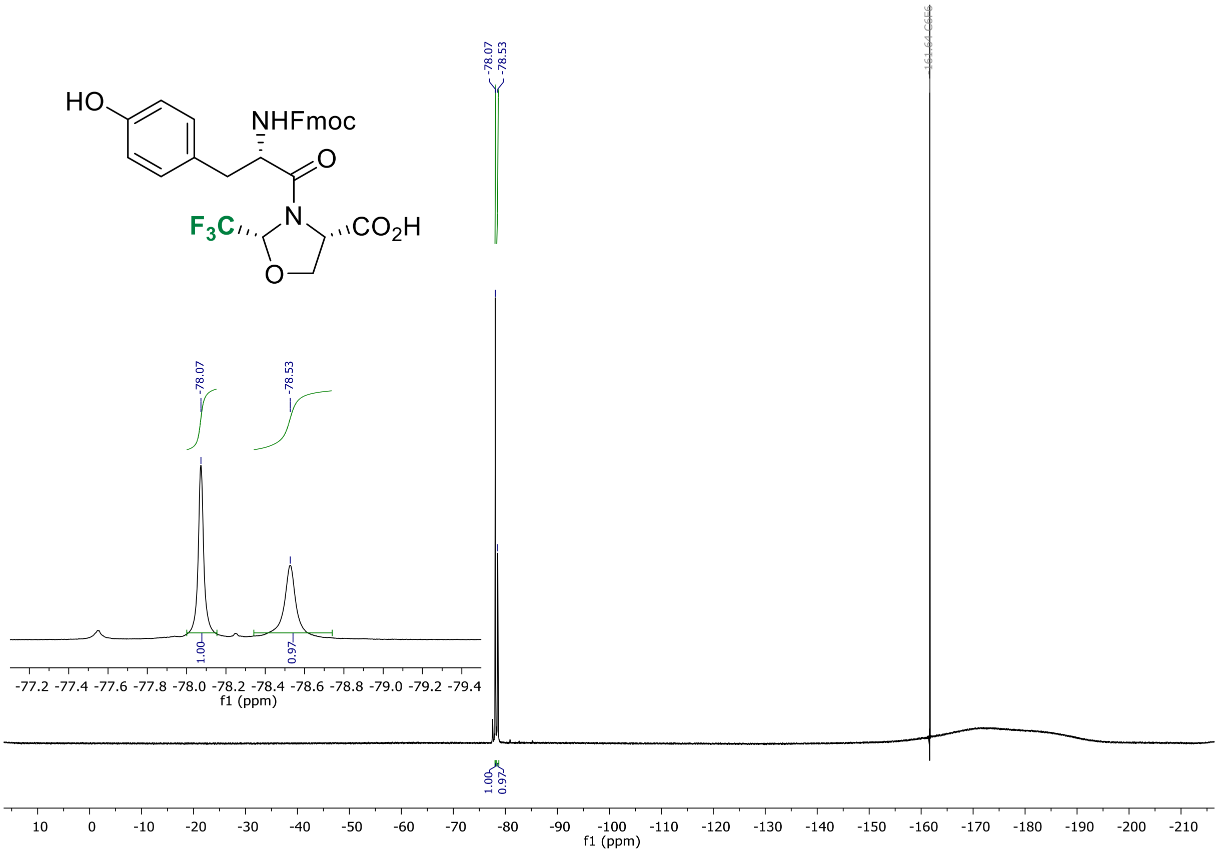

Opioid ligands

Figure S48: ^{1}H NMR spectrum of **EM1** in MeOD-d_3, 400 MHz (20 mM, water suppression)

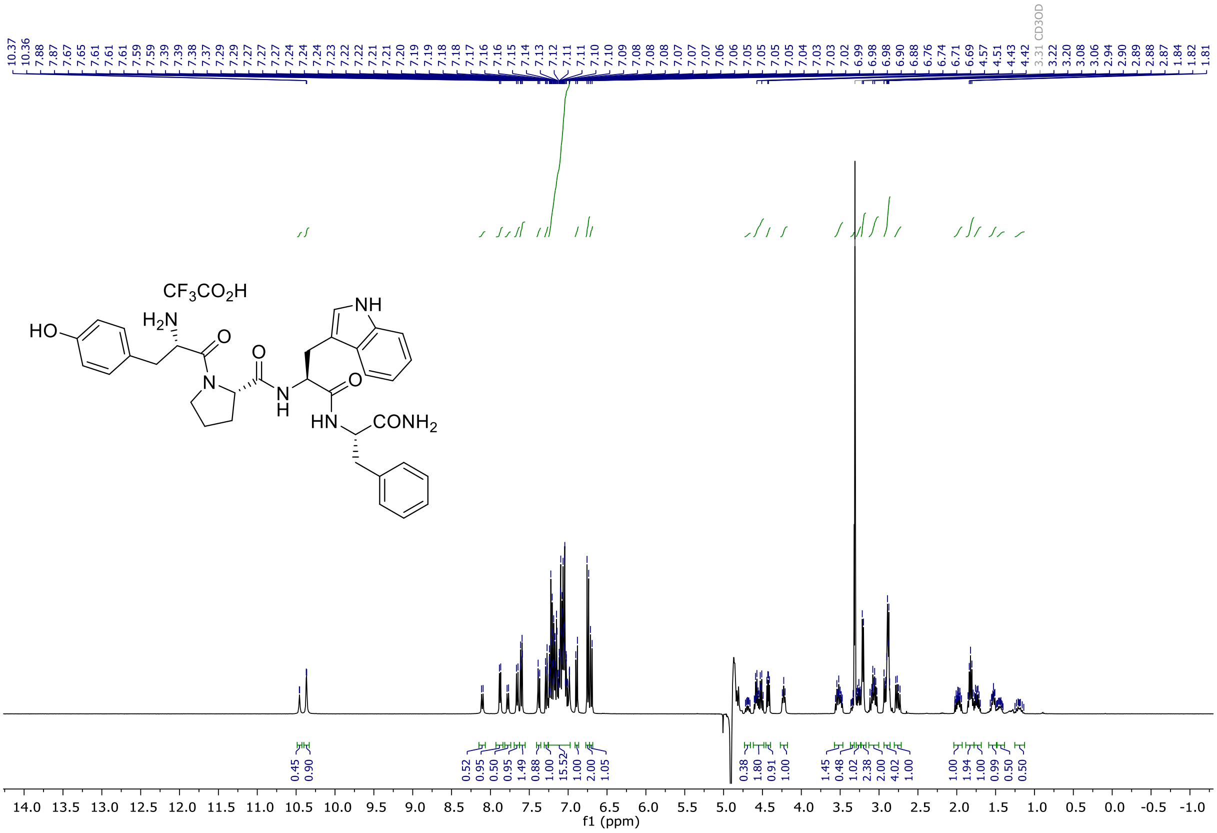

Figure S49: $^{13}C\{^{1}H\}$ NMR spectrum of **EM1** in MeOD-d_3, 101 MHz (20 mM)

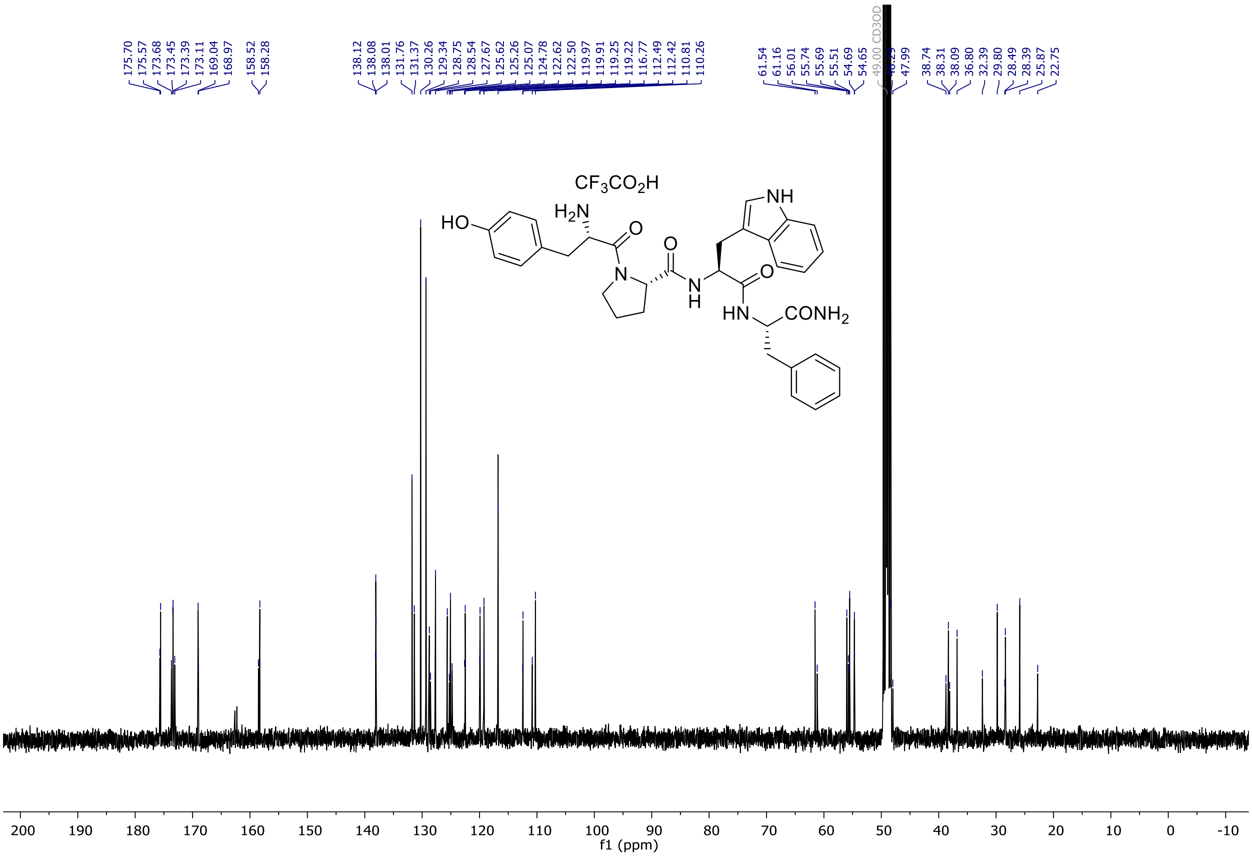

Figure S50: ^{1}H NMR spectrum of **L0** in MeOD-d_4, 400 MHz

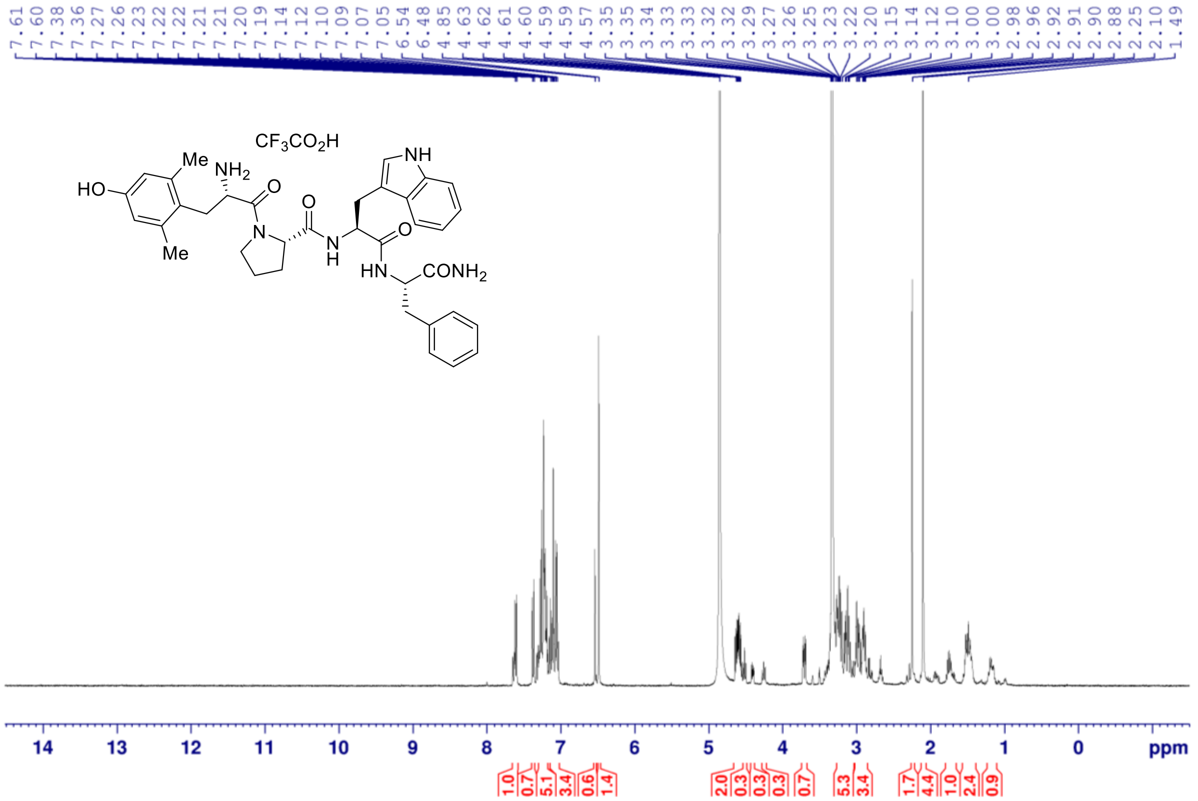

Figure S51: ^{1}H NMR spectrum of **L1** in MeOD-d_3, 400 MHz (20 mM, water suppression)

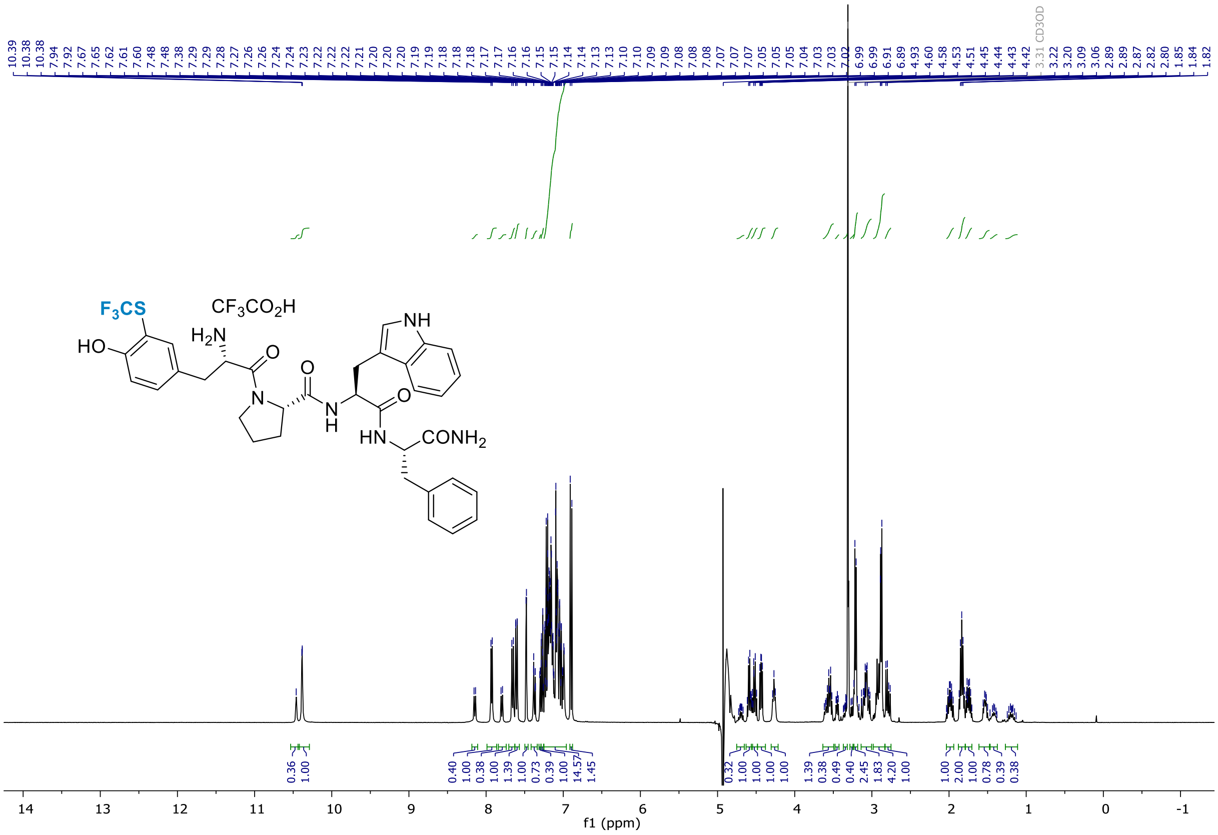

Figure S52: $^{13}C\{^{1}H\}$ NMR spectrum of **L1** in MeOD-d_3, 101 MHz (20 mM)

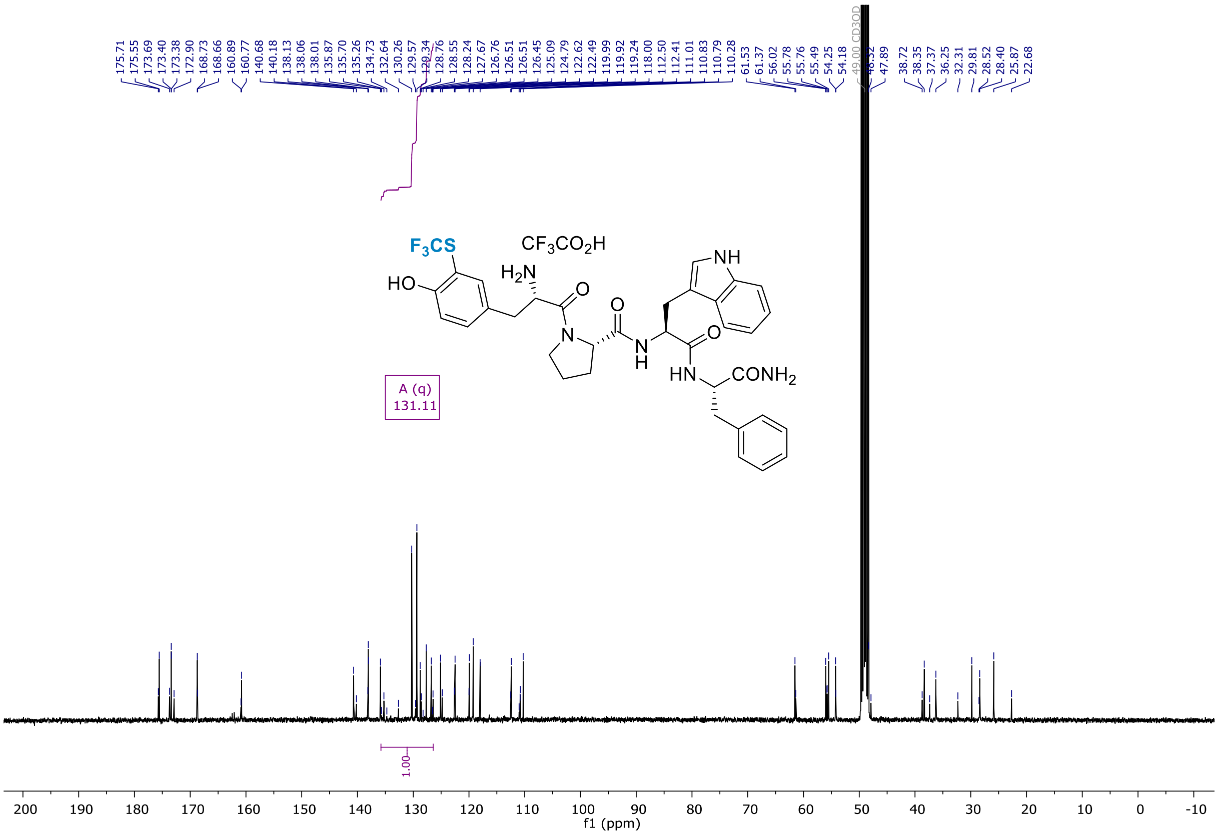

Figure S53: ^{19}F NMR spectrum of **L1** in MeOD-d_3, 376 MHz (20 mM)

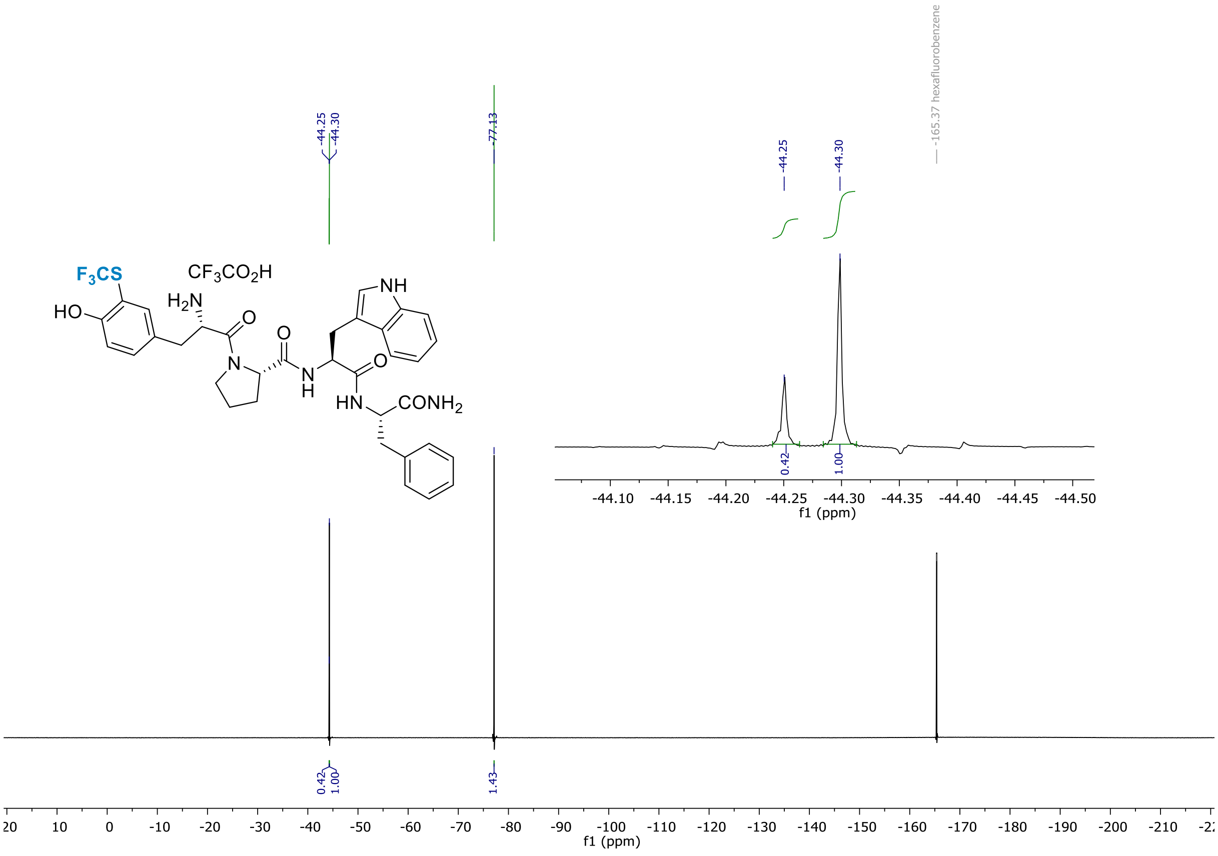

Figure S54: ^{1}H NMR spectrum of **L2** in MeOD-d_4, 600 MHz (20 mM)

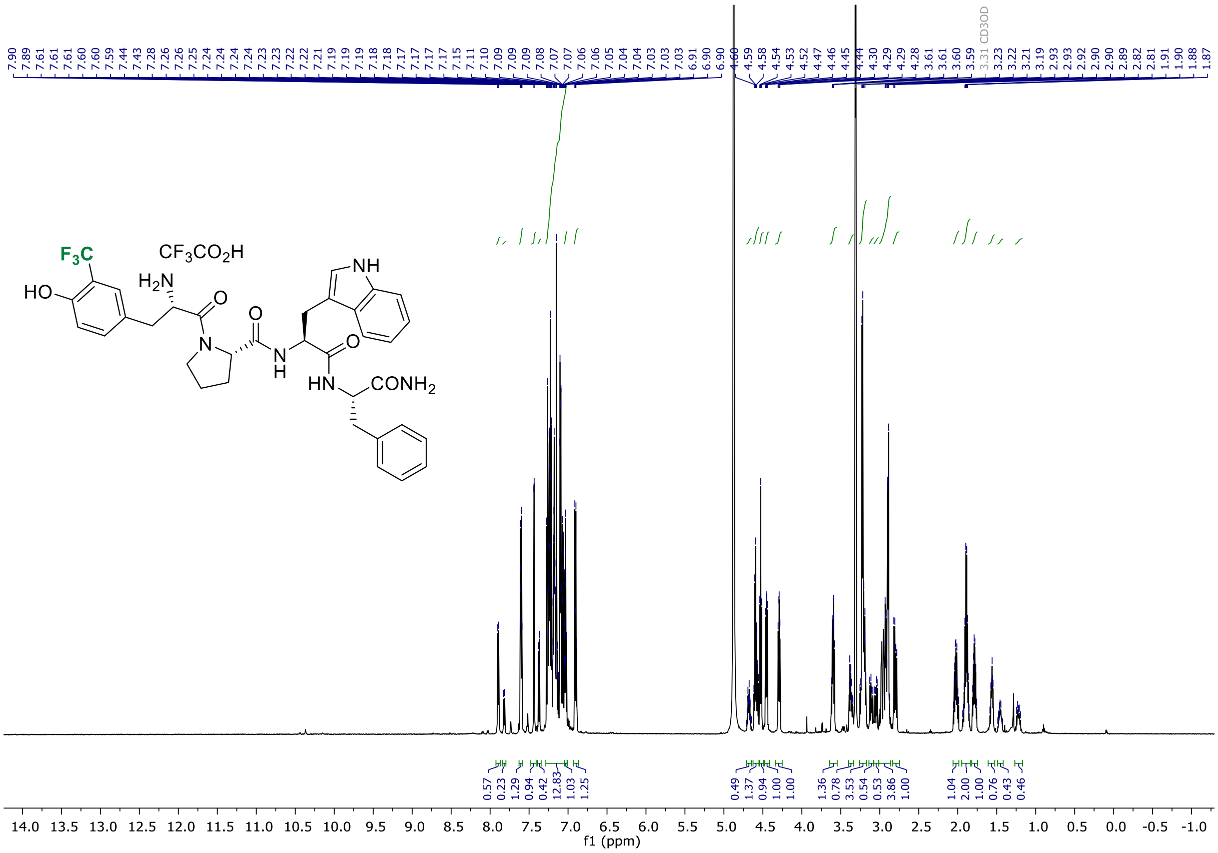

Figure S55: $^{13}C\{^{1}H\}$ NMR spectrum of **L2** in MeOD-d_4, 151 MHz (20 mM)

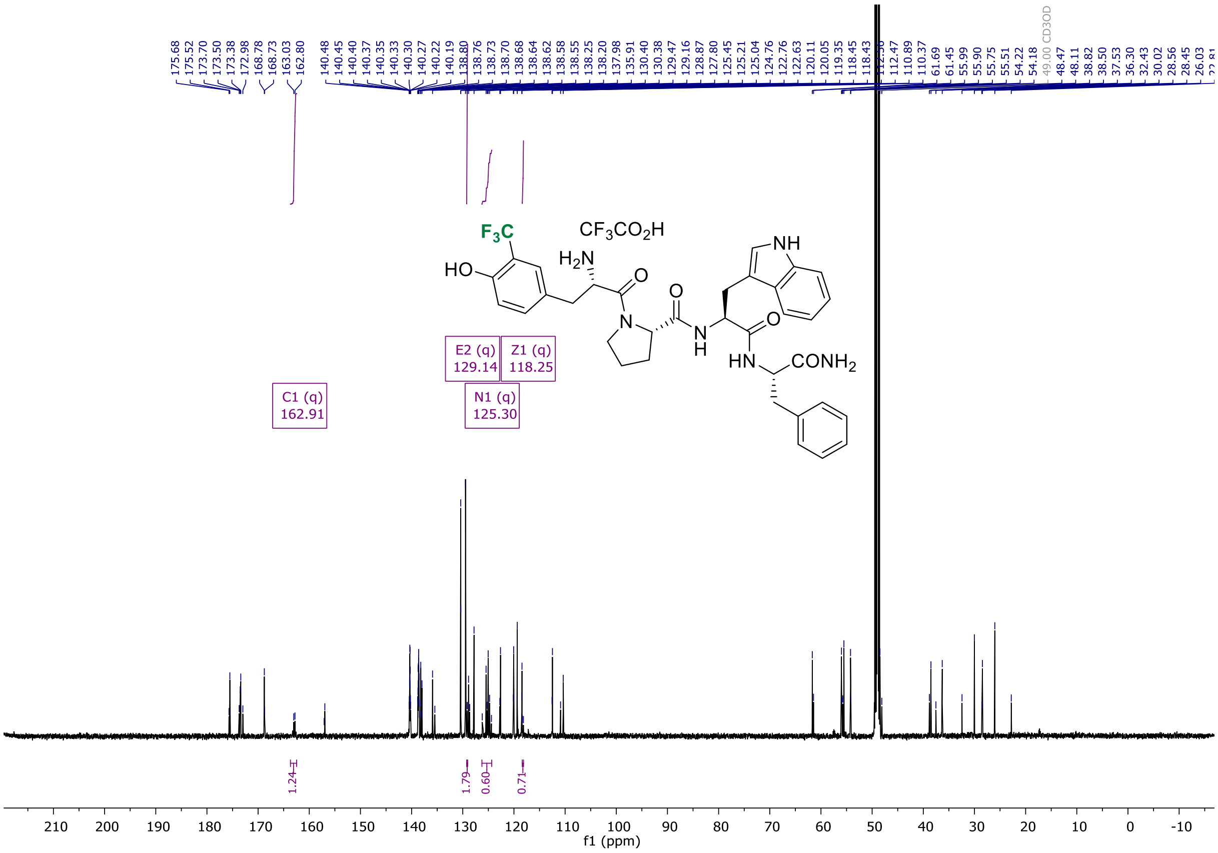

Figure S56: ^{19}F NMR spectrum of **L2** in MeOD-d_4, 565 MHz (20 mM)

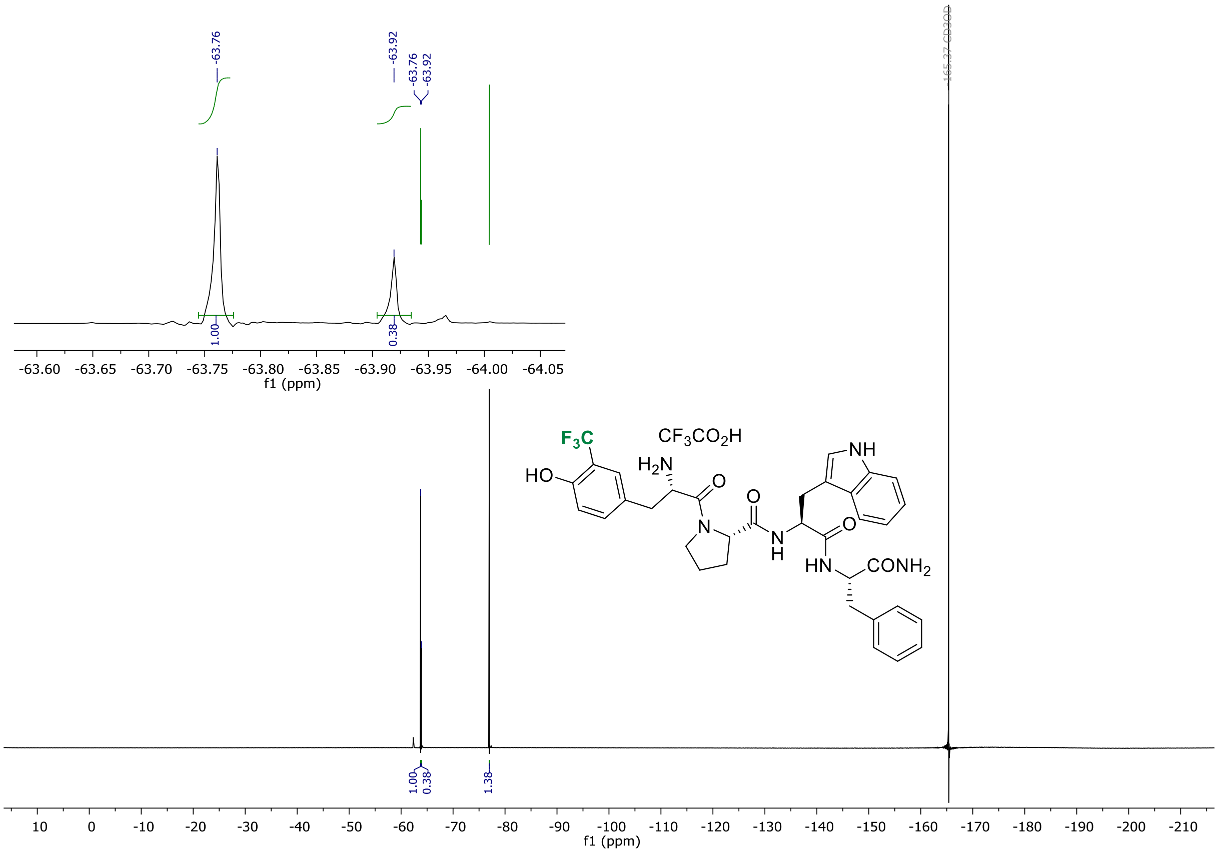

Figure S57: ^{1}H NMR spectrum of **L3** in MeOD-d_4, 600 MHz (18 mM)

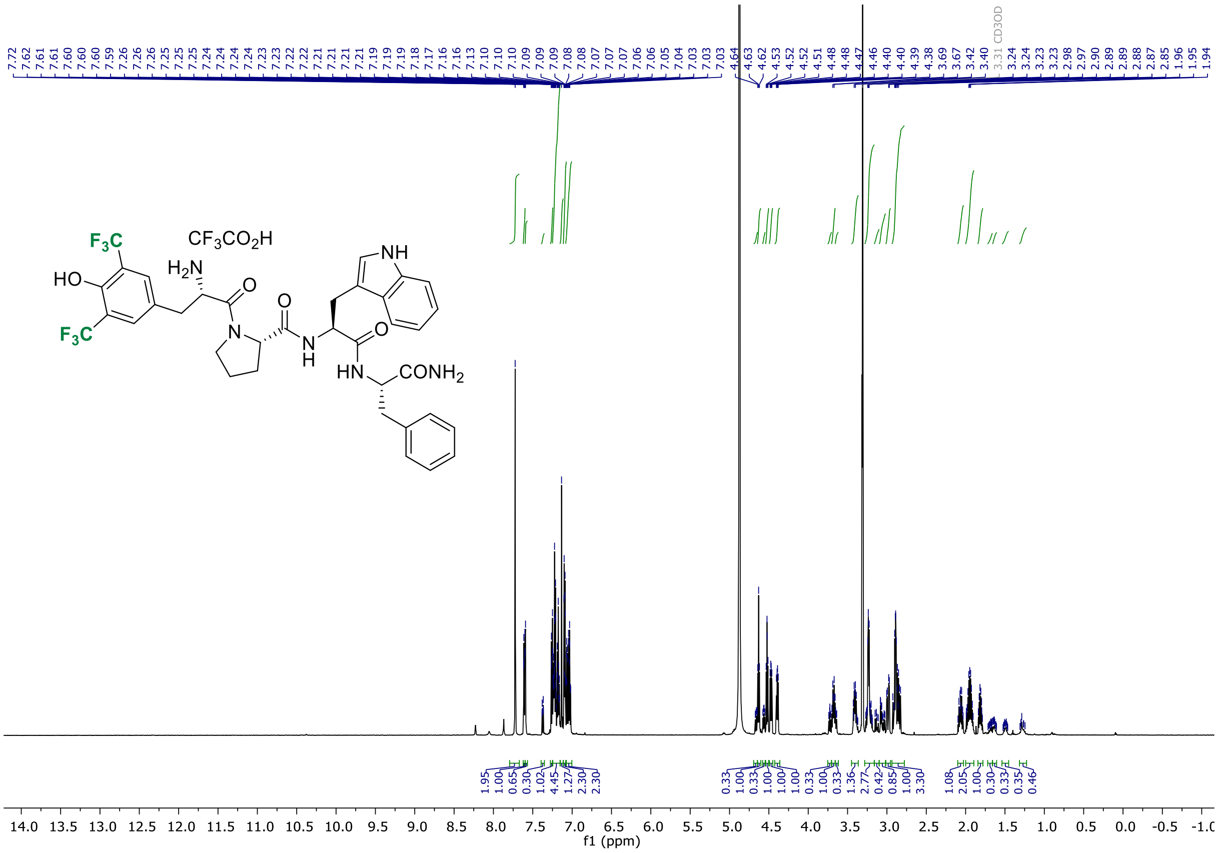

Figure S58: $^{13}C\{^{1}H\}$ NMR spectrum of **L3** in MeOD-d_4, 151 MHz (18 mM)

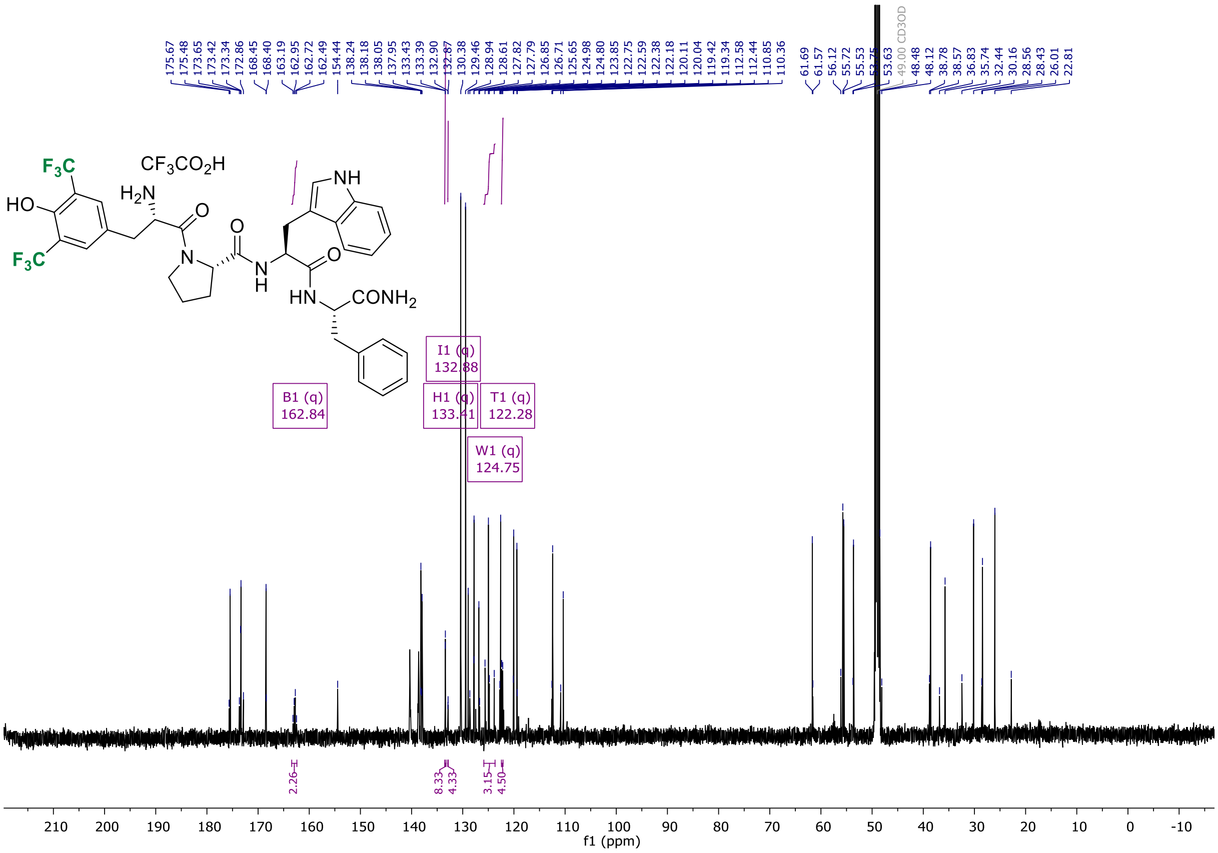

Figure S59: ^{19}F NMR spectrum of **L3** in MeOD-d_4, 565 MHz (18 mM)

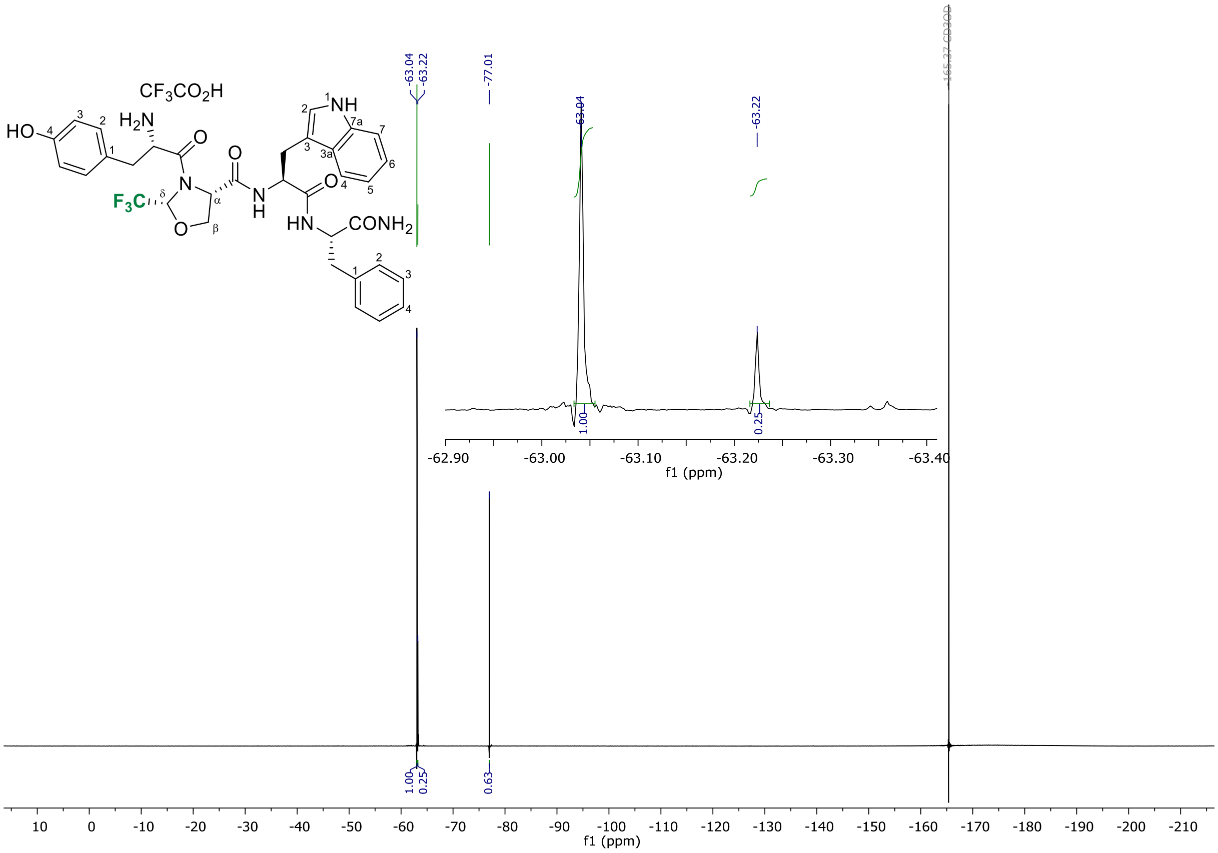

Figure S60: ^{1}H NMR spectrum of **L4** in MeOD-*d*$_4$, 400 MHz

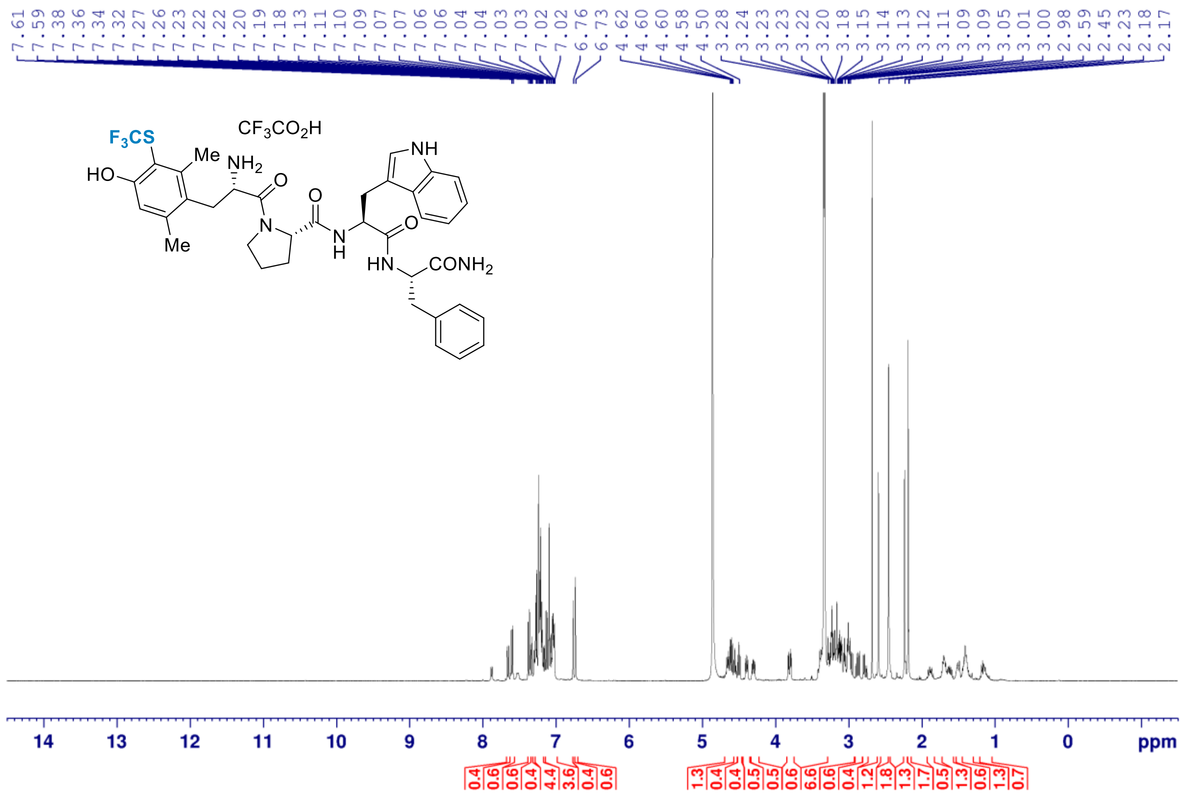

Figure S61: ^{19}F NMR spectrum of **L4** in MeOD-*d*$_4$, 376 MHz

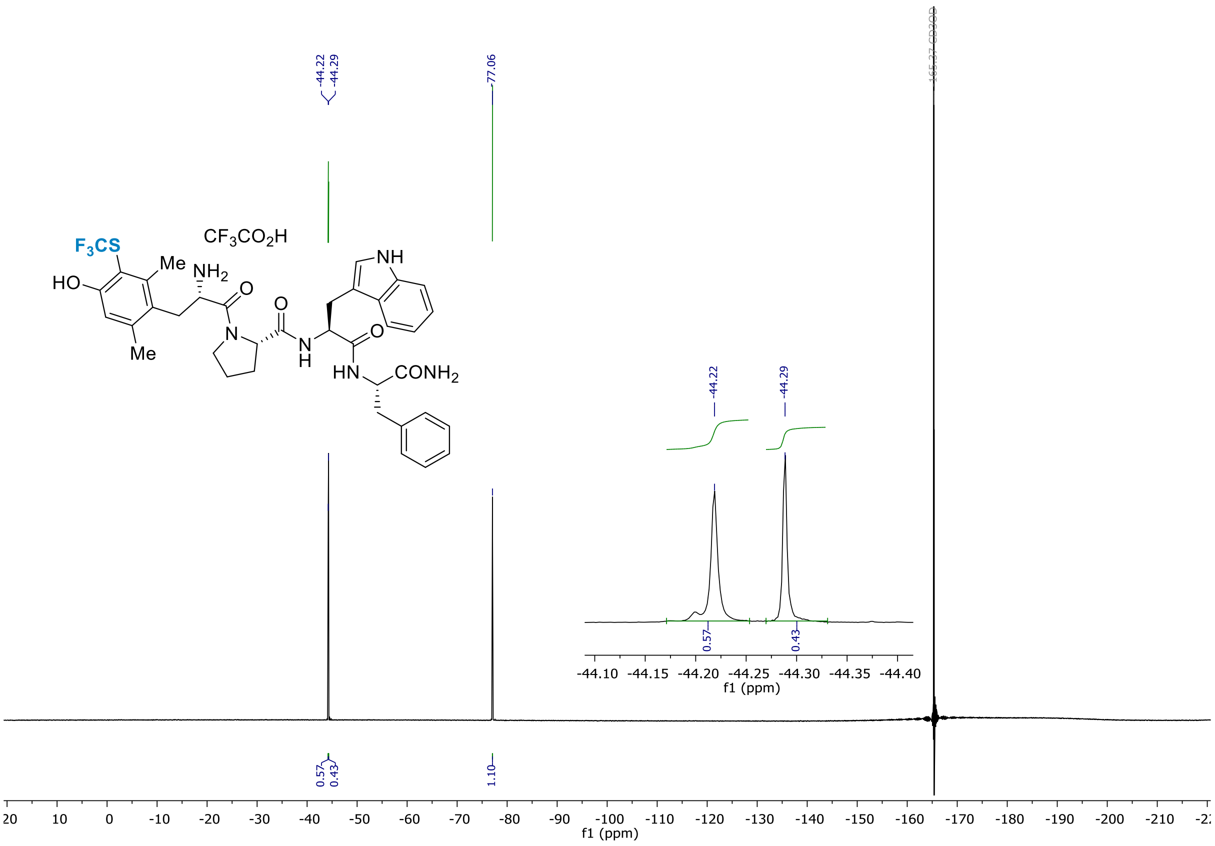

Figure S62: ^{1}H NMR spectrum of **L5** in MeOD-d_4, 400 MHz (18 mM, 10 °C)

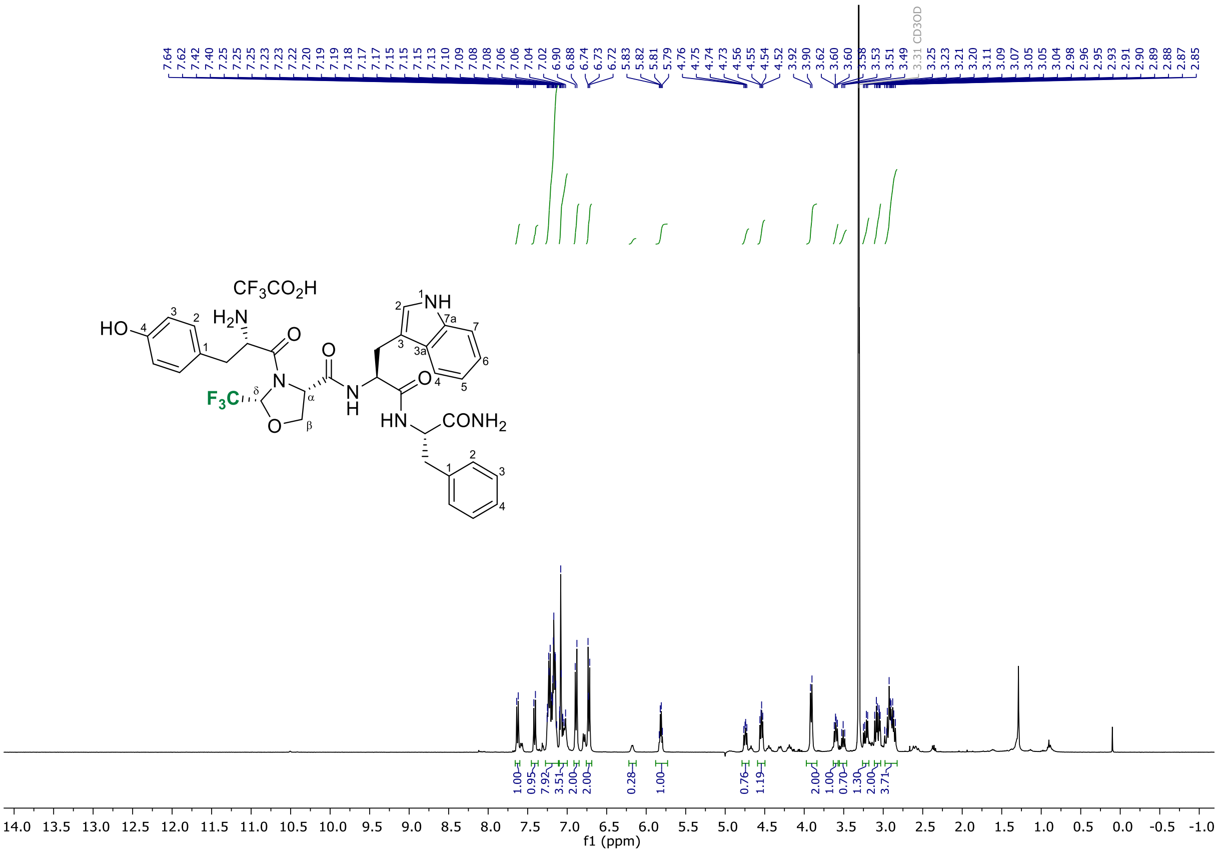

Figure S63: $^{13}C\{^{1}H\}$ NMR spectrum of **L5** in MeOD-d_4, 101 MHz (18 mM)

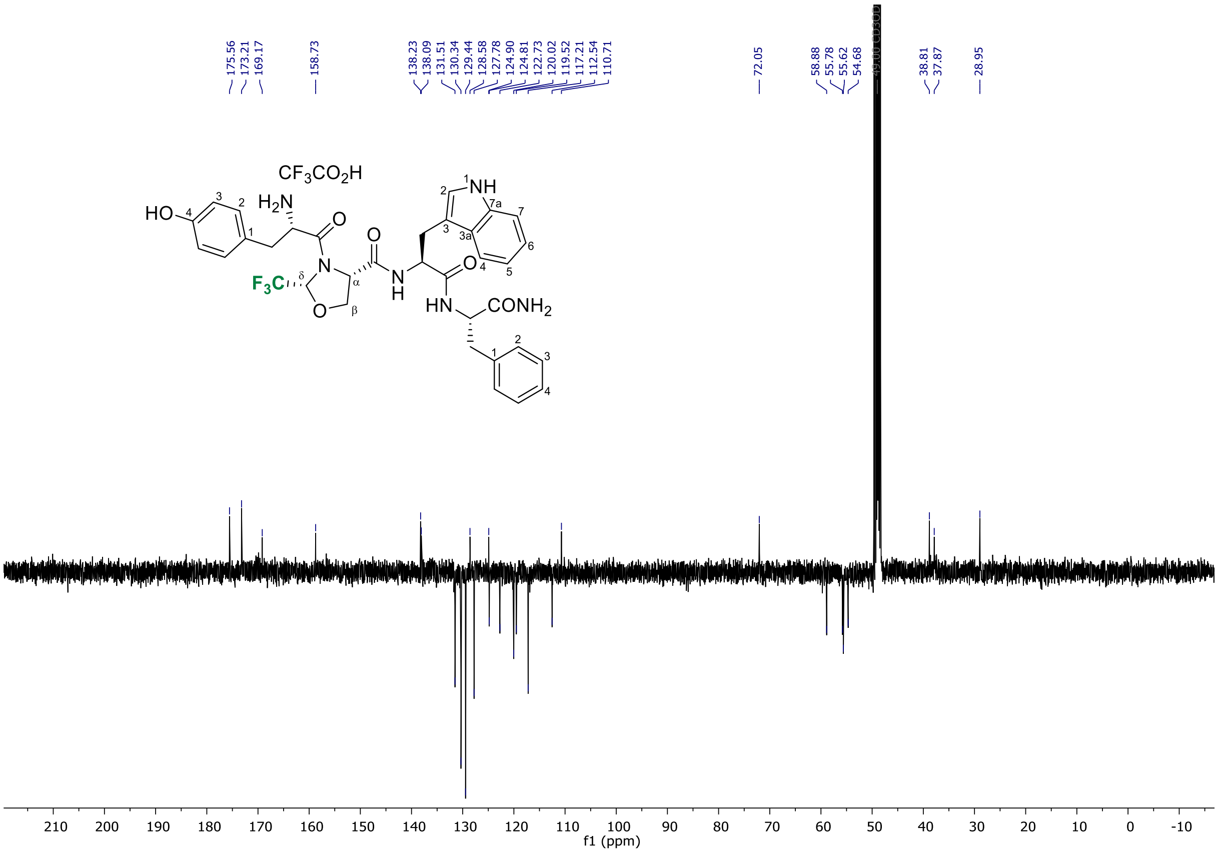

Figure S64: ^{19}F NMR spectrum of **L5** in MeOD-d_4, 376 MHz (18 mM)

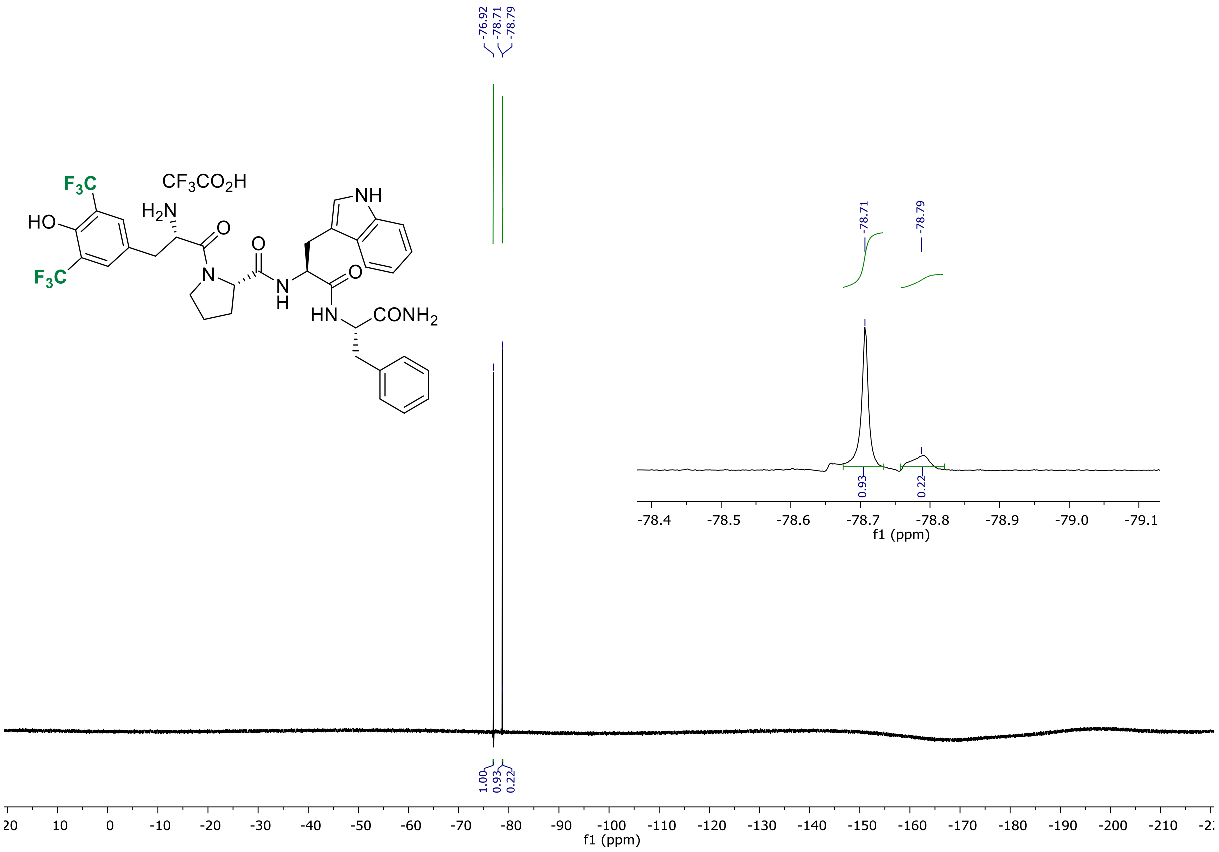

Figure S65: ^{1}H NMR spectrum of **L6** in MeOD-d_3, 400 MHz (20 mM, water suppression)

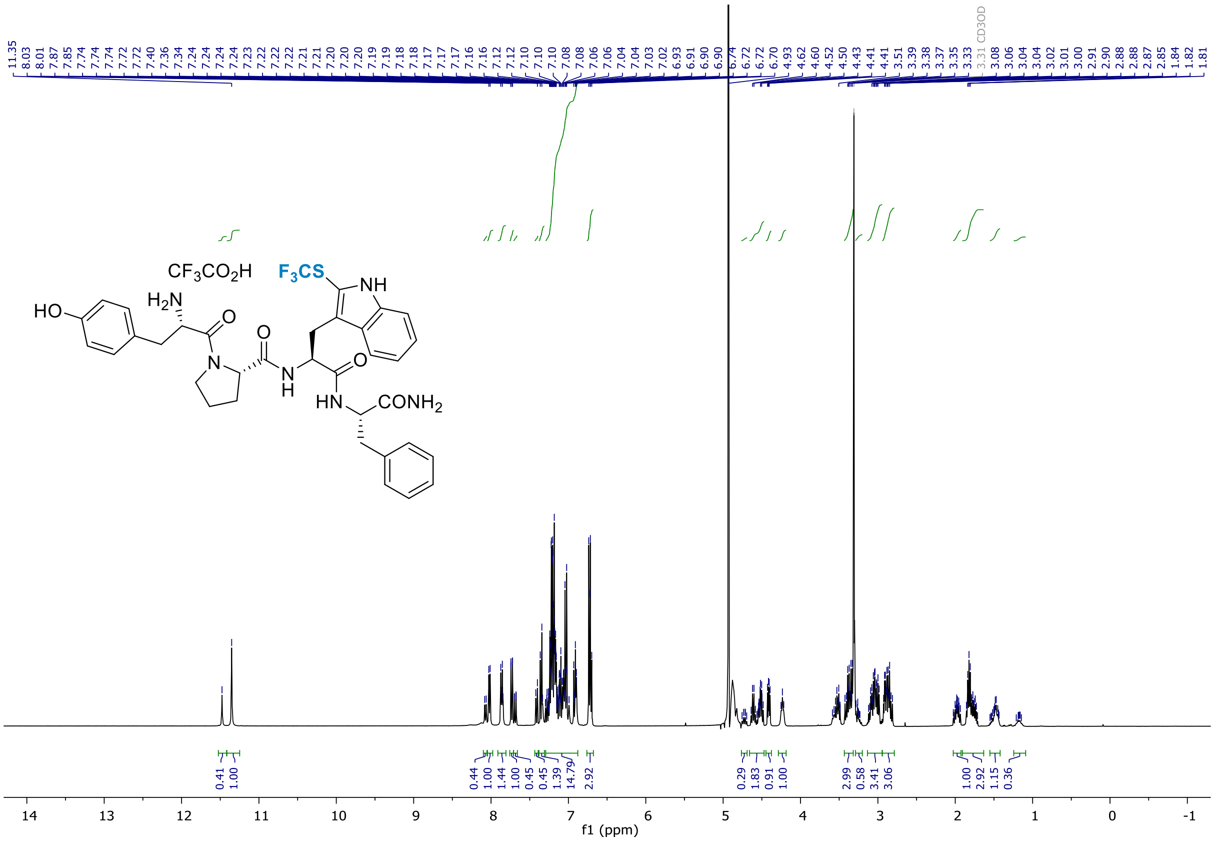

Figure S66: $^{13}C\{^1H\}$ NMR spectrum of **L6** in MeOD-d_3, 101 MHz (20 mM)

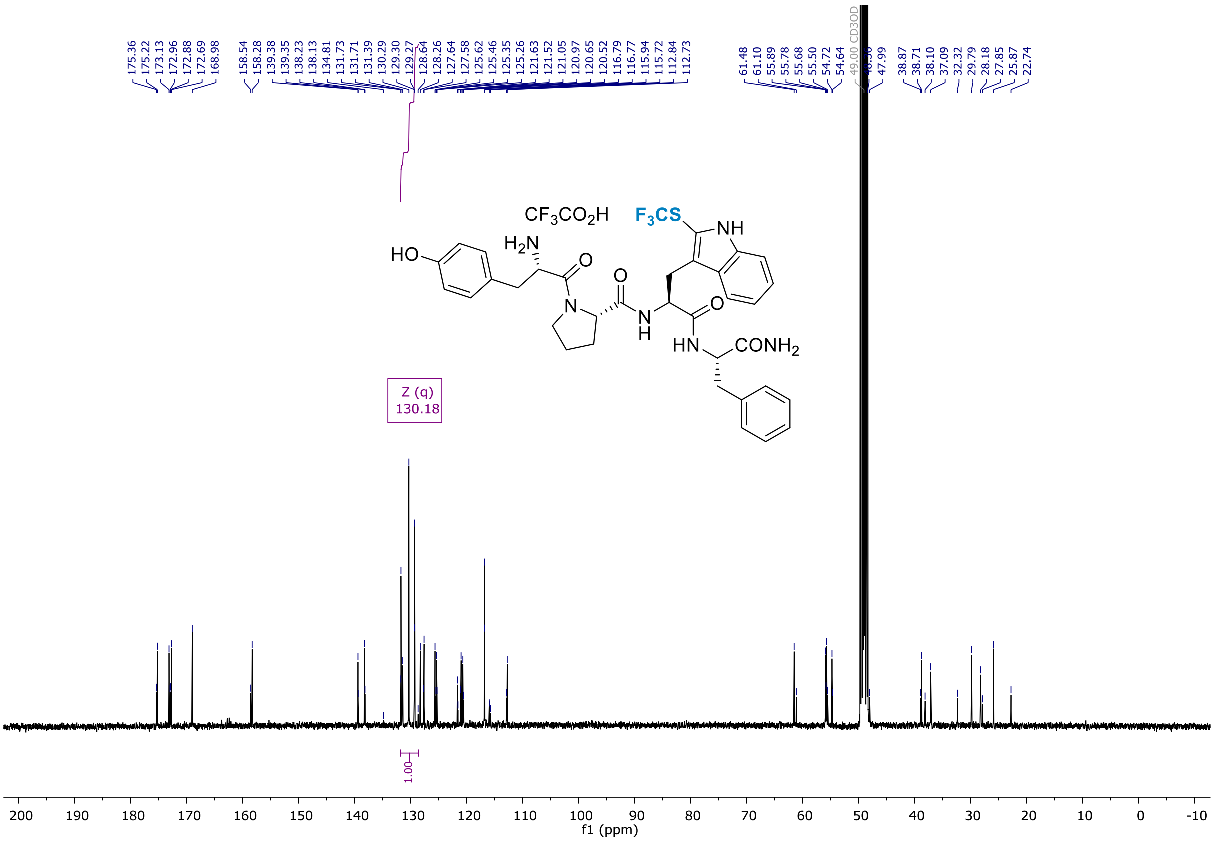

Figure S67: ^{19}F NMR spectrum of **L6** in MeOD-d_3, 376 MHz (20 mM)

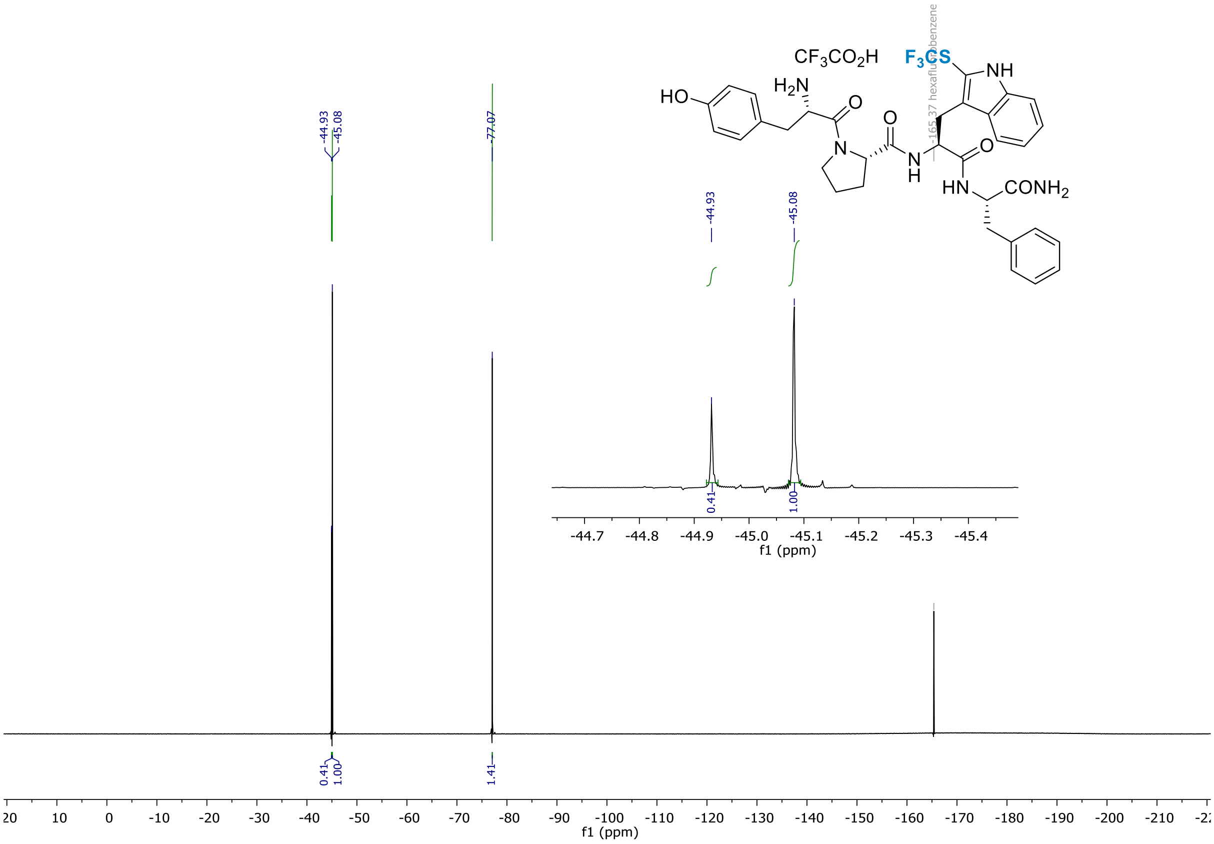

Figure S68: ^{1}H NMR spectrum of **L7** in MeOD-d_4, 600 MHz (20 mM)

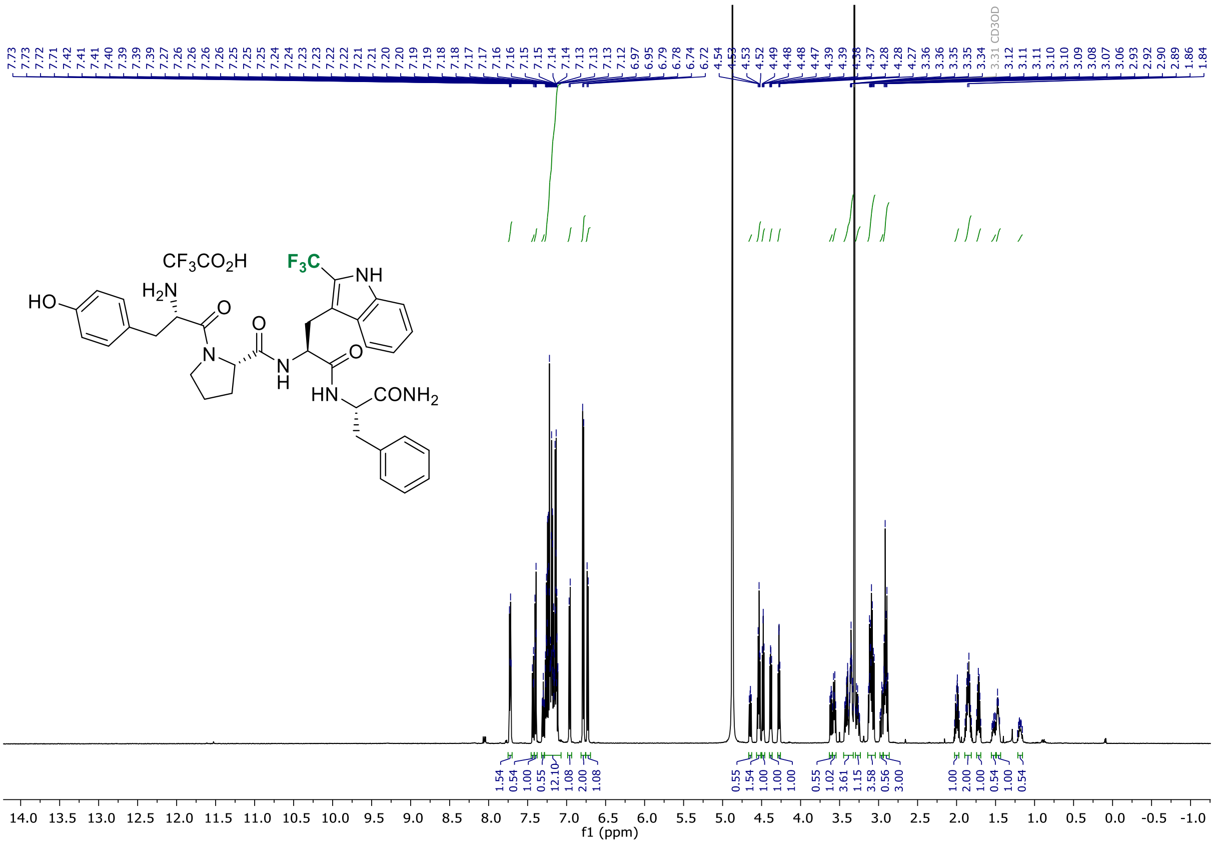

Figure S69: ^{13}C{^{1}H} spectrum of **L7** in MeOD-d_4, 151 MHz (20 mM)

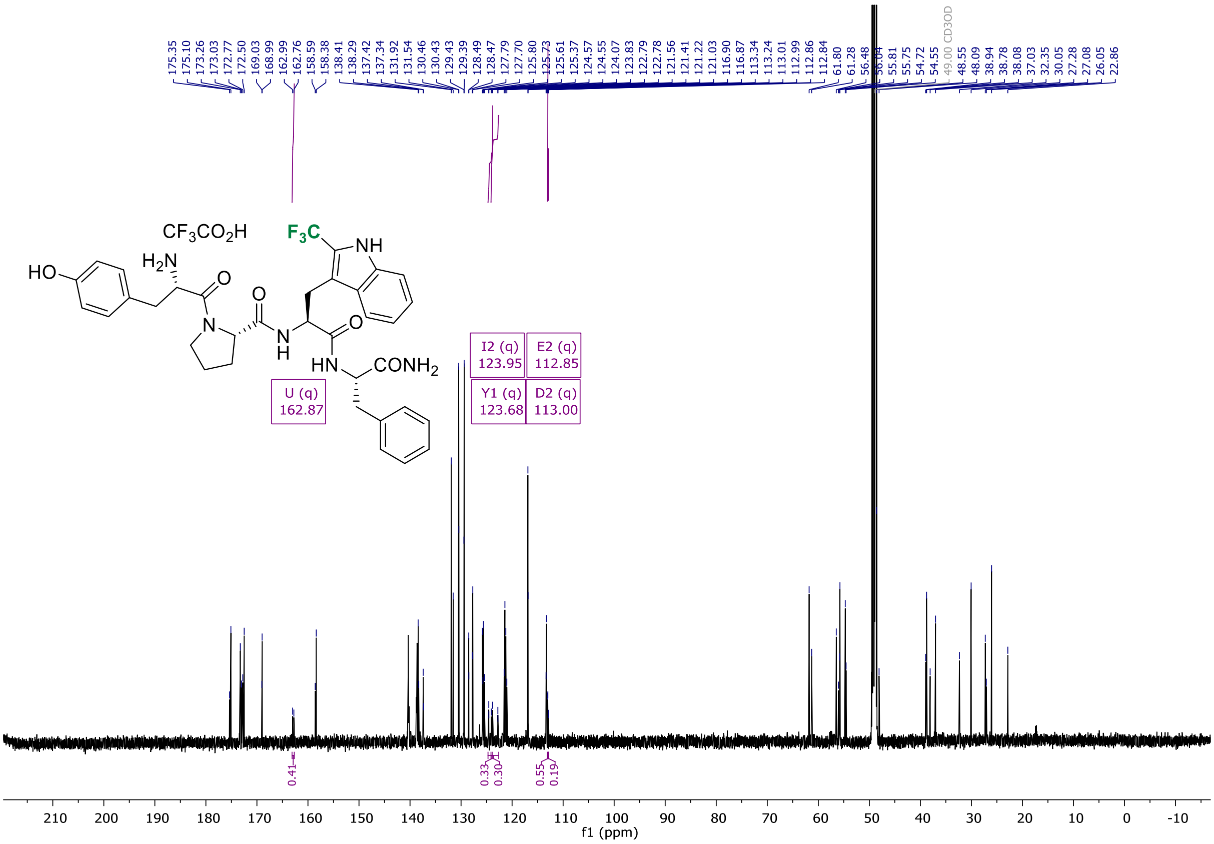

Figure S70: ^{19}F NMR spectrum of **L7** in MeOD-d_4, 565 MHz (20 mM)

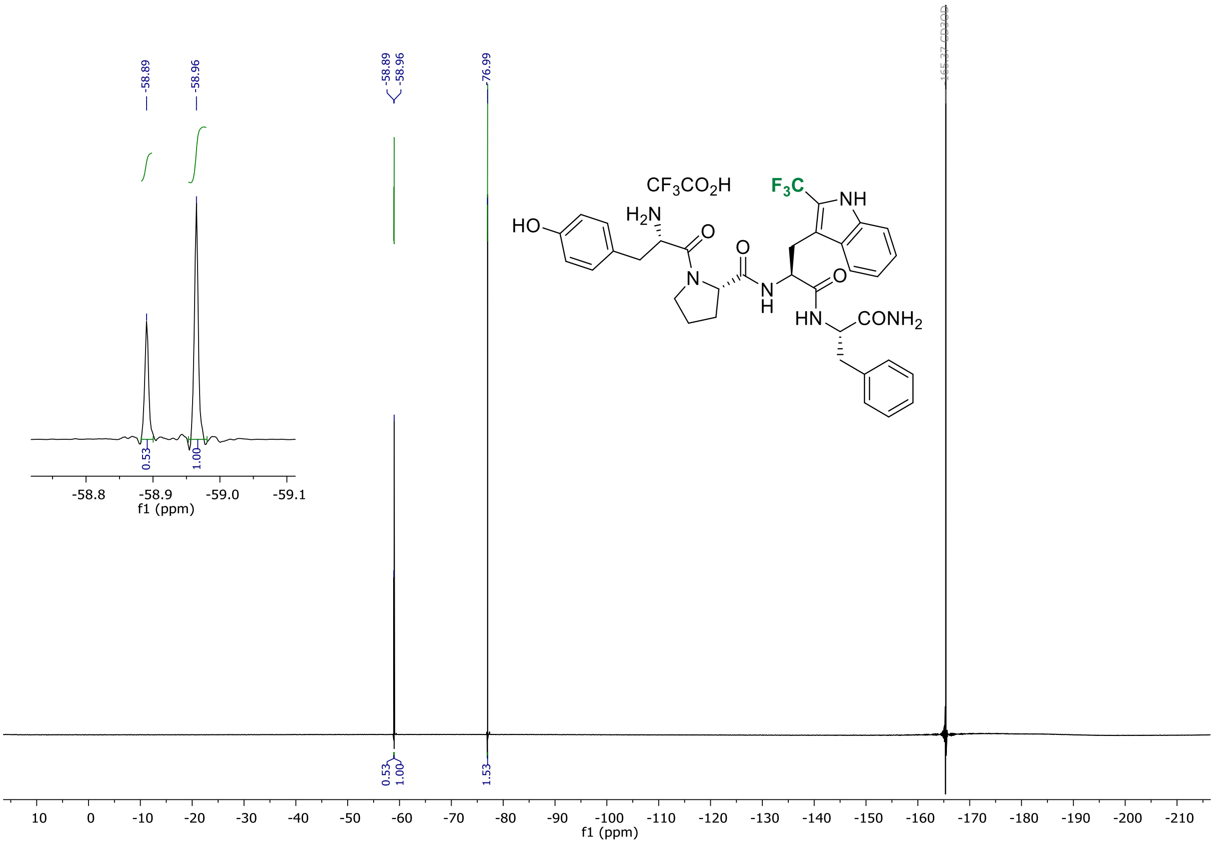

Figure S71: ^{1}H NMR spectrum of **L8** in MeOD-d_4, 600 MHz (10 mM)

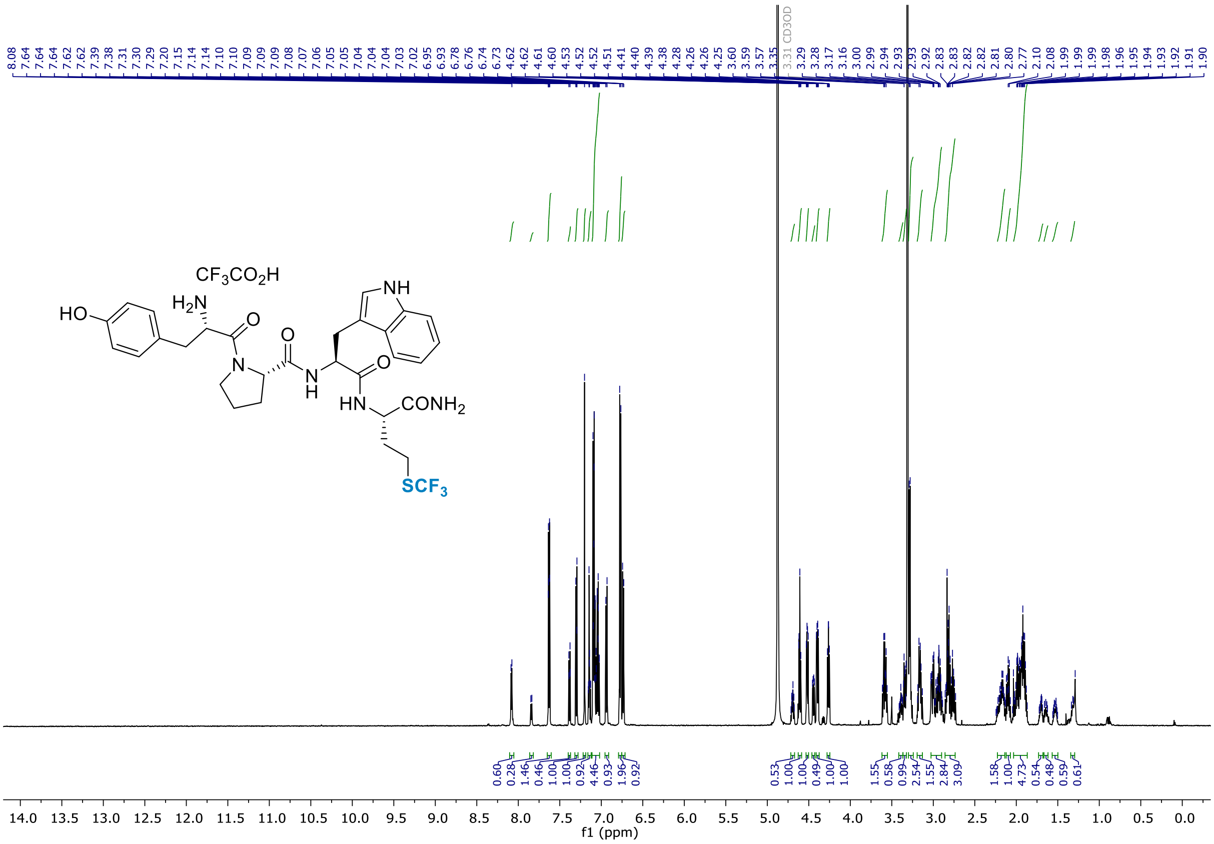

Figure S72: $^{13}C\{^{1}H\}$ spectrum of **L8** in MeOD-d_4, 151 MHz (10 mM)

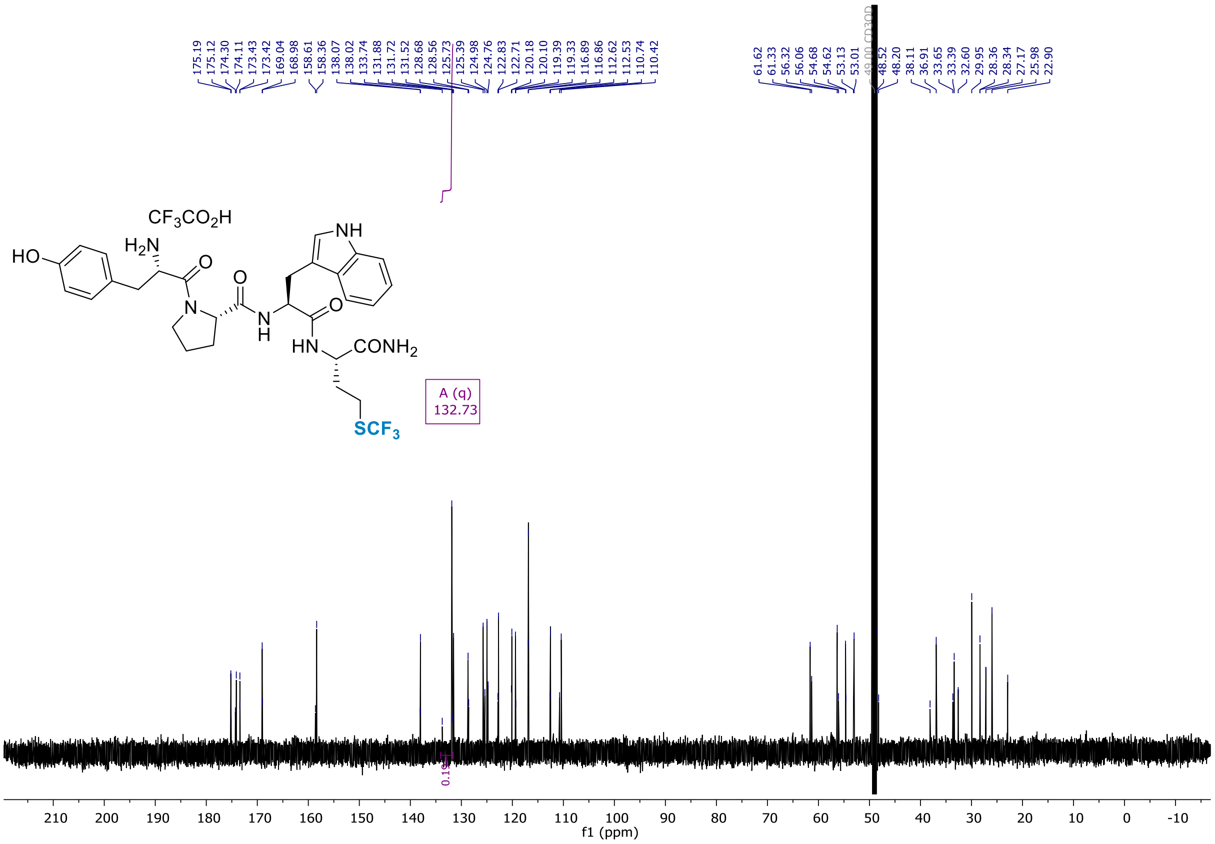

Figure S73: ^{19}F NMR spectrum of **L8** in MeOD-d_4, 565 MHz (10 mM)

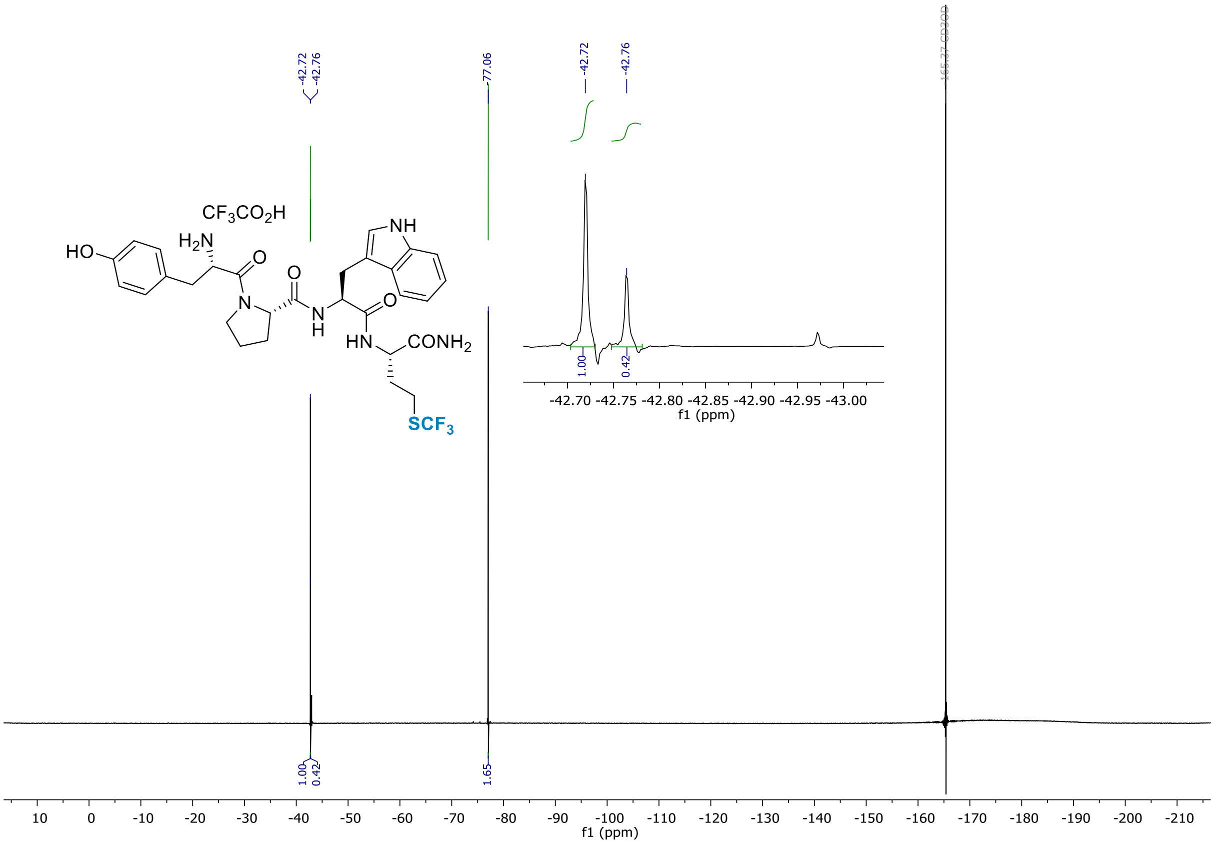

Figure S74: ^{1}H NMR spectrum of **L9** in MeOD-d_4, 400 MHz

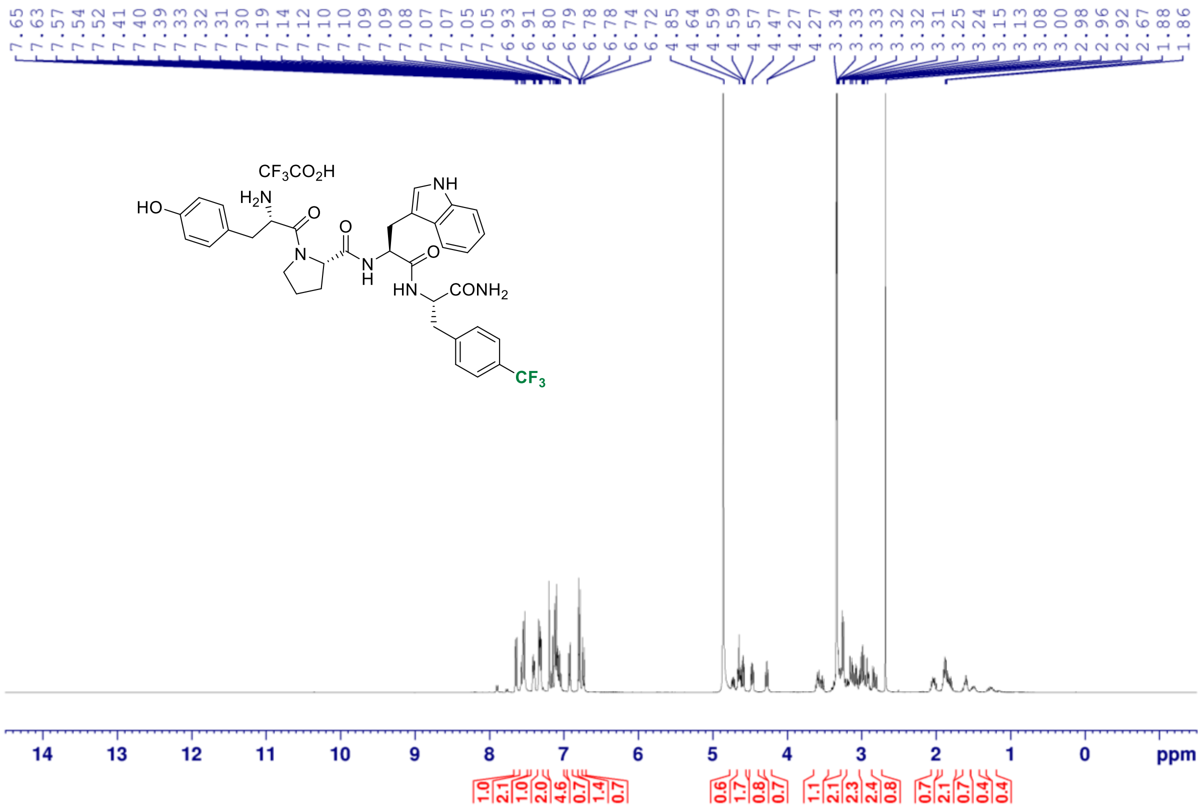

Figure S75: ^{19}F NMR spectrum of **L9** in MeOD-d_4, 376 MHz

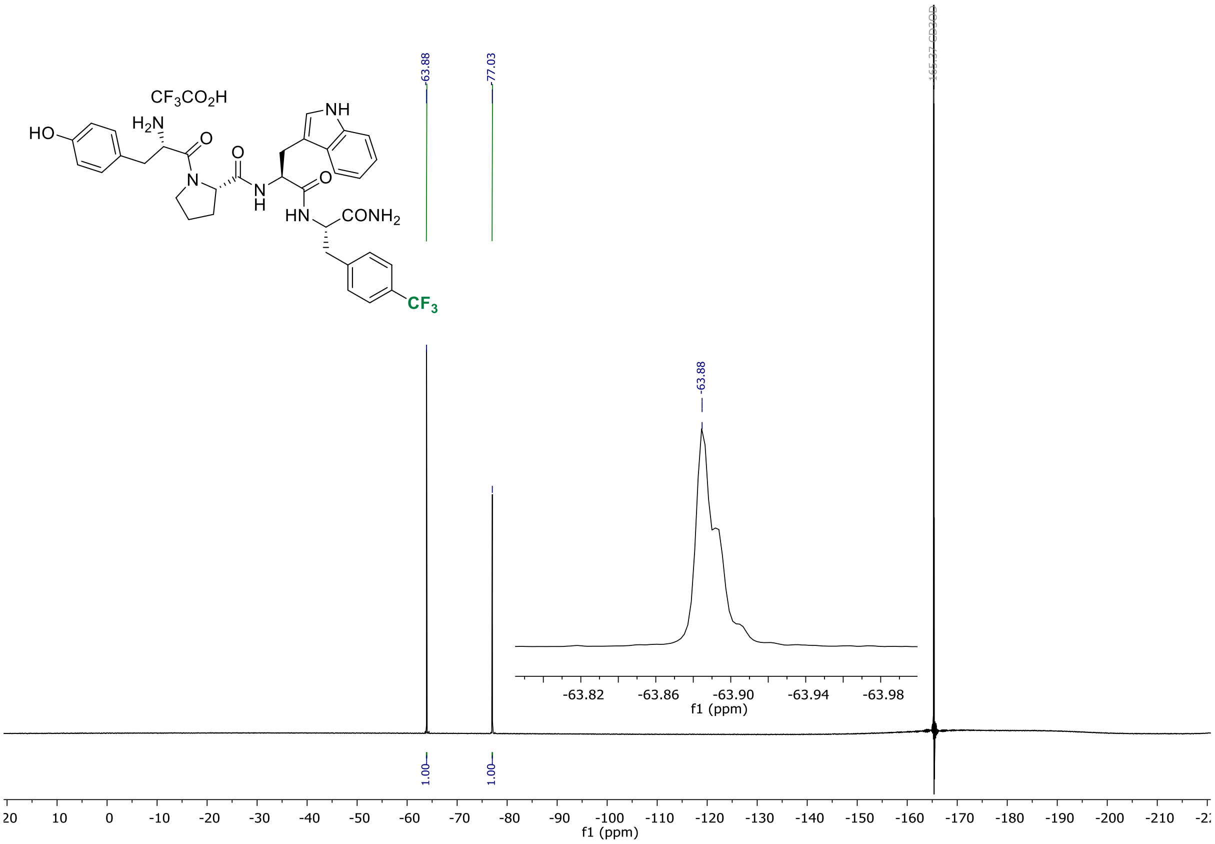

Figure S76: ^{1}H NMR spectrum of **L10** in MeOD-d_4, 400 MHz

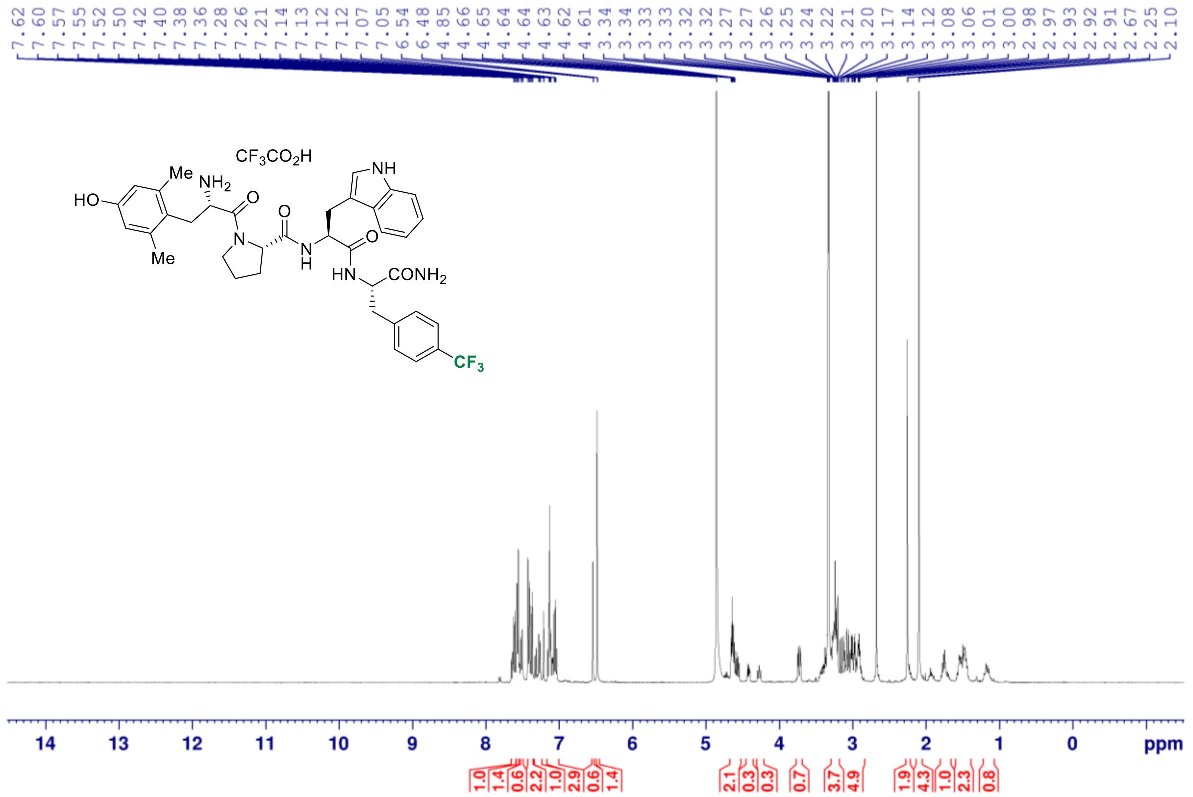

Figure S77: ^{19}F NMR spectrum of **L10** in MeOD-d_4, 376 MHz

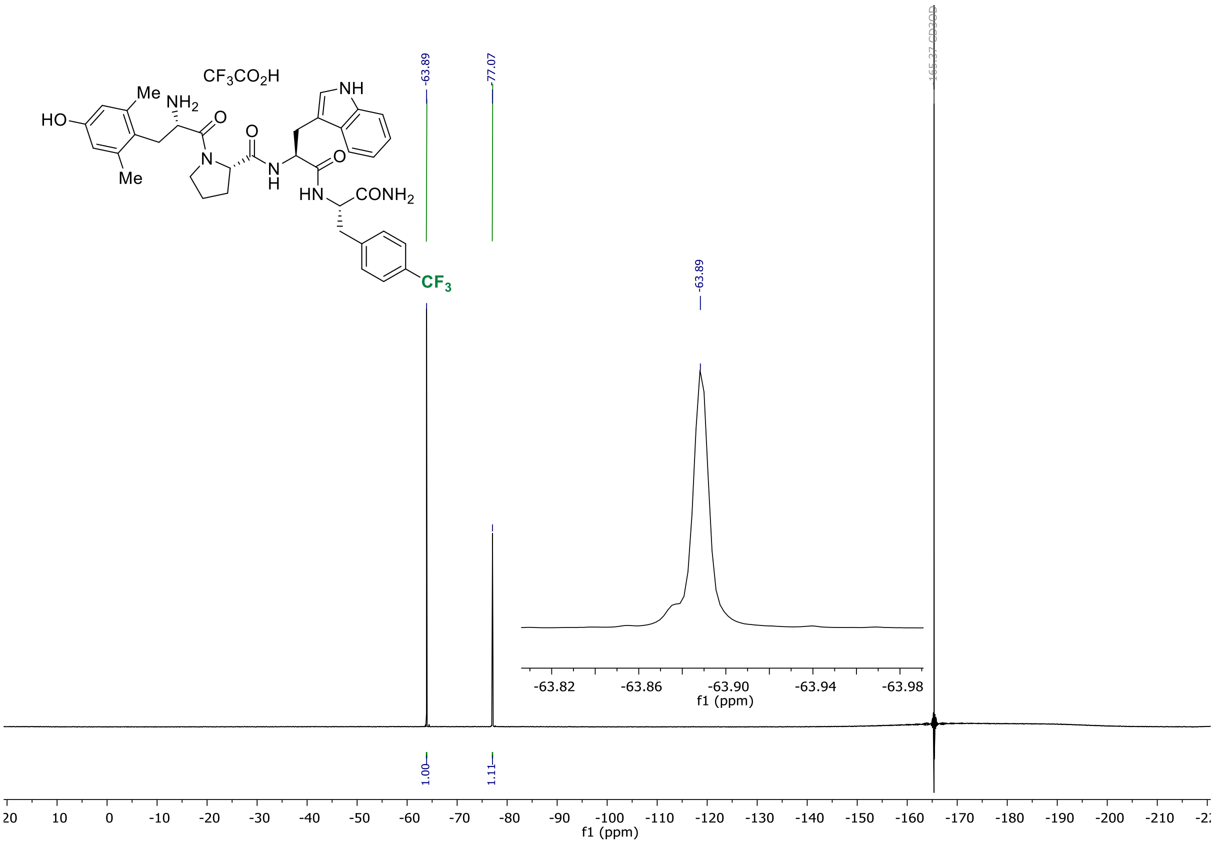

8. References

1. Rosenau, C. P.; Jelier, B. J.; Gossert, A. D.; Togni, A. Exposing the Origins of Irreproducibility in Fluorine NMR Spectroscopy. *Angew. Chem. Int. Ed.* **2018**, *57* (30), 9528–9533.
2. Engle, S. M.; Kirkner, T. R.; Kelly, C. B. Preparation of 2,4,5,6-Tetra(9*H*-carbazol-9-yl)isophthalonitrile. *Org. Synth.* **2019**, *96*, 455–473.
3. De Neve, J.; Breault, É.; Previti, S.; Vangeloven, E.; Loranger, B.; Chartier, M.; Brouillette, R.; Lanoie, A.; Holleran, B. J.; Longpré, J.-M.; Gendron, L.; Tourwé, D.; Sarret, P.; Ballet, S. Design, Synthesis, and *In Vitro* Characterization of Proteolytically-Stable Opioid-Neurotensin Hybrid Peptidomimetics. *ACS Pharmacol. Transl. Sci.* **2024**, *7* (9), 2784–2798.
4. Honfroy, A.; Bertouille, J.; Turea, A.-M.; Cauwenbergh, T.; Bridoux, J.; Lensen, N.; Mangialetto, J.; Van den Brande, N.; White, J. F.; Gardiner, J.; Brigaud, T.; Ballet, S.; Hernot, S.; Chaume, G.; Martin, C. Fluorinated Peptide Hydrogels Result in Longer *In Vivo* Residence Time after Subcutaneous Administration. *Biomacromolecules* **2024**, *25* (10), 6666–6680.
5. Chaume, G.; Barbeau, O.; Lesot, P.; Brigaud, T. Synthesis of 2-Trifluoromethyl-1,3-oxazolidines as Hydrolytically Stable Pseudoprolines. *J. Org. Chem.* **2010**, *75* (12), 4135–4145.
6. Ferry, A.; Billard, T.; Langlois, B. R.; Bacqué, E. Synthesis of Trifluoromethanesulfinamidines and -sulfanylamides. *J. Org. Chem.* **2008**, *73* (23), 9362–9365.
7. Gregorc, J.; Lensen, N.; Chaume, G.; Iskra, J.; Brigaud, T. Trifluoromethylthiolation of Tryptophan and Tyrosine Derivatives: A Tool for Enhancing the Local Hydrophobicity of Peptides. *J. Org. Chem.* **2023**, *88* (18), 13169–13177.
8. Clanton, N. A.; Spiller, T. E.; Ortiz, E.; Gao, Z.; Rodriguez-Poirier, J. M.; DelMonte, A. J.; Frantz, D. E. A Metal-Free Reductive *N*-Alkylation of Indoles with Aldehydes. *Org. Lett.* **2021**, *23* (9), 3233–3236.
9. Proteau-Gagné, A.; Bournival, V.; Rochon, K.; Dory, Y. L.; Gendron, L. Exploring the Backbone of Enkephalins To Adjust Their Pharmacological Profile for the δ-Opioid Receptor. *ACS Chem. Neurosci.* **2010**, *1* (11), 757–769.
10. Guerrero, I.; Correa, A. Cu-Catalyzed Site-Selective C(sp^2)–H Radical Trifluoromethylation of Tryptophan-Containing Peptides. *Org. Lett.* **2020**, *22* (5), 1754–1759.
11. Huang, P.; Lv, C.; Song, H.; Wang, C.; Du, J.; Li, J.; Sun, B.; Jin, C., An *in situ* generated proton initiated aromatic fluoroalkylation *via* electron donor–acceptor complex photoactivation. *Green Chem.* **2024**, *26* (12), 7198–7205.
12. Chaume, G.; Simon, J.; Caupène, C.; Lensen, N.; Miclet, E.; Brigaud, T. Incorporation of CF_3–Pseudoprolines into Peptides: A Methodological Study. *J. Org. Chem.* **2013**, *78* (20), 10144–10153.
